# Inhibiting the Histone Demethylase Kdm4a Restrains Cardiac Fibrosis After Myocardial Infarction by Promoting Autophagy in Premature Senescent Fibroblasts

**DOI:** 10.1002/advs.202414830

**Published:** 2025-04-15

**Authors:** Ming Jin, Chuling Li, Zhaoyi Wu, Zhenquan Tang, Jingfang Xie, Guoquan Wei, Zhiwen Yang, Senlin Huang, Yijin Chen, Xinzhong Li, Yanmei Chen, Wangjun Liao, Yulin Liao, Guojun Chen, Hao Zheng, Jianping Bin

**Affiliations:** ^1^ Department of Cardiology State Key Laboratory of Organ Failure Research Nanfang Hospital Southern Medical University Guangzhou 510515 China; ^2^ Guangdong Provincial Key Laboratory of Shock and Microcirculation Guangzhou 510515 China; ^3^ Cardiovascular Center the Sixth Affiliated Hospital School of Medicine South China University of Technology Foshan 528200 China; ^4^ Guangdong Provincial Geriatrics Institute Guangdong Provincial People's Hospital (Guangdong Academy of Medical Sciences) Southern Medical University Guangzhou 510080 China; ^5^ Department of Oncology Nanfang Hospital Southern Medical University Guangzhou 510515 China

**Keywords:** cardiac fibrosis, Kdm4a, premature senescent fibroblasts

## Abstract

Premature senescent fibroblasts (PSFs) play an important role in regulating the fibrotic process after myocardial infarction (MI), but their effect on cardiac fibrosis remains unknown. Here, the investigation is aimed to determine whether PSFs contribute to cardiac fibrosis and the underlying mechanisms involved. It is observed that premature senescence of fibroblasts is strongly activated in the injured myocardium at 7 days after MI and identified that Kdm4a is located in PSFs by the analysis of scRNA‐seq data and immunostaining staining. Moreover, fibroblast specific gain‐ and loss‐of‐function assays showed that Kdm4a promoted the premature senescence of fibroblasts and cardiac interstitial fibrosis, contributing to cardiac remodeling in the advanced stage after MI, without influencing early cardiac rupture. ChIP‐seq and ChIP‐PCR revealed that Kdm4a deficiency promoted autophagy in PSFs by reducing Trim44 expression through increased levels of the H3K9me3 modification in the Trim44 promoter region. Furthermore, a coculture system revealed that Kdm4a overexpression increased the accumulation of PSFs and the secretion of senescence‐associated secretory phenotype (SASP) factors, subsequently inducing cardiac fibrosis, which could be reversed by Trim44 interference. Kdm4a induces the premature senescence of fibroblasts through Trim44‐mediated autophagy and then facilitates interstitial fibrosis after MI, ultimately resulting in cardiac remodeling, but not affecting ventricular rupture.

## Graphical abstract

Kdm4a downregulation restrains excessive cardiac interstitial fibrosis and remodeling by depressing the premature senescence of fibroblasts in the advanced stage after MI but does not affect early ventricular rupture. It verified that Kdm4a downregulation promotes autophagy in premature senescent fibroblasts by increasing the H3K9m3 modification of the Trim44 promoter, significantly suppressing premature senescence in fibroblasts, and ultimately reducing cardiac interstitial fibrosis.

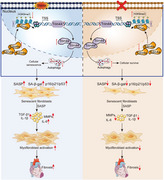

## 1 Introduction

Cardiac fibrosis, which is characterized by the transition of myofibroblasts from fibroblasts and excessive deposition of the extracellular matrix (ECM), is considered the major driver of adverse cardiac remodeling and progressive dysfunction in many heart diseases, including myocardial infarction (MI), chronic ischemic cardiomyopathy, genetic cardiomyopathies, and so on^[^ ^1^, ^2^ ^]^ It is widely known that cardiac fibrosis is a complex networking process involving many cellular and extracellular elements.^[^ ^3^, ^4^, ^5^, ^6^ ^]^ In recent decades, researchers have mainly focused on extracellular elements, and numerous extracellular factors, such as fibrogenic growth factors, cytokines, and neurohumoral factors have been identified as modulators of cardiac fibrosis.^[^ ^4^ ^]^ Several drugs targeting these extracellular factors, particularly ACEI/ARBs and β‐blockers, are currently used in clinical practice, but these treatments can only partially slow fibrotic progression in patients with cardiac diseases.^[^ ^5^ ^]^ Recently, increasing evidence has suggested that targeting cellular elements has greater efficiency in preventing or reversing fibrotic processes in multiple organs, including the heart.^[^ ^5^, ^6^, ^7^ ^]^ For example, valve endothelial cells have been reported to promote fibrosis in the cardiac mitral valve by transforming into fibroblasts through the endothelial‐to‐mesenchymal transition,^[^ ^8^ ^]^ while cardiac resident macrophages could prevent cardiac fibrosis in response to pressure overload.^[^ ^9^ ^]^ These findings suggest that cellular elements might play crucial roles in the fibrotic process in cardiac diseases and that the corresponding drug treatment might be an attractive strategy to prevent cardiac fibrosis and dysfunction.

Among these cellular elements, premature senescent cells were found that have a strong potential for regulating fibrotic processes in organs, including the lung,^[^ ^10^, ^11^, ^12^ ^]^ kidney^[^ ^13^ ^]^, and heart after injury.^[^ ^14^, ^15^, ^16^, ^17^ ^]^ Emerging evidence suggests that premature senescent cells also play a crucial role in promoting cardiac fibrosis.^[^ ^14^, ^15^ ^]^ Previous studies have demonstrated that premature senescent fibroblasts persistently exist in the injured myocardium and secrete cytokines, chemokines, and matrix‐remodeling enzymes, which are globally known as the senescence‐associated secretory phenotype (SASP).^[^ ^18^, ^19^, ^20^ ^]^ Secreted by premature senescent fibroblasts, the levels of SASP factors were reported to dramatically increase by 3–200 fold after injury.^[^ ^21^, ^22^ ^]^ It was also found that premature senescent fibroblasts play multiple and complex roles in the fibrotic process by secreting SASP factors in autocrine and paracrine manners.^[^ ^20^, ^23^ ^]^ First, premature senescent fibroblasts can directly promote the transition of myofibroblasts from fibroblasts and the deposition of ECM in fibrosis by secreting profibrotic factors and insoluble ECM proteins.^[^ ^11^, ^24^ ^]^ Moreover, premature senescent fibroblasts can maintain their own senescent status and induce the premature senescence of fibroblasts in damaged tissues in an autocrine manner, which contributes to the accumulation of premature senescent fibroblasts and further accelerates the process of fibrosis.^[^ ^11^, ^25^ ^]^ In addition, premature senescent fibroblasts can exert antiangiogenic effects on endothelial cells and activate neutrophils and macrophages to induce inflammation in a paracrine manner via SASP factors, eventually creating a profibrotic microenvironment.^[^ ^17^, ^24^ ^]^ The multiple effects and increased SASP factor secretion of premature senescent fibroblasts in the fibrotic process suggest that premature senescent fibroblasts might be extremely essential for mediating cardiac fibrosis. Therefore, determining the functions and key regulatory factors of premature senescent fibroblasts in injured myocardium is beneficial for the medical treatment of cardiac fibrosis after MI.

Since fibroblasts are a morphologically and functionally heterogeneous cell population,^[^ ^26^ ^]^ single‐cell RNA‐seq (scRNA‐seq) is an ideal method for precisely identifying the regulatory factors of premature senescent fibroblasts in the heart, which has also been applied to study the heterogeneity of fibroblasts in other organs.^[^ ^27^, ^28^ ^]^ After performing bioinformatics analysis of the scRNA‐seq data obtained from the MI model, we identified that the histone demethylase Kdm4a (also known as JMJD2A) is activated primarily in premature senescent fibroblasts from infarcted hearts. Kdm4a, which acts as an eraser of H3K9me3,^[^ ^29^, ^30^ ^]^ was reported to significantly exacerbate bleomycin‐induced lung and liver fibrosis by promoting the transition of myofibroblasts.^[^ ^31^, ^32^ ^]^ A recent study also revealed that Kdm4a could promote senescence in pancreatic cancer and nucleus pulposus cells.^[^ ^33^, ^34^ ^]^ Moreover, Kdm4a was found to orchestrate epigenomic remodeling in senescent cells, potentiating the SASP.^[^ ^29^ ^]^ The above results indicate the potent role of Kdm4a in regulating cellular senescence in cardiac fibrosis. Thus, we hypothesized that the histone demethylase Kdm4a induces the premature senescence of cardiac fibroblasts (CFs) by repressing cell autophagy, eventually facilitating cardiac fibrosis post‐MI.

In this study, on the basis of confirming the profibrotic effect of premature senescent fibroblasts in the injured heart, we demonstrated that Kdm4a downregulation restrains excessive cardiac interstitial fibrosis and remodeling by depressing the premature senescence of fibroblasts in the advanced stage after MI, but does not affect the early ventricular rupture. To further clarify the underlying mechanism of Kdm4a in premature senescent fibroblasts, we observed that Kdm4a can regulate H3K9me3 levels on tripartite motif containing 44 (Trim44), an important mediator of autophagy, by conducting ChIP‐seq assays and verified that Kdm4a downregulation promotes autophagy in premature senescent fibroblasts by increasing the H 3K9m3 modification of the Trim44 promoter, significantly suppressing premature senescence in fibroblasts, and ultimately reducing cardiac interstitial fibrosis.

## 2 Results

### 2.1 Premature Senescent Fibroblasts are Elevated and Persistent in the Heart Post‐MI

**Figure 1:**
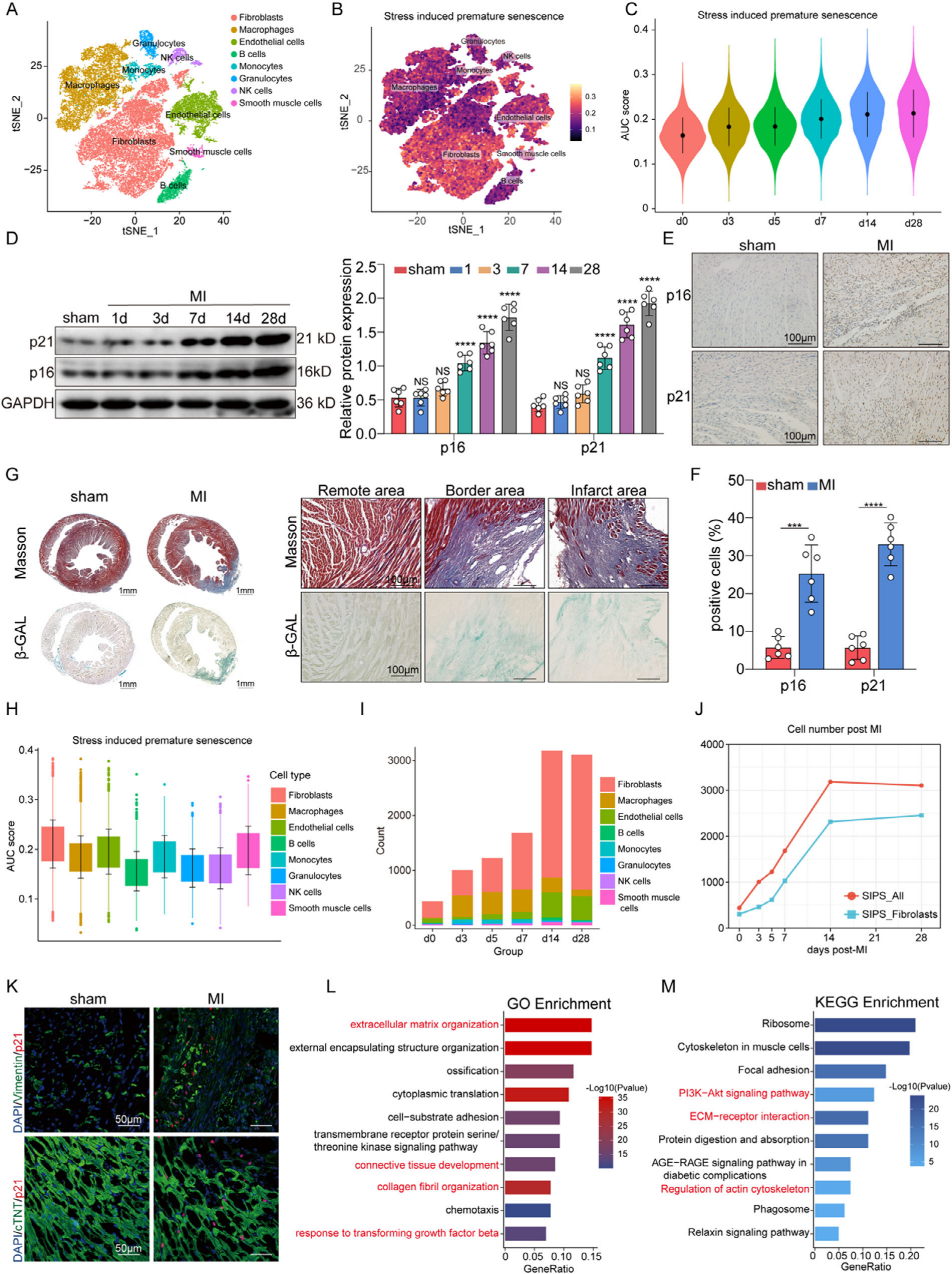
Premature senescent fibroblasts are elevated and persistent in the heart post‐MI A. A tSNE projection of cardiac cells analyzed by scRNA‐seq. Cells are colored according to the 8 major cell types. B. Heatmap illustrating the AUC scores of the SIPS gene set across different cell types, as projected on the tSNE plot. C. Violin plot illustrating the AUC scores of the SIPS gene set across various time points. D. Western blot analysis of the protein levels p16 and p21 in adult hearts after MI and in normal adult hearts. (*n* = 6/group) E‐F. Representative immunohistochemical staining and densitometric analysis of p16 and p21 in MI heart tissue compared to normal heart tissue. (*n* = 6 /group; scale bars = 100 µm) G. Representative images of Masson's trichrome staining and SA‐β‐gal staining in adult hearts harvested at 14 days post‐MI and in normal adult hearts. (*n* = 4 /group; scale bars = 100 µm) H. Box plot depicting the AUC scores of the SIPS gene set across various cell types. I. Bar plots showing the number of the eight‐cell types within the total SIPS cells at different time points. J. Line plot depicting the count of stress‐induced premature senescent cells and fibroblasts over different time points. K. Representative immunofluorescence staining for cTnT, α‐SMA, and p21 in the peri‐infarct zone at 14 days post‐MI and in normal adult hearts. (*n* = 6 /group; scale bars = 50 µm) L‐M. GO and KEGG pathway enrichment analysis of differentially expressed genes in SIPS fibroblasts compared to non‐SIPS fibroblasts. The x‐axis represents the gene ratio, while the y‐axis denotes the corresponding GO or KEGG terms. Data are expressed as the means SD for each group. D were analyzed by one‐way ANOVA followed by Tukey's test. F were analyzed by a two‐tailed unpaired t‐test. ^*^ *p* < 0.05, ^**^ *p* < 0.01, ^***^ *p* < 0.001, ^****^ *p* < 0.0001.

To investigate the potential correlation between premature senescence and the pathogenesis of MI, we first analyzed scRNA‐seq data from cardiac interstitial cells at homeostasis and 1, 3, 5, 7, 14, and 28days post‐MI obtained from ENA (accession code: E‐MTAB‐7895) and successfully captured 42049 cells (Figure S1A, Supporting Information). Based on the high expression of marker genes in each cluster, we manually annotated the captured cells into 8 major cell types, and the proportion of fibroblasts gradually increased at day 3 post‐MI (**Figure** **1**A; Figure S1B,C, Supporting Information). To elucidate stress‐induced premature senescence (SIPS) following MI, we defined a SIPS gene set comprising 58 genes identified from the CellAge database and found that SIPS activity gradually increased at 7 days and continued to increase until 28 days after MI through AUCell (Figure 1B,C). Based on the cut‐off (“Global_k1” value), we classified the cells into stress‐induced premature senescent (SIPS) cells and non‐SIPS cells, and the proportion of SIPS cells gradually increased at 7 days and continued to increase until 28 days after MI (Figure S1D–G, Supporting Information). The expression levels of the cellular senescence biomarkers p21 and p16 were further supported the fact that SIPS was gradually increased at 7 days and continued to increase until 28 days after MI (Figure 1D). Moreover, the histological analysis and senescence‐associated beta‐galactosidase (SA‐β‐Gal)‐positive cells were confirmed in both the infarct area and the border area through colocalization with Masson's trichrome staining (Figure 1E–G). Because fibroblasts are known to be the most abundant cells and located mainly in the infarct and border areas after MI,^[^ ^35^ ^]^ we next investigated whether premature senescent fibroblasts are the most abundant type of senescent cells in the infarct heart. Based on the AUC score of the SIPS gene set and the different cell types within SIPS cells, we found that fibroblasts were the predominant cell type following MI (Figure 1H,I). The number of SIPS fibroblasts gradually increased at 7 days following MI. Additionally, SIPS fibroblasts presented higher expression levels of cellular senescence biomarkers, including Cdkn1a, Cdkn2a, Glb1, and Trp53, compared to non‐SIPS fibroblasts (Figure 1J; Figure S1H, Supporting Information). We also performed flow cytometry to isolate senescent cells in cardiac tissue after MI and observed a progressive increase in the proportion of SIPS cells at 7 days post‐MI, with continued elevation until to 28 days, consistent with our analyses (Figure S1I, Supporting Information). Moreover, immunostaining also revealed that the senescent cells after MI were predominantly fibroblasts, as identified by costaining with vimentin and p21 (Figure 1K; Figure S1J, Supporting Information). Western blotting further showed that the expression of senescence‐related proteins increased significantly in fibroblasts collected from adult hearts at 7 days post‐MI (Figure S1K, Supporting Information). Furthermore, we performed an analysis of differentially expressed genes (DEG) between SIPS fibroblasts and non‐SIPS fibroblasts. Gene Ontology (GO) and Kyoto Encyclopedia of Genes and Genomes (KEGG) analyses revealed that the DEGs were associated with the regulation of extracellular matrix organization, collagen fibril organization, response to TGF‐β and extracellular matrix synthesis. Gene set enrichment analysis (GSEA) indicated that SIPS fibroblasts tend to promote extracellular matrix organization, collagen fibril organization, and cell‐substrate adhesion (Figure 1L,M; Figure S1L,M, Supporting Information). Collectively, these results indicated that the numbers of premature senescent cells, predominantly fibroblasts, are elevated and persistent in the heart following MI and that these premature senescent fibroblasts are closely associated with the development of cardiac fibrosis.

### 2.2 Kdm4a is Upregulated in Premature Senescent Fibroblasts After MI and is Associated with Cardiac Fibrosis

**Figure 2:**
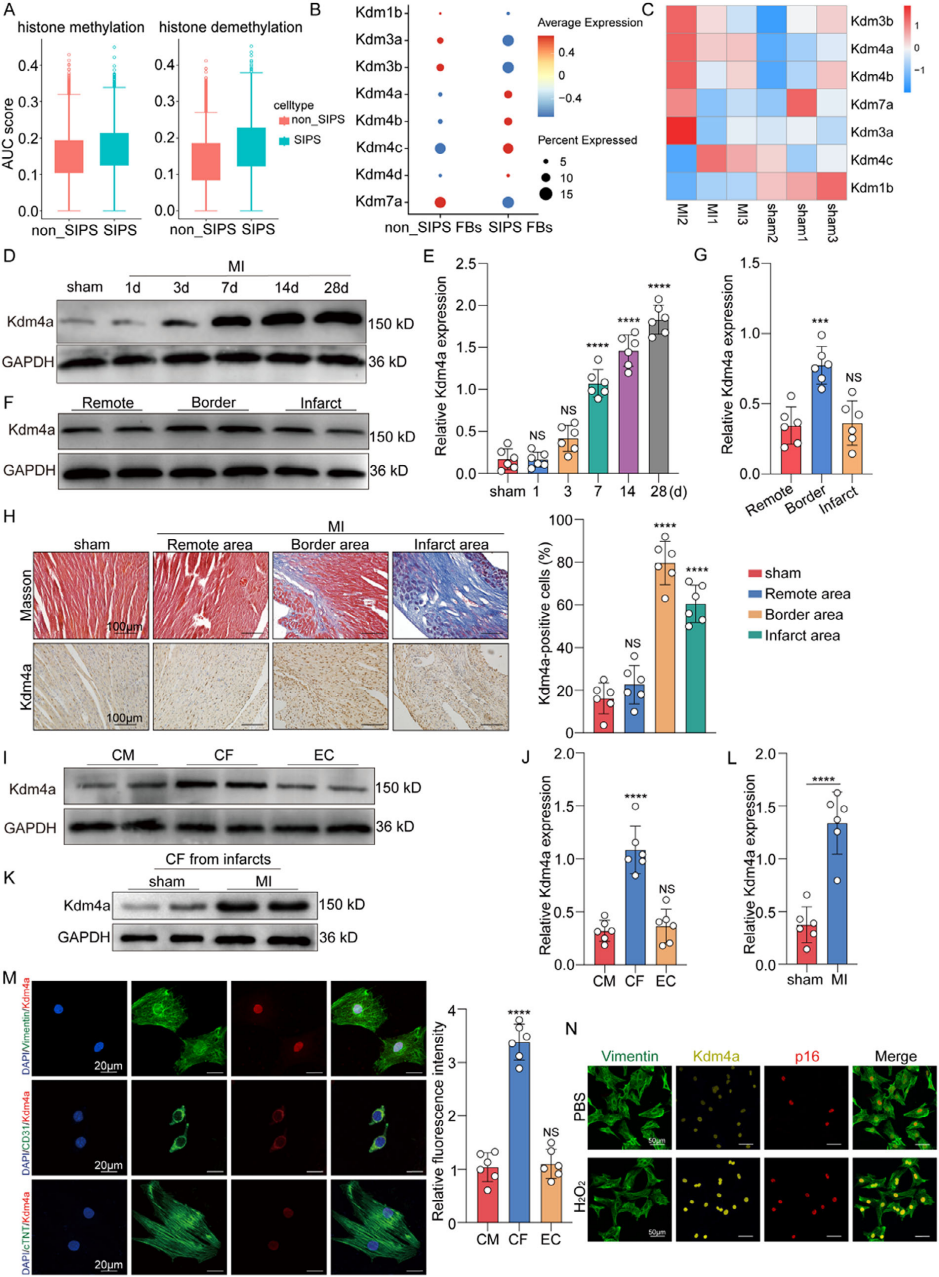
Kdm4a is upregulated in premature senescent fibroblasts after MI and H_2_O_2_‐induced fibroblasts A. Box plot depicting the AUC scores of the histone methylation and histone demethylation across various cell types. B. Dot plots showing the expression of Kdm family members in different fibroblast cell types. C. Heatmap showing the differential expression of Kdm family members between sham and MI (GSE183272). D‐G. Western blotting was used to detect the cardiac expression of Kdm4a and the relative levels of glyceraldehyde‐3‐phosphate dehydrogenase (GAPDH) were quantified in different groups. (*n* = 6/group) H. Representative images of Masson's trichrome staining and immunohistochemical staining of Kdm4a in different regions after MI. (*n* = 6 /group; scale bars = 100 µm) I‐J. Western blot analysis of Kdm4a protein levels in cardiomyocytes (CMs), fibroblasts (FBs) and endothelial cells (ECs). The cells were isolated from the hearts of mice with MI. (*n* = 6/group) K‐L. Western blot analysis of Kdm4a protein levels in isolated fibroblasts from the hearts of mice with MI and in control fibroblasts. (*n* = 6/group) M. Immunofluorescence staining analysis for CD31, vimentin and cTnT costaining to identify cells expressing Kdm4a in vitro. (*n* = 6 /group; scale bars = 20 µm) N. Immunofluorescence staining analysis for Kdm4a, p16 and vimentin in fibroblasts. (scale bars = 50 µm) Data are expressed as the means SD for each group. L was analyzed by two‐tailed unpaired t‐test. E, G, H, J and M were analyzed by one‐way ANOVA followed by Tukey's test. ^*^ *p* < 0.05, ^**^ *p* < 0.01, ^***^ *p* < 0.001, ^****^ *p* < 0.0001.

Histone methylation plays critical regulatory roles in gene expression and is involved in cellular differentiation and senescence,^[^ ^36^, ^37^ ^]^ which is regulated by histone methyltransferases and histone demethylases. To further investigate the relationship between histone methylation and cellular senescence, we analyzed the AUC scores of histone methylation and demethylation in senescent and non‐senescent cells post‐MI using single‐cell data. Our results revealed that senescent cells exhibited significantly higher histone demethylation scores, indicating a strong association between cellular senescence and histone demethylases (**Figure** **2**A). To elucidate the involvement of histone demethylases in senescence, we examined the expression of histone methylation‐related genes in senescent fibroblasts. Our analysis demonstrated that the histone demethylase KDM family, especially Kdm4a, was generally upregulated in premature senescent fibroblasts (Figure 2B), which was reported to play a pivotal role in various biological processes, including gene expression regulation, DNA repair, senescence, and cell fate determination. RNA sequencing data (GSE183272) also revealed that Kdm4a was strongly upregulated in the myocardium after MI (Figure 2C). Simultaneously, we established an in vitro model of premature senescent fibroblasts induced by H_2_O_2_. Fibroblasts were exposed to different concentrations of H_2_O_2_ (0, 50, 100, or 200 µm) and cultured for 96 h. The results showed that the number of SA‐β‐gal‐positive cells gradually increased in a concentration‐dependent manner and peaked at 100 µm H_2_O_2_ compared to controls (Figure S2A, Supporting Information). Notably, the CCK‐8 assays showed that H_2_O_2_ at a concentration of 100 µm did not induce significant cytotoxicity (Figure S2B, Supporting Information). Therefore, 100 µm H_2_O_2_ was selected for subsequent experiments (Figure S2C, Supporting Information). We assessed the mRNA levels of KDM family members in H_2_O_2_‐treated fibroblasts and various cardiac cell types isolated from mouse hearts (Figure S2D,E, Supporting Information). We found that Kdm4a was highly expressed in H_2_O_2_‐induced premature senescent fibroblasts. Given its strong expression, Kdm4a was selected for further analysis of its potential importance in cellular senescence. We also examined Kdm4a expression following MI. Western blotting revealed that Kdm4a levels gradually increased beginning at 7 days post‐MI and continued to increase until 28 days (Figure 2D,E). Furthermore, we found that Kdm4a was located mainly in the border zone and infarct zone rather than in the remote zone of adult hearts (Figure 2F–H). Western blotting further revealed that Kdm4a was predominantly expressed in fibroblasts after MI (Figure 2I,J). Next, we assessed Kdm4a expression in fibroblasts isolated from the infarcted myocardium, and found that the Kdm4a mRNA and protein levels were increased in fibroblasts from MI tissues (Figure 2K,L; Figure S2F, Supporting Information). Additionally, the immunofluorescence results confirmed that Kdm4a was predominantly localized in the nuclei of the fibroblasts and its expression was increased in the H_2_O_2_‐treated fibroblasts (Figure 2M,N). Overall, these findings suggest that Kdm4a is upregulated in response to cardiac injury and might play an important role in the premature senescence of fibroblasts and cardiac fibrosis after MI.

### 2.3 Knockdown of Kdm4a Ameliorates Cardiac Interstitial Fibrosis and Improves Cardiac Function After MI in Adult Mice

**Figure 3:**
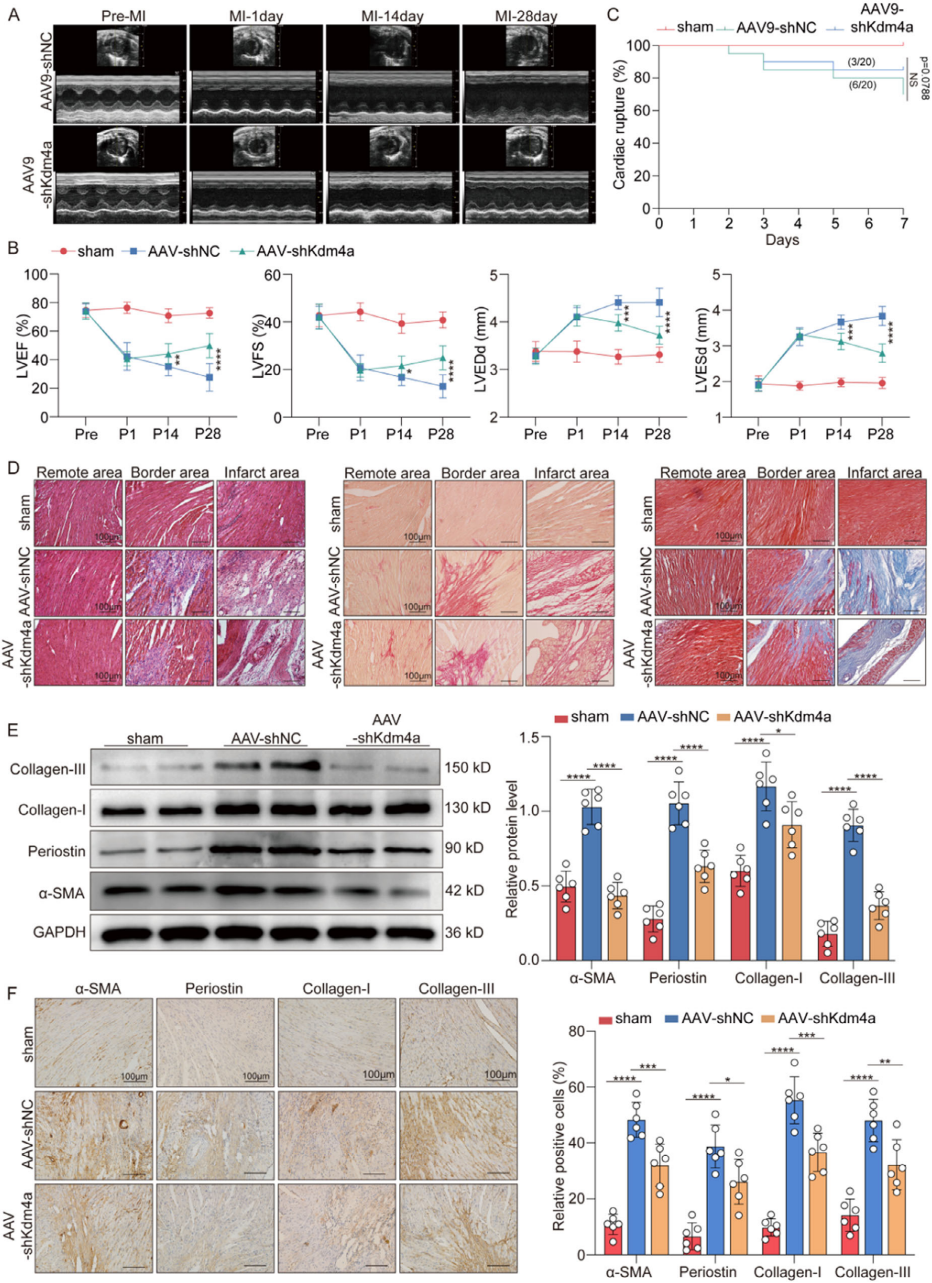
Knockdown of Kdm4a improved cardiac function and cardiac fibrosis after MI in adult mice A‐B. Cardiac function was assessed by echocardiography at pre‐MI and 1, 14, and 28 days after MI. (*n* = 10 /group) C. Percentage of cardiac rupture between mice injected with AAV9‐NC or AAV9‐shKdm4a post‐MI. (*n* = 20/group) D. The myocardium of three different zones in mice was harvested on day 14 post‐MI for the following study. Representative images of heamatoxylin and eosin (H&E), picrosirius red staining, and Masson's trichrome staining in the mouse heart. (*n* = 6 /group; scale bars = 100 µm) E. Western blot analysis of α‐SMA, periostin, collagenI, and collagenIII and GAPDH expression in heart tissues of mice and quantification for the indicated proteins. (*n* = 6 /group) F. Representative immunohistochemical staining of α‐SMA, periostin, collagen I, and collagen III in transverse sections from adult mouse MI model hearts at 14 days after MI. (*n* = 6 /group; scale bars = 100 µm) Data are expressed as the means SD for each group. B was analyzed by the two‐way ANOVA followed by Tukey's test. C was analyzed by the log‐rank (Mantel‐Cox) test. E and F were analyzed by one‐way ANOVA followed by Tukey's test. ^*^ *p* < 0.05, ^**^ *p* < 0.01, ^***^ *p* < 0.001, ^****^ *p* < 0.0001.

To determine the impact of Kdm4a deficiency on cardiac fibrosis after MI, AAV9 vector containing the shKdm4a gene with the fibroblast‐specific Postn promoter (AAV9‐Postn‐shKdm4a) was delivered into the myocardial tissue of adult mice to knock down Kdm4a, and AAV9‐Postn‐shNC served as the control. To assess the efficiency of AAV9 infection in fibroblasts, we isolated fibroblasts and cardiomyocytes from the hearts of adult mice 14 days after AAV9‐Postn‐GFP injection. Through colocalization analysis of the GFP, vimentin, and cTnT signals, we observed that ≈68% of vimentin‐positive cells exhibited GFP expression, with no significant GFP expression detected in cTnT‐positive cells (Figure S3A, Supporting Information). We found that the protein expression levels of Kdm4a were decreased in the AAV9‐Postn‐shKdm4a group compared with the AAV9‐Postn‐shNC group (Figure S3B, Supporting Information). These results suggest that the gene transfection system can effectively regulate the expression of Kdm4a in mouse fibroblasts. We further investigated the effects of Kdm4a on cardiac function. The results showed that cardiac function was significantly and equally decreased in all the MI groups compared with the sham group, and it was found that MI mice injected with the AAV9‐Postn‐shKdm4a exhibited marked improvements in the LVEF, LVFS, LVIDd and LVIDs compared with those injected with AAV‐scramble after MI surgery (**Figure** **3**A,B). In addition, AAV9‐Postn‐shKdm4a treatment significantly reduced the mortality caused by cardiac dysfunction in MI mice, but did not increase the risk of ventricular rupture in the first week after MI (Figure 3C; Figure S3C, Supporting Information). These results indicate that targeting Kdm4a can effectively alleviate adverse cardiac function after MI.

We further investigated the effects of Kdm4a on myocardial fibrosis after MI. Masson's trichrome staining revealed that Kdm4a knockdown reduced the size of the cardiac interstitial fibrotic area of the myocardium after MI compared to the control treatment (Figure S3D, Supporting Information). Furthermore, a comprehensive histological examination via hematoxylin and eosin (HE) staining, picrosirius red staining, and Masson's trichrome staining revealed that the fibrotic area in the infarct border zone was markedly reduced in the mice treated with AAV‐shKdm4a (Figure 3D; Figure S3E, Supporting Information). However, no discernible differences were observed between the infarct and remote zones in the three groups of mice. Concurrently, western blot and immunostaining assays revealed that the expression of α‐smooth muscle actin (α‐SMA), periostin, collagen I and collagen III was significantly increased in the MI mice injected with AAV‐shNC compared with the sham group. However, the expression of fibrotic markers was decreased in MI model mice injected with AAV‐shKdm4a compared with those injected with AAV‐shNC (Figure 3E,F). Immunofluorescence staining showed that Kdm4a knockdown effectively inhibited collagen III expression in MI model mice (Figure S3F). To further demonstrate Kdm4a function in vivo, we constructed CF‐specific Kdm4a knockout mice by injecting AAV‐sgRNA (Kdm4a) into adult mouse hearts with Cas9‐tdTomato expression (Figure S4A,B, Supporting Information). AAV‐sgRNA (Kdm4a) was successfully transduced and Kdm4a expression was significantly decreased (Figure S4C, Supporting Information). Masson's trichrome staining results revealed that CF‐specific Kdm4a deficiency effectively attenuated cardiac fibrosis formation after MI (Figure S4D, Supporting Information). Taken together, these findings strongly suggest that Kdm4a is a key driver of cardiac interstitial fibrosis post‐MI.

### 2.4 Kdm4a Knockdown Eliminates Premature Senescence in Fibroblasts both In Vitro and In Vivo

**Figure 4:**
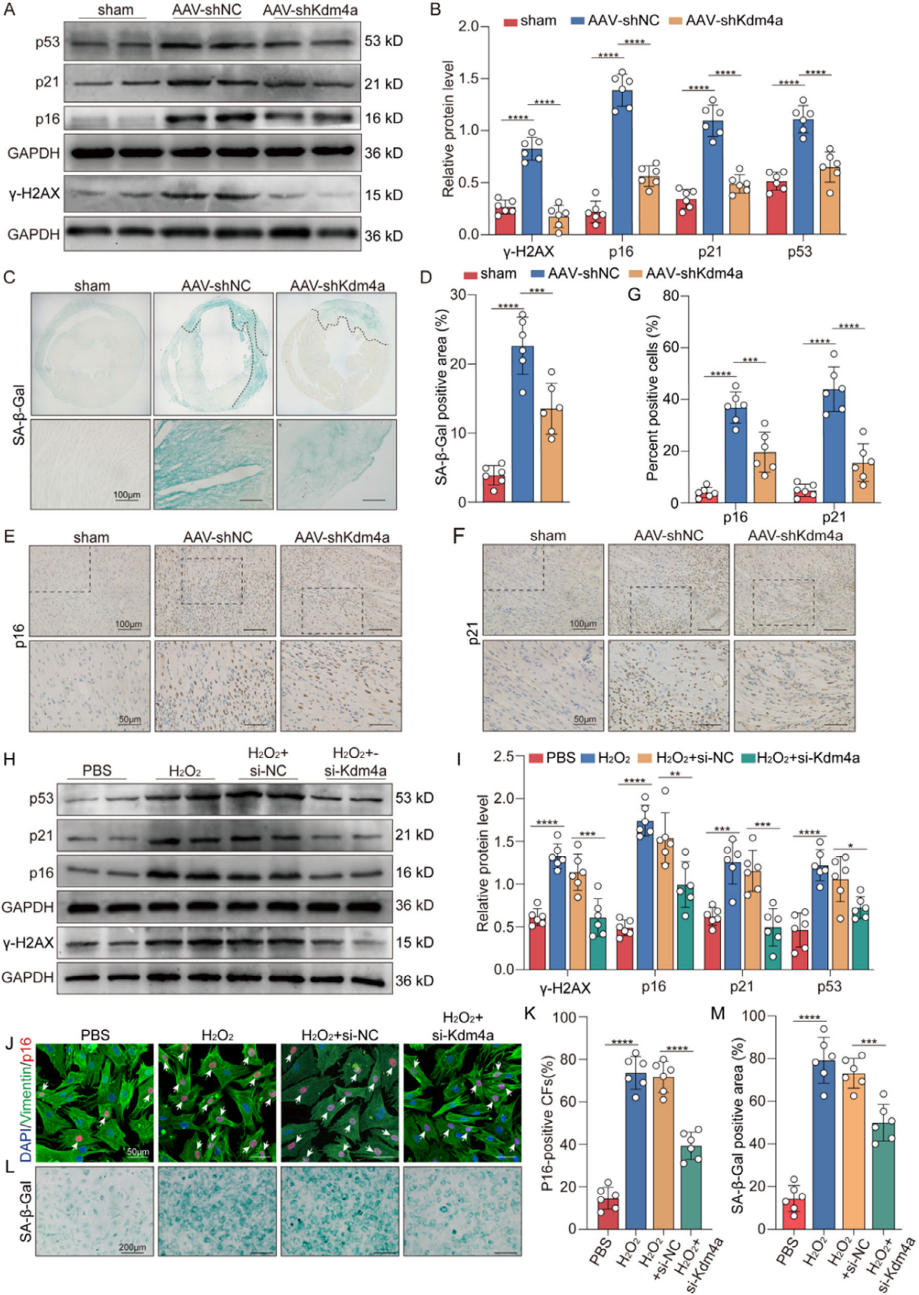
Deficiency of Kdm4a inhibits premature senescence of fibroblasts both in vitro and in vivo A‐B. Western blot analysis of p16, p21, p53 and γH2AX in cardiac tissue from mice harvested at 14 days post‐MI in each group. (*n* = 6 /group) C‐D. Representative images of SA‐β‐gal assay in the left ventricle of hearts harvested at 14 days post‐MI in different groups. (*n* = 6 /group; scale bars = 100 µm). E‐G. Representative densitometric analysis and immunostaining micrographs of senescence‐associated markers p16 and p21 in MI model hearts at 14 days post‐MI. (*n* = 6 /group; scale bars = 100 µm) H‐I. The mouse cardiac fibroblasts were pre‐treated with si‐Kdm4a and then exposed to H_2_O_2_ (100 µM) for an additional 96 h, and then the cells were analyzed. Western blotting and densitometric analysis of the protein levels of γH2AX, p16, p21 and p53 in each group. (*n* = 6 /group) J‐K. Representative immunofluorescence images for p16 in fibroblasts. Fibroblasts are stained with vimentin, nuclei are labelled with DAPI. (scale bars = 50 µm; *n* = 6/group) L‐M. Cytochemical evaluation of the SA‐β‐gal activity in fibroblasts and the percentage of positive fibroblasts was quantified. (scale bars = 200 µm; *n* = 6/group) Data are expressed as the means SD for each group. A‐M were analyzed by one‐way ANOVA followed by Tukey's test. ^*^ *p* < 0.05, ^**^ *p* < 0.01, ^***^ *p* < 0.001, ^****^ *p* < 0.0001.

An increasing body of evidence has indicated that accelerated cellular senescence is associated with cardiac fibrosis in MI mice.^[^ ^16^, ^18^ ^]^ We investigated whether the increase in the premature senescent fibroblasts following MI is responsible for the initiation of fibrosis. We subsequently assessed the effects of Kdm4a on cellular senescence. We detected the mRNA expression of p16, p21, and p53 and showed that Kdm4a knockdown significantly reduced the levels of these transcripts in the context of MI (Figure S5A, Supporting Information). Furthermore, qRT‐PCR analysis demonstrated that the expression of SASP markers, including IL‐6, IL‐10, IL‐1β, MMP2 and MMP9, decreased when Kdm4a was knocked down (Figure S5B, Supporting Information). These findings were further confirmed by western blot analysis (**Figure** **4**A,B). Additionally, Kdm4a suppression significantly decreased SA‐β‐gal activity in MI model mice (Figure 4C,D). Immunohistochemical assays showed that p21‐ and p16‐positive cells were less frequently stained in Kdm4a‐treated mice than in control mice (Figure 4E–G). We also evaluated the effect of senescent fibroblasts on cardiomyocytes, and the TUNEL staining results demonstrated that Kdm4a knockdown effectively inhibits cardiomyocyte apoptosis after MI (Figure S5C, Supporting Information). The results showed that inhibiting the senescence of fibroblasts could effectively suppress the activation of fibroblasts and the apoptosis of cardiomyocytes.

Next, we further assessed the impact of Kdm4a on cellular senescence in vitro. Fibroblasts were transfected with si‐Kdm4a and subsequently exposed to H_2_O_2_. Western blot and RT‒PCR assays were performed to evaluate the efficacy of Kdm4a knockdown in vitro (Figure S6A,B, Supporting Information). The results showed that H_2_O_2_ infusion increased the mRNA expression of senescence‐associated markers and SASP factors, whereas this effect was reversed when Kdm4a was knocked down (Figure S6C,D, Supporting Information). Additionally, the expression levels of senescence‐related proteins (p16, p21, p53 and γH2AX) were significantly increased in H_2_O_2_‐stimulated cells, which was alleviated by the transfection of the Kdm4a siRNA (Figure 4H,I). Immunofluorescence assays revealed decreased positive staining for p16 in myocardial fibroblasts subjected to si‐Kdm4a treatment (Figure 4J,K). Furthermore, SA‐β‐Gal staining indicated that the transfection of the Kdm4a siRNA significantly inhibited the H_2_O_2_‐induced activation of β‐galactosidase, whereas these changes were not observed in the NC group (Figure 4L,M). We then observed an increase in the levels of SASP factors in the supernatant of cardiac fibroblasts treated with H_2_O_2_, which was significantly attenuated by si‐Kdm4a (Figure S6E, Supporting Information). This effect was further verified by flow cytometry assays. The percentage of cells in G0/G1 phase decreased after transfection with si‐Kdm4a (Figure S6F, Supporting Information). These results indicate that the inhibition of Kdm4a can effectively prevent the premature senescence of fibroblasts and the SASP both in vitro and in vivo.

### 2.5 Kdm4a Knockdown Suppresses Fibroblast Activation Induced by Premature Senescent Fibroblasts

**Figure 5:**
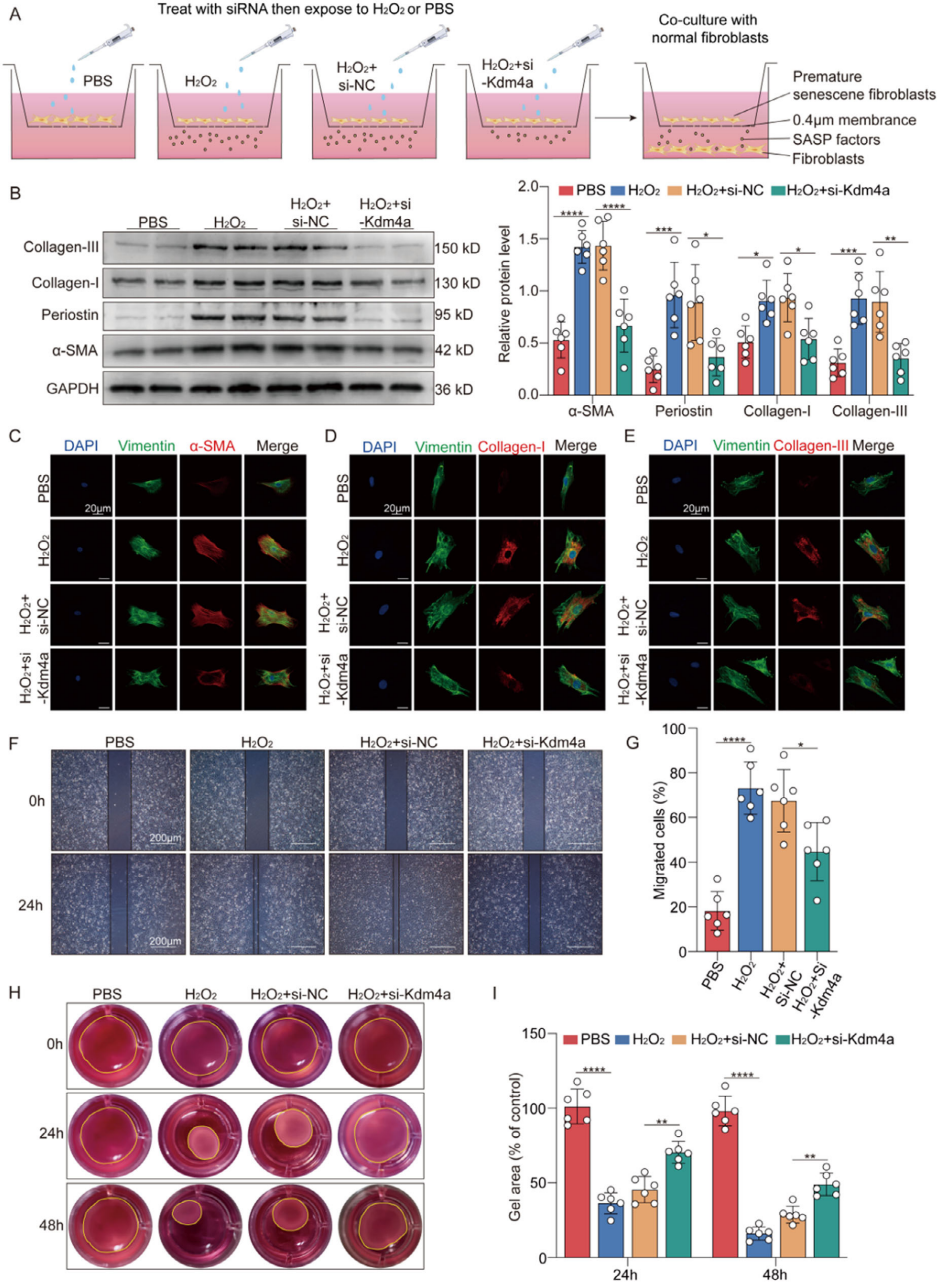
Kdm4a deficiency inhibits H_2_O_2_‐induced cardiac fibroblast activation and extracellular matrix (ECM) production A. Schematic diagram of transwell support coculture experiments to assess the effect of H_2_O_2_‐induced senescent fibroblasts on normal fibroblasts. B. Western blotting of the protein levels of fibrotic markers α‐SMA, periostin, collagen I, and collagen IIII in cultured fibroblasts transfected with siRNA against Kdm4a (si‐Kdm4a). (*n* = 6 /group) C–E. Representative immunofluorescence images for α‐SMA (red) (C), collagen I (red) (D), and collagen III (red) (E) in fibroblasts. Fibroblasts are stained with vimentin, nuclei are labeled with DAPI. (scale bars = 20 µm) F‐G. Representative images showing the scratch wound assay in fibroblasts transfected with si‐Kdm4a. Images of the wounds were captured at 0 and 24 h after the assay. The area between the wound edges was measured and compared between the groups. (scale bars = 200 µm; *n* = 6 /group) H‐I. Collagen gels containing fibroblasts were photographed at 0, 24, and 48 h. The sizes of the gels were measured and analyzed to assess changes in gel. Data are expressed as the means SD for each group. B and G were analyzed by one‐way ANOVA followed by Tukey's test. I was analyzed by the two‐way ANOVA followed by Tukey's test. ^*^ *p* < 0.05, ^**^ *p* < 0.01, ^***^ *p* < 0.001, ^****^ *p* < 0.0001.

Cellular senescence is considered an accelerator of the functional deterioration of organs.^[^ ^16^ ^]^ To further confirm whether premature senescent fibroblasts can stimulate the activation of normal fibroblasts, we established a coculture system utilizing the Boyden chamber system to coculture normal fibroblasts with premature senescent fibroblasts (**Figure** **5**A). Interestingly, we found that H_2_O_2_‐treated senescent fibroblasts increased the expression of fibrosis‐related indicators, whereas this effect was abolished when the fibroblasts were preincubated with si‐Kdm4a (Figure 5B). Immunocytological staining revealed that H_2_O_2_ treatment significantly induced the expression of α‐SMA, collagen I, and collagen III. However, transfection with Kdm4a siRNA significantly inhibited the expression of these proteins (Figure 5C–E; Figure S7A, Supporting Information). Wound‐healing assays were performed to assess the migratory behavior of normal fibroblasts cocultured with H_2_O_2_‐induced premature senescent fibroblasts. Notably, the results demonstrated that si‐Kdm4a pretreatment significantly inhibited the migration of normal fibroblasts cocultured with H_2_O_2_‐induced premature senescent fibroblasts (Figure 5F,G). The collagen lattice contraction assay is a well‐established method for assessing fibroblast contractility. The results revealed a notable reduction in collagen contractility in the Kdm4a‐knockdown group compared with the control group (Figure 5H,I).

To further demonstrate that Kdm4a regulates fibroblast activation by affecting premature fibroblast senescence, we performed rescue experiments. We overexpressed Kdm4a to induce premature senescence in cardiac fibroblasts, followed by treatment with ABT263,^[^ ^38^ ^]^ a potent senolytic drug used to selectively eliminate senescent cells. Additionally, we performed a coculture experiment in which the treated fibroblasts were cocultured with untreated normal fibroblasts. Western blot analysis revealed that ABT263 treatment significantly decreased the expression of Kdm4a‐driven senescence‐related proteins in treated fibroblasts and fibrosis‐related proteins in normal fibroblasts (Figure S8A,B, Supporting Information). Furthermore, we used Ang II to induce fibroblast activation, establishing an in vitro fibrosis model, while simultaneously knocking down Kdm4a. Western blot analysis revealed that si‐Kdm4a partially inhibited fibroblast activation, but its effect was relatively weak (Figure S8C,D, Supporting Information). Taken together, these findings suggest that si‐Kdm4a inhibits the activation of cardiac fibroblasts mainly by its mediation of premature senescent fibroblasts.

Next, we investigated the role of Kdm4a in human cardiac fibroblasts. Western blot and immunofluorescence analyses demonstrated that Kdm4a knockdown effectively reduced the expression of senescence‐associated proteins in human cardiac fibroblasts (Figure S9A,B, Supporting Information). Furthermore, when senescent fibroblasts were co‐cultured with normal human fibroblasts, western blot, and immunofluorescence results indicated that Kdm4a knockdown significantly decreased the expression of fibrosis‐related proteins in human cardiac fibroblasts (Figure S9C,D, Supporting Information). These findings suggest that Kdm4a knockdown mitigates cardiac fibrosis by suppressing senescent fibroblast in human cardiac fibroblasts.

### 2.6 Kdm4a Knockdown Increases the Deposition of H3K9me3 at the Promoter of Trim44 in Premature Senescent Fibroblasts

**Figure 6:**
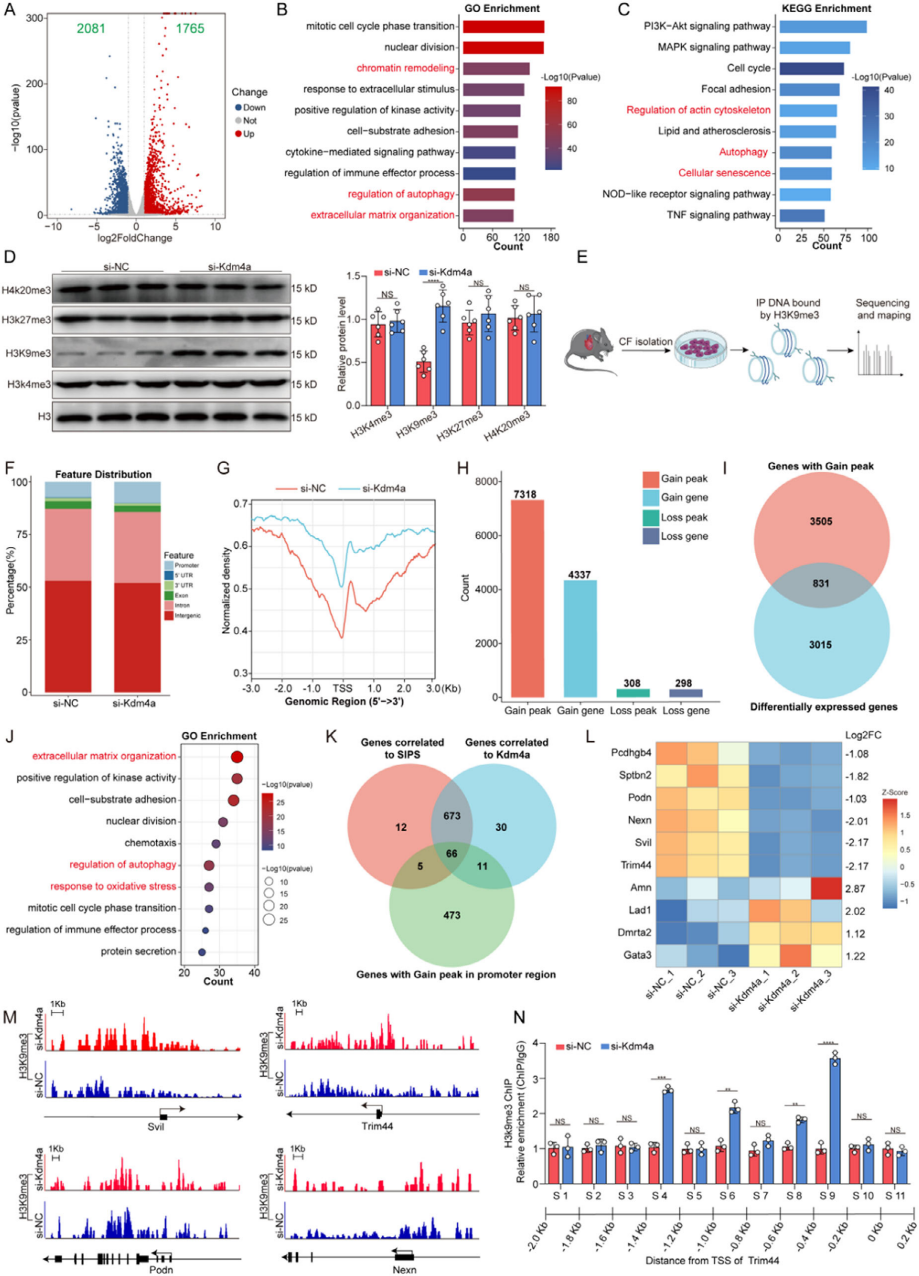
Kdm4a knockdown suppresses Trim44 expression by directly enhancing H3K9me3 deposition A. Volcano plot illustrating differentially expressed genes in adult fibroblasts incubated with H_2_O_2_+si‐NC or H_2_O_2_+si‐Kdm4a. A |log2FC|>1 and a *p* value <0.05 were considered indicative of significant differential expression. B‐C. GO and KEGG pathway enrichment analysis of differentially expressed genes after Kdm4a knockdown. The x‐axis indicates the gene count, and the y‐axis specifies the GO or KEGG terms. D. Western blot analysis was performed to show the global changes of histone methylation modifications induced by Kdm4a knockdown in fibroblasts. (*n* = 6 /group) E. Schematic diagram to illustrate the workflow of H3K9me3 ChIP‐seq experiments. F. Bar chart showing the genomic distribution of H3K9me3 binding peaks in H_2_O_2_‐treated fibroblasts transfected with either si‐NC or si‐Kdm4a, including promoters, 5′ UTR, 3′ UTR, exons, introns, and intergenic regions. G. Normalized ChIP‐seq density was assessed in H_2_O_2_‐treated fibroblasts transfected with either si‐NC or si‐Kdm4a. The signal intensities were ranked according to H3K9me3 read density within ±3 kilobases of the peaks relative to the transcription start sites. H. Bar plot illustrating the count of differentially enriched peaks and their annotated genes in H_2_O_2_‐treated fibroblasts following Kdm4a knockdown. Differentially expressed peaks were identified with a |log2FC|>1 and a p value <0.05 were considered to indicate statistical significance. I. Venn diagram illustrating the 831 genes that overlap between those with elevated H3K9me3 modification levels, as assessed by ChIP‐seq, and those identified as differentially expressed through RNA‐seq in H_2_O_2_‐treated fibroblasts following Kdm4a knockdown. J. GO enrichment analysis of the overlapping correlated to the ssGSEA scores of SIPS. The x‐axis indicates the gene count, and the y‐axis specifies the GO terms. K. Venn diagram depicting the intersection among 831 genes, highlighting those correlated with the SIPS ssGSEA scores, associated with Kdm4a expression, and having elevated peaks located in the promoter regions. L. Heatmap displaying the differential expression of the top 10 genes with the most significant peak differences among the 66 intersecting genes in H_2_O_2_‐treated fibroblasts following Kdm4a knockdown. M. Visualization of H3K9me3 ChIP‐seq tag profiles was conducted using screenshots from the Integrative Genomics Viewer (IGV) to display representative peaks. Selected genes were analyzed to evaluate changes in H3K9me3 levels at their respective loci. N. ChIP‐qPCR was conducted to evaluate the enrichment of H3K9me3 in different promoter regions of Trim44 in fibroblasts after silencing. (*n* = 3 /group) Data are expressed as the means SD for each group. D and N was analyzed by two‐tailed unpaired t‐test. ^*^ *p* < 0.05, ^**^ *p* < 0.01, ^***^ *p* < 0.001, ^****^ *p* < 0.0001.

To explore the mechanistic impact of Kdm4a on the premature senescence of fibroblasts and identify its specific downstream targets, we conducted transcriptome sequencing via RNA‐seq analysis to investigate alterations in gene expression in Kdm4a knockdown fibroblasts. Volcano plot analyses revealed the significant differential expression of 3846 mRNAs, of which 1765 genes were upregulated and 2081 genes were downregulated in fibroblasts transfected with si‐Kdm4a (**Figure** **6**A). The GO analysis indicated that the DEGs were mainly involved in processes related to chromatin remodeling, extracellular matrix remodeling, and autophagy (Figure 6B). Additionally, the KEGG analysis revealed the significant enrichment of gene sets related to cellular senescence, autophagy, and PI3K‐AKT signaling among the DEGs (Figure 6C). Previous studies have reported that Kdm4a is a histone demethylase that is mainly responsible for mediating H3K9me3 demethylation, which is a transcriptional repressor mark.^[^ ^29^, ^30^ ^]^ Taken together, these data collectively suggest that Kdm4a may modulate cellular senescence through the regulation of histone methylation. Our experiments verified that Kdm4a knockdown increased the level of H3K9me3 (Figure 6D). Therefore, to ascertain whether Kdm4a regulates the expression of pivotal genes through H3K9me3, we performed ChIP‐seq using DNA fragments captured by the H3K9me3 antibody to determine the differential H3K9me3 enrichment between si‐NC‐ and si‐Kdm4a‐transfected fibroblasts (Figure 6E). As expected, Kdm4a depletion significantly increased the accumulation of H3K9me3 in the peaks located within the promoter regions (±2 kb from the nearest transcription start sites (TSSs) (Figure 6F,G). The bar plot illustrating the count of differentially enriched peaks and their annotated genes in H_2_O_2_‐treated fibroblasts following Kdm4a knockdown (Figure 6H). We further analyzed these DEGs and found that Kdm4a knockdown could positively regulate the cell cycle phase transition and negatively regulate SIPS and extracellular matrix organization (Figure S10A–C, Supporting Information). We combined transcriptome sequencing with ChIP‐Seq data to further investigate direct downstream targets and identified 831 genes that might be directly regulated by Kdm4a (Figure 6I). We further found that 517 genes were positively associated with SIPS and that 540 genes were positively associated with Kdm4a regulation (Figure S10D,E, Supporting Information). Additionally, the GO analysis of the overlapping genes correlated with the ssGSEA scores of SIPS revealed the significant enrichment of extracellular matrix remodeling and autophagy (Figure 6J). We explored the mechanism by which Kdm4a mediates cellular senescence, by conducting a comprehensive gene screen and observed several downstream genes involved in cellular senescence (Figure 6K). Through a bioinformatics analysis, we prioritized the top ten genes with a 2 fold change and *p* < 0.05 (Figure 6L). Subsequent RT‒PCR, ChIP‒qPCR and western blot assays confirmed that among the regulatory changes mediated by Kdm4a, Trim44 exhibited the most significant changes (Figure S10F–H, Supporting Information). Through programmed screening, we identified Trim44 as a promising candidate gene affected by Kdm4a‐mediated H3K9me3 modifications for further investigation. Previous studies have reported that increased Trim44 levels are associated with the progression of cardiac fibrosis after MI.^[^ ^39^ ^]^ Furthermore, the results showed that silencing endogenous Kdm4a increased the peak of H3K9me3 around the TSS (Figure 6M). To further confirm this result, we knocked down Kdm4a in fibroblasts and found that the enrichment of H3K9me3 was increased at the −1.4– −1.2 kb, −1.0– −0.8 kb, −0.6– −0.4 kb and −0.4– −0.2 kb promoter regions of Trim44 (Figure 6N). Taken together, these findings indicate that the decrease of Kdm4a inhibits Trim44 expression through the accumulation of H3K9me3, identifying Trim44 as a downstream target regulated by Kdm4a.

### 2.7 Knockdown of Kdm4a Attenuates Premature Senescence of Fibroblasts by Increasing Trim44‐Mediated Autophagy

We first investigated the expression of Trim44 and observed that Trim44 levels were significantly increased after MI or exposure to H_2_O_2_. However, the expression of Trim44 was significantly reduced when Kdm4a was knocked down (Figure S11A–D, Supporting Information). To investigate the role of Trim44, we suppressed Trim44 expression in cardiac fibroblasts via siRNA and then detected the Trim44 protein and mRNA levels (Figure S11E,F, Supporting Information). Western blot assays and β‐galactosidase staining revealed that Trim44 knockdown also significantly inhibited H_2_O_2_‐induced senescence‐related protein expression and β‐galactosidase activity in cardiac fibroblasts (Figure S11G,H, Supporting Information). These results indicate that Trim44 knockdown can effectively prevent fibroblasts from entering senescence. We therefore investigated whether premature senescent fibroblasts with suppressed Trim44 expression could affect cocultured normal fibroblasts. As expected, treatment with si‐Trim44 significantly suppressed the fibroblast‐to‐myofibroblast transition and ECM generation (Figure S11I–K, Supporting Information). Overall, our results indicate that Trim44 is the downstream of Kdm4a, participating in the premature senescence of fibroblasts.

**Figure 7:**
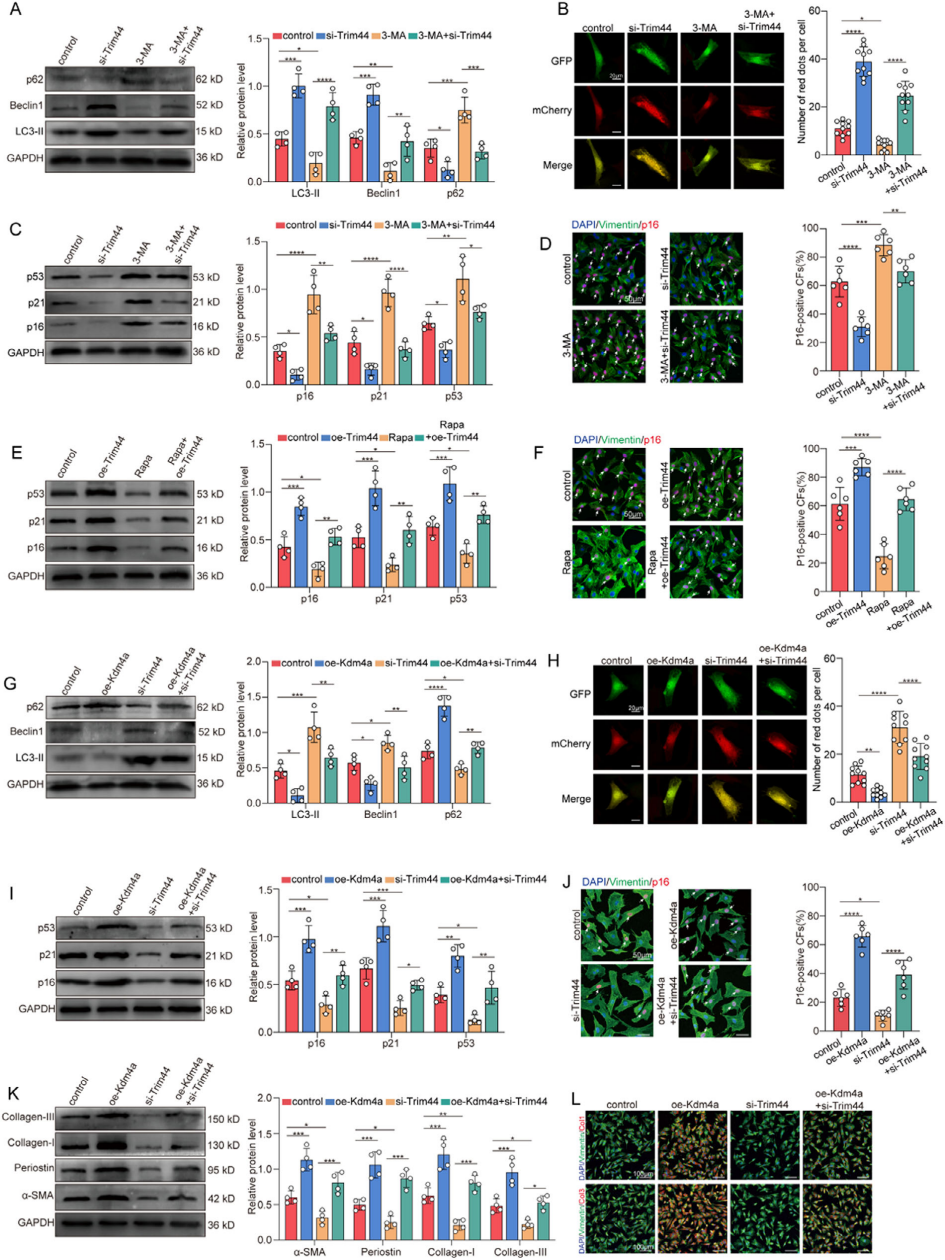
Kdm4a knockdown attenuates fibroblast senescence by regulating Trim44‐mediated autophagy A. Western blot analysis and quantification of the expression of LC3B‐II, Beclin1, and p62 protein expression in H_2_O_2_‐treated fibroblasts after si‐Trim44 and inhibition of autophagy with 3‐MA. GAPDH was used as a loading control. (*n* = 4/group) B. H_2_O_2_‐treated fibroblasts were incubated with si‐Trim44 and 3‐MA and then transfected with mCherry‐GFP‐LC3 adenovirus. LC3 spots were captured by confocal microscopy. (scale bars = 20 µm) C. Western blot analysis and quantification of the expression of p16, p21, and p53 protein levels in H_2_O_2_‐treated fibroblasts after si‐Trim44 and inhibition of autophagy with 3‐MA. GAPDH was used as a loading control. (*n* = 4/group) D. Immunofluorescence staining and quantification of p16‐positive fibroblasts to show the effects of Trim44 knockdown on the regulatory role of 3‐MA in fibroblast senescence. (scale bars = 50 µm) E. Western blot analysis and quantification of the expression of p16, p21, and p53 protein levels in H_2_O_2_‐treated fibroblasts after OE‐Trim44 and induction of autophagy with rapamycin. GAPDH was used as a loading control. (*n* = 4/group) F. Immunofluorescence staining and quantification of p16‐positive fibroblasts to show the effects of Trim44 overexpression on the regulatory role of rapamycin in fibroblast senescence. (scale bars = 50 µm) G. Western blot analysis and quantification of LC3B‐II, Beclin1, and p62 protein levels in fibroblasts to evaluate the effects of Trim44 knockdown on the regulatory role of Kdm4a in fibroblast autophagy. GAPDH was used as a loading control. (*n* = 4/group) H. Fibroblasts were transfected with adenovirus harboring mCherry‐GFP‐LC3, and then representative images of fluorescent LC3 spots were captured to evaluate the effects of Trim44 knockdown on the regulatory role of Kdm4a in fibroblast autophagy. (scale bars = 20 µm) I. Western blot analysis and quantification of p16, p21, and p53 protein levels in fibroblasts to evaluate the effects of Trim44 knockdown on the regulatory role of Kdm4a in fibroblast senescence. GAPDH was used as a loading control. (*n* = 4/group) J. Immunofluorescence staining and quantification of p16‐positive fibroblasts to show the effects of Trim44 knockdown on the regulatory role of Kdm4a in fibroblast senescence. (scale bars = 50 µm) K. Western blot analysis and quantification of the expression of fibrotic markers α‐SMA, periostin, collagen I, and collagen III expression in fibroblasts cocultured with fibroblasts from OE‐NC + si‐NC, OE‐Kdm4a+ si‐NC, OE‐NC + si‐Trim44 or OE‐Kdm4a+ si‐Trim44. GAPDH was used as a loading control. (*n* = 4/group) L. Representative images of collagen I and collagen III immunostaining in fibroblasts cocultured with fibroblasts from OE‐NC + si‐NC, OE‐Kdm4a+ si‐NC, OE‐NC + si‐Trim44 or OE‐Trim44+ si‐Trim44. (scale bars = 100 µm) Data are expressed as the means SD for each group. A‐L were analyzed by one‐way ANOVA followed by Tukey's test. ^*^ *p* < 0.05, ^**^ *p* < 0.01, ^***^ *p* < 0.001, ^****^ *p* < 0.0001.

We next explored the underlying mechanism by which Trim44 modulates premature senescence in fibroblasts. Previous studies have demonstrated that autophagy is a crucial mechanism associated with cellular senescence.^[^ ^40^ ^]^ When premature senescent cells are not effectively removed from tissues by autophagy, their accumulation can lead to tissue dysfunction and fibrosis.^[^ ^11^ ^]^ As reported previously, Trim44 exerts regulatory effect on the induction of autophagy.^[^ ^41^ ^]^ Based on this, we hypothesized that Trim44 regulates cellular senescence by modulating autophagy. Through a biological analysis, we obtained 149 genes related to Trim44 and deubiquitinase substrates in the UbiBrowser 2.0 database (Figure S12A,B, Supporting Information). GO and KEGG analysis revealed that these genes were significantly enriched in the set of genes associated with cellular senescence and autophagy (Figure 12C,D, Supporting Information). Therefore, we investigated whether Trim44 regulates cellular senescence by modulating autophagy in cardiac fibroblasts. Western blot analysis revealed that the 3‐MA‐mediated decrease of Beclin‐1 and LC3B‐II levels, together with the increase of p62 levels, was markedly enhanced by si‐Trim44 (**Figure** **7**A). Confocal microscopy assays further confirmed that si‐Trim44 significantly increased the number of yellow puncta in fibroblasts, indicating increased autophagosome accumulation caused by Trim44 knockdown (Figure 7B). Furthermore, when autophagy was inhibited by 3‐MA, the downregulation of senescence‐associated proteins induced by si‐Trim44 was enhanced (Figure 7C,D). Conversely, Trim44 overexpression counteracted rapamycin‐mediated downregulation of senescence‐associated proteins (Figure 7E,F). These results suggest that Trim44 modulates the premature senescence through autophagy in cardiac fibroblasts. We further investigated whether Trim44 can reverse the Kdm4a‐mediated phenotype of premature fibroblast senescence and myofibroblast activation, by performing rescue experiments on Kdm4a‐overexpressing fibroblasts using si‐Trim44. Both immunofluorescence assays and western blotting revealed that Kdm4a upregulation blocked the initiation of autophagy, which was also reversed by Trim44 inhibition (Figure 7G,H). GSEA futher indicated that Kdm4a knockdown could positively regulate of autophagy (Figure S12E, Supporting Information). Both immunofluorescence assays and TEM analysis further confirmed that si‐Kdm4a significantly increased the H_2_O_2_‐induced reduction in autophagosomes (Figure S12F,G, Supporting Information). Western blotting assay futher support this finding (Figure S12H,I, Supporting Information). Furthermore, we investigated whether the accelerated premature senescence of fibroblasts triggered by Kdm4a upregulation was reversed by Trim44 inhibition. This hypothesis was supported by evidence from western blot and immunofluorescence assays (Figure 7I,J). Western blotting demonstrated that Kdm4a overexpression increased the expression of α‐SMA, periostin, collagen I, and collagen III, whereas these effects were reversed when Trim44 was silenced with si‐Trim44 (Figure 7K). Subsequent immunofluorescence assays and collagen lattice contraction assays also presented similar results (Figure 7L; Figure S13A, Supporting Information). Taken together, these results indicate that Kdm4a knockdown attenuates premature senescent fibroblasts by modulating Trim44‐mediated autophagy, thereby reducing fibroblast activation and collagen accumulation.

### 2.8 Kdm4a Knockdown Prevents Fibroblast Activation by Reducing the SASP from the Premature Senescent Fibroblasts

Recent studies have revealed that SASP secreted by senescent cells includes multiple factors that promote the progression of fibrosis.^[^ ^20^ ^]^ We further investigated the key SASP factors that contribute to fibroblast activation regulated by Kdm4a. A ligand‒receptor interaction‐based strategy was used to explore the communication between stress‐induced premature senescent fibroblasts and normal fibroblasts. We then combined the secretory patterns of fibroblasts with genes positively correlated with Kdm4a from transcriptome sequencing and identified 40 genes that could be key SASP factors regulated by Kdm4a (Figure S14A, Supporting Information). We selected the top 10 ligands with the highest Pearson correlation coefficients, among which TGF exhibited the strongest correlation (Figure 14B, Supporting Information). Based on the receptor‒ligand model, we predicted potential interacting receptors and genes that might act downstream of these ten genes (Figure S14C,D, Supporting Information). Heatmap analyses revealed that the expression of the top 10 ligands was decreased in H_2_O_2_‐treated fibroblasts transfected with si‐Kdm4a (Figure S14E, Supporting Information). GO and KEGG analysis revealed that the top 10 ligands were closely related to the regulation of the fibrotic pathway (Figure S14F,G, Supporting Information). GSEA also suggested that the SASP plays an important role in promoting fibroblast activation (Figure S14H, Supporting Information). Moreover, previous studies have shown that TGF‐β1 is an important factor in promoting myocardial fibrosis.^[^ ^42^ ^]^ We therefore focused our investigation on TGF‐β1 to elucidate the mechanism by which H_2_O_2_‐induced senescent fibroblasts facilitate the activation of surrounding normal fibroblasts.

**Figure 8:**
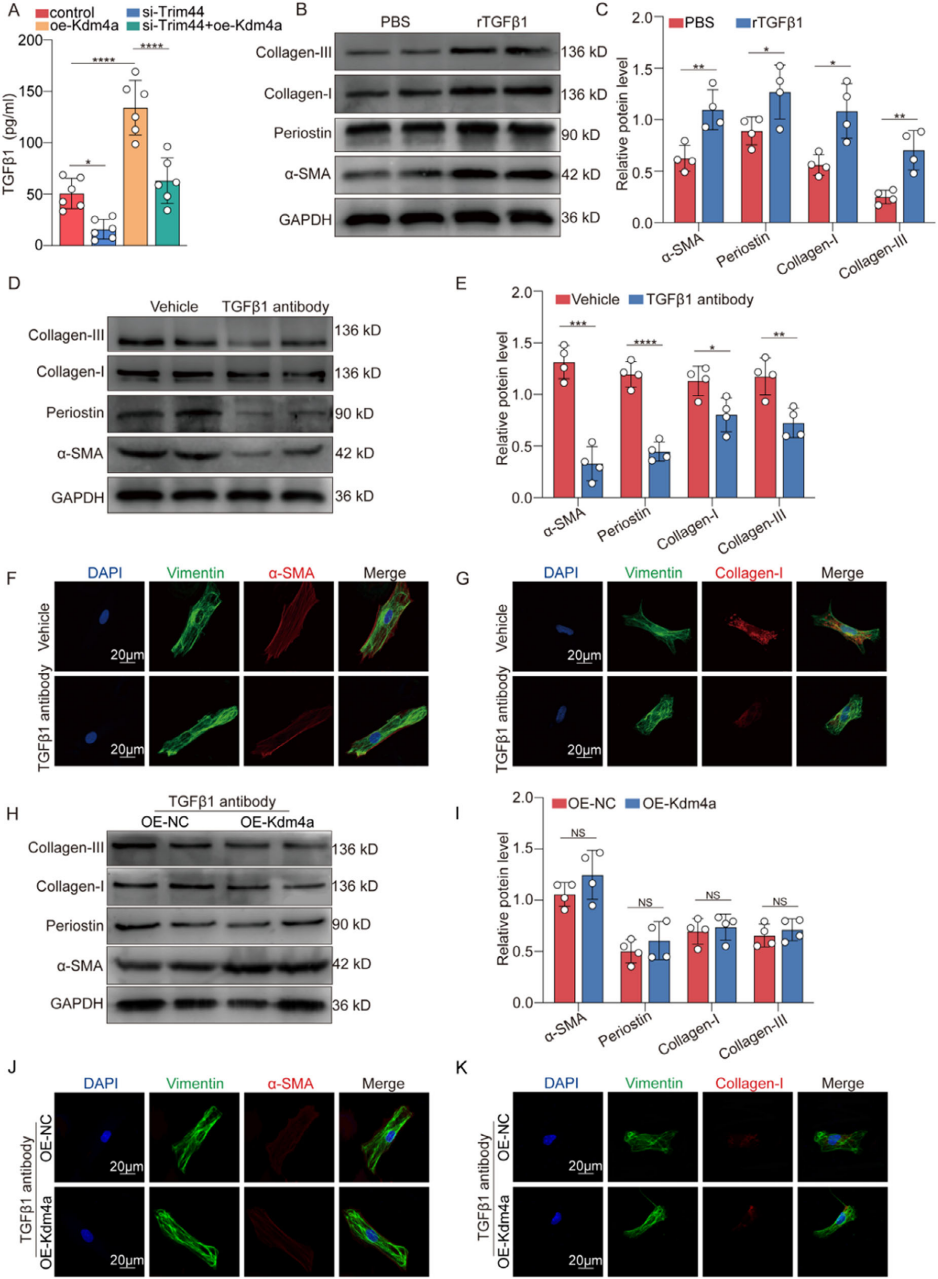
Kdm4a knockdown prevents fibroblast activation by inhibiting SASP secretion A. TGF‐β1 was detected by enzyme‐linked immunosorbent assay (ELISA). ELISAs were performed on conditioned medium collected from fibroblasts incubated in OE‐NC + si‐NC, OE‐Kdm4a+ si‐NC, OE‐NC + si‐Trim44 or OE‐Kdm4a+ si‐Trim44. (*n* = 6/group) B‐C. Western blot analysis and quantification of α‐SMA, periostin, collagen I and collagen III protein levels in fibroblasts with or without exogenous rTGF‐β1 treatment. (*n* = 4/group) D‐E. Western blot analysis and quantification of α‐SMA, periostin, collagen I and collagen III protein levels in fibroblasts cocultured with H_2_O_2_‐induced fibroblasts with vehicle or TGF‐β1 neutralising antibody. (*n* = 4/group) F‐G. Representative images of α‐SMA and collagen I immunostaining in fibroblasts which were cocultured with H_2_O_2_‐induced fibroblasts incubated with vehicle or TGF‐β1 neutralising antibody. (scale bars = 20 µm) H‐I. Western blot analysis and quantification of α‐SMA, periostin, collagen I and collagen III protein levels in fibroblasts cocultured with H_2_O_2_‐induced fibroblasts with TGF‐β1 neutralising antibody+OE‐NC or TGF‐β1 neutralising antibody+OE‐Kdm4a. (*n* = 4/group) J‐K. Representative images of α‐SMA and collagen I immunostaining in fibroblasts cocultured with H_2_O_2_‐induced fibroblasts with TGF‐β1 neutralising antibody+OE‐NC or TGF‐β1 neutralising antibody+OE‐Kdm4a. (scale bars = 20 µm) Data are expressed as the means SD for each group. A was analyzed by one‐way ANOVA followed by Tukey's test. C, E and I were analyzed by two‐tailed unpaired t‐test. ^*^ *p* < 0.05, ^**^ *p* < 0.01, ^***^ *p* < 0.001, ^****^ *p* < 0.0001.

We found that TGF‐β1 levels were significantly increased in the supernatant of fibroblasts when Kdm4a was overexpressed, which was strongly attenuated by si‐Trim44 (**Figure** **8**A). We also selected a panel of classic SASP factors and assessed the alterations in supernatant expression following Trim44 knockdown and Kdm4a overexpression (Figure S15A, Supporting Information). Furthermore, our western blotting and immunofluorescence staining results revealed that the addition of the recombinant TGF‐β1 (rTGF‐β1) protein effectively promoted normal fibroblast activation and collagen deposition (Figure 8B,C; Figure S15B,C, Supporting Information). To prevent autocrine stimulation, we neutralized secreted TGF‐β1 in the culture medium via antibody‐mediated neutralization, thereby inhibiting its binding to cognate receptors. However, when the fibroblasts were incubated with the anti‐TGF‐β1 antibody, the opposite effect was observed (Figure 8D–G). We further investigated whether Kdm4a deficiency could inhibit fibroblast activation by blocking TGF‐β1 secretion from premature senescent fibroblasts. The results of western blotting and immunofluorescence staining revealed that the fibroblast activation and collagen deposition induced by Kdm4a overexpression disappeared when secreted TGF‐β1 was blocked (Figure 8H–K). Collectively, our results indicate that TGF‐β1 is a critical component of the SASP secreted by premature senescent fibroblasts, which exerts influence on the stimulation of fibroblast activation and collagen deposition.

We further explored the mechanism by which Kdm4a mediates SASP secretion. Recent studies have demonstrated that senescent cells primarily regulate the SASP through transcriptional regulators, notably the NF‐κB signaling pathway.^[^ ^43^ ^]^ We next explored whether Kdm4a regulates SASP secretion by activating the NF‐κB pathway. Flow cytometry demonstrated that Trim44 inhibition effectively reduced the accumulation of Kdm4a‐induced senescent fibroblasts (Figure S15D, Supporting Information). Subsequently, western blot analysis revealed that Kdm4a knockdoen significantly decreased the phosphorylation of NF‐κB in H_2_O_2_‐induced fibroblasts, suggesting impaired NF‐κB activation (Figure S15E, Supporting Information). These findings suggest that Kdm4a knockdown can attenuate SASP secretion by reducing the number of senescent fibroblasts and suppressing NF‐κB pathway activation.

### 2.9 Pharmacological Inhibition of Kdm4a Ameliorates Cardiac Fibrosis and Promotes Cardiac Repair Post‐MI in Adult Mice

Given the observed beneficial effects of Kdm4a deletion on cardiac fibrosis and post‐injury repair, we investigated the potential clinical value of targeting Kdm4a via pharmacological inhibition. To this end, we used a small molecule inhibitor of Kdm4a to assess its ability to inhibit Kdm4a function. In subsequent studies, we selected ML324 as a Kdm4a inhibitor by analyzing the protein structure of Kdm4a (Figure S16A, Supporting Information). The molecular docking analysis showed that ML324 could bind to the active site of the Kdm4a protein, forming hydrophobic interactions with the residues Ala69, Ile71, Tyr132, and Trp208. Additionally, ML324 formed a hydrogen bond with Tyr132 and both a hydrogen bond and a salt bridge with Asp135 (Figure S16B, Supporting Information). These results indicate that ML324 has a stable binding interaction with Kdm4a, with a binding affinity of −5.106. We further investigated the effects of ML324 treatment on myocardial fibrosis and cardiac function after MI. Cardiac function was significantly improved in MI mice injected with ML324 (Figure S16C,D, Supporting Information). Moreover, treatment with ML324 significantly increased the survival rate after MI compared to the control group and did not increase the risk of ventricular rupture in the first week after MI (Figure S16E,F, Supporting Information). In addition, ML324 treatment significantly reduced the size of the cardiac fibrotic area and total collagen deposition (Figures S16G,H and S17A, Supporting Information). Furthermore, ML324‐treated mice presented reduced collagen accumulation in the border zone, as assessed by HE staining, picrosirius red staining, and Masson's trichrome staining (Figure S17B–D, Supporting Information). Collectively, these results suggest that treatment with ML324 may have a protective effect against the development of cardiac fibrosis and may promote the repair of adult cardiac function.

## 3 Discussion

In this study, we creatively verified that premature senescent fibroblasts could only promote interstitial fibrosis at 7 days post‐MI without affecting replacement fibrosis during the acute phase, the inhibition of which could strongly restrict advanced cardiac remodeling and failure but not cause cardiac rupture in the early stage after MI. Excitingly, we further identified Kdm4a as a crucial promoter of premature senescence in fibroblasts, which induces cardiac interstitial fibrosis. Silencing Kdm4a could promote autophagy in premature senescent fibroblasts by increasing the level of H 3K9m3 modification of the Trim44 promoter, thereby suppressing the profibrotic effect of SASP factors secreted by premature senescent fibroblasts in the injured myocardium. Our findings indicate that targeting Kdm4a‐induced premature senescent fibroblasts might be a clinically valuable therapy that restricts excessive cardiac fibrosis and does not promote ventricular rupture after MI.

We innovatively reported that premature senescent fibroblasts could only accelerate interstitial fibrosis 7 days post‐MI but did not affect the replacement fibrosis within 7 days, which is clinically relevant for effectively preventing prolonged cardiac remodeling and dysfunction in the advanced stage and avoiding early ventricular rupture after MI. Previous studies have reported that antifibrotic factors, such as plasminogen activators and macrophage‐derived VSIG4,^[^ ^44^, ^45^ ^]^ not only reduce excessive cardiac interstitial fibrosis in the advanced stage (7 days post‐MI), but also unfortunately increase the incidence of cardiac rupture in the first week after MI, which could result in undesirable consequences. In our study, we found that premature senescent fibroblasts rarely exist in the injured myocardium in the first week after MI, but sharply increase at 7 days post‐MI, which is supported by prior studies reporting that the expression level of p21, the key senescence marker, was significantly upregulated at 1‐week post‐MI.^[^ ^18^ ^]^ The results of functional experiments further verified that inhibiting premature senescent fibroblasts does not reduce replacement fibrosis in the infarct zone or increase the incidence of ventricular rupture in vivo, indicating that targeting the premature senescence of fibroblasts could effectively prevent cardiac rupture in the early stage after MI. More importantly, we found that premature senescent fibroblasts play a crucial role in interstitial fibrosis in the advanced stage after MI. First, premature senescent fibroblasts persistently exist in the injured myocardium more than 4 weeks post‐MI, similar to previous reports that premature senescent cells steadily accumulate in bleomycin‐induced pulmonary fibrosis.^[^ ^11^, ^25^ ^]^ Second, the functional experiments demonstrated that inhibiting premature senescent fibroblasts could depress the transition of myofibroblasts and the deposition of ECM in vitro and visibly reduce interstitial fibrosis and ECM deposition in the border zone in vivo. Finally, the levels of SASP factors secreted by premature senescent fibroblasts, including fibrogenic growth factor (TGF‐β), cytokines (including IL‐1β and IL‐6) and MMPs, are dramatically increased, facilitating the transformation of myofibroblasts and the deposition of ECM. Consistent with our results, SASP factors, which can alter the behavior of neighboring cells and the tissue microenvironment, have also been found to promote the fibrotic process in the lung, liver and kidney.^[^ ^20^, ^46^ ^]^ Our results indicate that reducing the premature senescence of fibroblasts might be a clinically valuable strategy to solely reduce excessive interstitial fibrosis and restrict cardiac remodeling and dysfunction after MI, without resulting in ventricular rupture.

Another important finding in our study is that Kdm4a is a critical inducer of premature senescent fibroblasts, leading to cardiac interstitial fibrosis. In our study, we demonstrated that silencing Kdm4a strongly inhibited the premature senescence of fibroblasts and interstitial fibrosis in the border zone, and then significantly improved the LVEF from 21% to 42% in vivo, which is close to or even exceeding reported in previous studies.^[^ ^20^ ^]^ The antifibrotic effect of Kdm4a silencing revealed in this study could be partially supported by previous studies on the function of Kdm4a in multiple diseases, including liver fibrosis,^[^ ^31^ ^]^ hypercholesterolemia‐induced heart failure^[^ ^47^ ^]^ and intervertebral disc degeneration.^[^ ^34^ ^]^ In contrast to senolytic agents, which have been reported to promote senescent cell death to decrease the senescent cell burden in tissues,^[^ ^48^ ^]^ the histone demethylase Kdm4a, a key epigenetic regulator, can accurately and directly regulate the premature senescence of fibroblasts, which is partly supported by a previous study showing that Kdm4a can epigenetically induce DNA damage,^[^ ^33^, ^49^ ^]^ an important initiator of cellular senescence in aging. Moreover, we found that Kdm4a increases the expression of SASP factors from premature senescent fibroblasts, then accelerating interstitial fibrosis through various classic fibrosis‐associated pathways, including ECM‒receptor interactions, the TGF‒β signaling pathway, and the TNF signaling pathway. In addition, we confirmed that ML324,^[^ ^50^ ^]^ a small‐molecule inhibitor of Kdm4a, suppresses interstitial fibrosis in the border zone after MI, indicating that ML324 shows promise for treating cardiac fibrosis in clinical practice. Thus, inhibiting Kdm4a might be a potential avenue to restrict excessive cardiac fibrosis.

We further revealed that Kdm4a induces the premature senescence of fibroblasts by depressing cell autophagy through the H3K9me3 modification of Trim44, resulting in cardiac interstitial fibrosis. Previous studies have demonstrated that Bcl2, PI3K/AKT, and mTORC1 are involved in regulating cellular senescence by promoting senescent cell death or intervening in the SASP modulation.^[^ ^20^, ^48^ ^]^ In this study, we novelly uncovered that autophagy, which is mediated by Kdm4a, can also depress the premature senescence of fibroblasts by directly clearing damaged proteins and organelles. First, we found that accompanied by the accumulation of premature senescent fibroblasts, autophagic activity is substantially decreased in vitro and in vivo. Moreover, the initiation of autophagy could significantly reduce the premature senescence induced by Kdm4a in fibroblasts, similar to recent findings that metformin suppresses vascular smooth muscle cell senescence by promoting autophagic flux,^[^ ^51^ ^]^ and supported by the fact that autophagy could effectively maintain cellular quality, thereby inhibiting cellular senescence.^[^ ^11^ ^]^ To explore the mechanism by which Kdm4a regulates autophagy in premature senescent fibroblasts, we applied ChIP‐seq and RNA‐seq to screen the downstream of Kdm4a and found that the H3K9me3 profile of Trim44, an autophagy regulatory factor, was dramatically increased. Immunoprecipitation experiments further demonstrated that Kdm4a binds to the Trim44 promoter at the H3K9me3 position, which is consistent with a previous report that Kdm4a can mediate H3K9me3 accumulation at the promoter region of HIF1α, regulating cell autophagy under hypoxic conditions.^[^ ^52^, ^53^ ^]^ Additional experiments indicated that the effect of Kdm4a on premature senescent fibroblasts could be reversed by Trim44 silencing, which revealed that Kdm4a promotes the premature senescence of fibroblasts through Trim44‐mediated autophagy, leading to cardiac interstitial fibrosis, and uncovering a novel molecular pathway of Kdm4a in the fibrotic process. In addition, we found that Trim44 deletion promoted autophagy in premature senescent fibroblasts, reducing the myofibroblast transition and ECM deposition. Similar to our results, previous studies reported that cardiac‐specific Trim44 knockout attenuates isoproterenol‐induced cardiac remodeling by inhibiting the AKT/mTOR pathway.^[^ ^39^ ^]^ Collectively, these findings illustrate the important role of Trim44 in Kdm4a‐mediated premature senescence and cardiac interstitial fibrosis.

There are several limitations to the current study. First, we found that in addition to being located mainly in fibroblasts, Kdm4a is also expressed in other cells, such as cardiomyocytes. The function of Kdm4a in other types of cells after MI should be investigated in further studies. In addition, Kdm4a was silenced by an adeno‐associated virus carrying the fibroblast‐specific Postn promoter, which is widely used to deliver target genes in fibroblasts, but the use of fibroblast‐specific knockout and overexpression transgenic mice may be helpful in determining the role of Kdm4a in cardiac interstitial fibrosis. In conclusion, premature senescent fibroblasts play a pivotal role in promoting cardiac interstitial fibrosis in the advanced stage after MI without affecting replacement fibrosis, which can be inhibited by Kdm4a deletion through Trim44‐mediated autophagy of premature senescent fibroblasts. These findings may provide a valuable strategy to reduce cardiac interstitial fibrosis and remodeling after MI by promoting the autophagy of premature senescent fibroblasts.

## 4 Experimental Section

### Animal Models

C57BL/6 mice were procured from the Guangdong Medical Laboratory Animal Center. All experiments were approved by the Animal Research Committee of Nanfang Hospital, Southern Medical University (Approval no. NFYY‐2020‐1137) and adhered to the stipulations outlined in Directive 2010/63/EU of the European Parliament concerning the protection of animals utilized for scientific research. All the experiments were conducted on age‐matched mice. The mice were housed under standard environmental conditions with a 12 h light/dark circadian cycle and were granted unrestricted access to both food and water. MI of adult mouse hearts was performed as described in previous studies.^[^ ^54^ ^]^ The mice (8–10 weeks old, male) were anesthetized via isoflurane inhalation, with a 5% concentration used for induction, followed by a 2% concentration for maintenance. During the procedure, the mice were connected to an animal ventilator to maintain the airway. A left‐sided thoracotomy was performed at the fourth intercostal space, followed by permanent ligation of the left coronary artery (LAD) via a 7–0 nylon suture ligated 1 mm below the left atrium. A similar procedure was performed on sham‐operated animals but without the occlusion of the LAD.

### Cas9 Knockin Transgenic Mouse Model

The Cre‐dependent Cas9 knock‐in mouse model (R26‐CAG‐Cas9/+) was purchased from GemPharmatech (Jiangsu, China). The Rosa26 locus in mice has been shown to stably express exogenous genes integrated at this site and has been widely used for the generation of various knock‐in and Gene Trap mouse models. The targeting vector contained a ubiquitously expressed CAG promoter, a loxP‐flanked PGK‐Neo‐polyA sequence, and a Cas9 gene inserted into intron 1 of the Rosa26 locus. After confirming the correct insertion, the constructs were linearised and electroporated into JM8A3 embryonic stem cells. Chimeric mice were obtained by injecting the correct targeting colonies into blastocysts. The high proportion of chimeric male mice was then crossed with female C57BL/6J mice to produce heterozygous Cre‐dependent Cas9 mice (Rosa26‐LSL‐Cas9‐tdTomato/+). These mice were then intercrossed to obtain homozygous Rosa26‐LSL‐Cas9‐tdTomato mice. Upon the presence of Cre recombinase, the STOP elements are excised, enabling the expression of the transgene or viral vector‐delivered sgRNAs, which facilitates tissue‐ and organ‐specific gene knockout.

### Echocardiogram

Transthoracic echocardiographic assessments were conducted at specified intervals after surgery using the Vevo 770 imaging system (VisualSonics, Toronto, Canada). The mice were anesthetized with inhaled isoflurane at concentrations ranging from 0.4% to 1.5%. Throughout the procedure, the heart rate was consistently maintained between 450 and 550 beats per minute. 2D guided M‐mode measurements were conducted in the short parasternal long‐axis plane of the papillary muscle. At least three measurements were performed and then averaged. Measurements were consistently conducted by the same investigator, who was blinded to the experimental groups.

### Fibroblast Isolation and Culture

Primary cardiac fibroblasts (CFs) were isolated from 6‐week‐old C57BL/6J mice and performed according to previous studies.^[^ ^55^ ^]^ Anesthesia was administered to the mice via 5% isoflurane, after which they were euthanized via cervical dislocation. The cleaned ventricular tissue was dissected into smaller fragments. The mixture was subsequently digested with a mixed digestion solution of 0.08% trypsin (Beyotime Biotechnology, Shanghai, China) and 0.06% type II collagenase (Bopei Biotech Co., Ltd., Guangzhou, China). The digestion was facilitated for 10 min at 37 °C with intermittent agitation. Next, the suspension of CFs was centrifuged, resuspended, and plated. After incubation at 37 °C for 2 h, the CFs adhered to the culture plates, after which nonadherent cells in the supernatant were subsequently discarded. CFs were cultured for ≈3‒4 days until they reached confluence, after which they were passaged. The identity of the CFs was ascertained based on vimentin expression. Experiments were conducted using third‐ or fourth‐passage CFs.

### Isolation of Adult Mouse CMs

Primary adult CMs were isolated from the hearts of adult mice treated with Saline and AAV‐Posten‐shKdm4a at 14 days after transduction using an angendorff perfusion system. Mice were anesthetized with isoflurane (5% for induction, 2% for maintenance) and hearts were excised via thoracotomy. After isolating the aorta, the hearts were connected to a Langendorff perfusion system and perfused with calcium‐free buffer (composition in mM: NaCl 113; KCl 4.7; KH2PO4 0.6; Na2HPO4 0.6; MgSO4 1.2; Na‐HEPES 10; NaHCO3 12; KHCO3 10; phenol red 0.032; taurine 30; BDM 10; glucose 5.5) until residual blood was completely removed. Subsequently, the hearts were digested at 37 °C using 50 mL of perfusion buffer containing 15 000 U of type II collagenase (Roche) and 50 µM CaCl2. Digestion continued until the heart became noticeably pale and flaccid. The tissue was then gently minced using forceps and triturated with a Pasteur pipette to isolate individual CMs. Cell suspension was centrifuged (1000 rpm, 5 min) and resuspended in laminin‐coated plates for 2 h CM selection, followed by medium replacement with DMEM/F12 supplemented with 10% FBS to remove non‐cardiomyocyte.

### Isolation and Culture of Adult Mouse Cardiac ECs

Primary cardiac endothelial cells (ECs) were isolated from adult mice and cultured as our previous study. Mice were anesthetized with isoflurane (5% for induction, 2% for maintenance), and hearts were harvested via thoracotomy. The heart tissues were carefully rainsed with cold PBS to remove residual blood. The heart tissues were enzymatically digested with 0.1% trypsin at 37 °C for 10 min and then incubated with 0.1% type II collagenase at 37 °C after centrifugation to obtain single‐cell suspensions. The resulting cell suspension was filtered through a 70‐µm cell strainer to remove debris, then centrifuged and washed with PBS. And then the cell suspension was incubated with magnetic beads conjugated with an anti‐CD31 antibody. The CD31‐positive ECs were separated using a magnetic column, washed multiple times, and prepared for further applications. The identity and purity of ECs were confirmed by fluorescence staining with an anti‐CD31 antibody. Isolated ECs were cultured in DMEM/F12 medium supplemented with 10% FBS and incubated at 37 °C with 5% CO_2_.

### Human Cardiac Fibroblast Culture

Human primary cardiac fibroblasts (CP‐H078) were obtained from Pricella Biotechnology (Wuhan, China). These fibroblasts were isolated following the same protocol as previously used in our studies. The isolation process involved a combination of collagenase‐trypsin digestion and differential adhesion. After enzymatic digestion, the cell suspension was centrifuged, resuspended, and plated onto culture dishes. The cells were incubated at 37 °C for 2 h to allow fibroblast adhesion, after which nonadherent cells in the supernatant were removed. Fibroblasts were maintained in culture for ≈3–4 days until reaching confluence, at which point they were passaged. The identity of the fibroblasts was confirmed by vimentin expression. All experiments were performed using second‐ to third‐passage fibroblasts.

### SA‐β‐Gal Staining

Cardiac muscle tissue or cells induced to senescence were stained using the SA‐β‐Gal Staining Kit (C0602, Beyotime) according to the manufacturer's protocol. Appropriate tissues or cells were fixed with the fixative provided for 15 min according to the relevant instructions and then washed 3 times with PBS for 5 min. The tissues or cells were washed, followed by staining with the SA‐β‐gal solution at 37 °C for 24 h in an environment without CO_2_. The tissues or cells were subsequently washed with PBS and photographed.

### RNA Isolation and Real‐Time qPCR

Total RNA was extracted from fibroblasts or cardiac muscle tissue using TRIzol reagent (Thermo) according to the manufacturer's protocol. Total RNA was then reverse transcribed to cDNA using the QuantiTect Reverse Transcription Kit (Qiagen Co., Germany). Quantitative real‐time PCR was conducted using SYBR Premix Ex Taq II (TaKaRa) on a Light Cycler 480 II instrument (Roche Diagnostics, Basel, Switzerland) real‐time qPCR system. Relative gene expression was determined using the 2‐∆CT method, with glycerol 3‐phosphate dehydrogenase (GAPDH) serving as an endogenous control. The PCR primers were designed and synthesized by Tsingke (Guangzhou, China). The primer sequences are listed in Table S2 (Supporting Information).

### Immunofluorescence Staining

Myocardial tissue sections or cells were washed with PBS and subsequently fixed with 4% paraformaldehyde for 30 min. Then, the cells or tissue sections were permeabilized with 0.1% Triton X‐100 for 10 min and washed with PBS. The sections were then treated with 2% bovine serum albumin (BSA) for 1 h at room temperature to block nonspecific binding. Following this, the samples were incubated with the indicated antibodies (1:100 dilution) overnight at 4 °C and then treated with AF‐488‐ or AF‐594‐labeled anti‐mouse or anti‐rabbit secondary antibodies (#A‐11001, Invitrogen) for 1 h at room temperature. Finally, the cell nuclei were counterstained with DAPI. The results were measured using a confocal laser scanning microscope (Leica).

### TUNEL Assay

The apoptosis rate of CMs was assessed using the TUNEL assay according to the manufacturer's instructions. Briefly, tissue sections were rinsed in PBS and permeabilized with 0.1% Triton X‐100 for 10 min at room temperature. After rinsing with PBS, the sections were incubated with the TUNEL reaction mixture for 1 h at 37 °C in the dark. Nuclei were counterstained with DAPI for 15 min. Fluorescence images were acquired with a Leica confocal microscope. Apoptotic cells were quantified by counting TUNEL‐positive nuclei in five randomly selected fields of view per section using ImageJ software. The percentage of apoptotic cells was calculated as the ratio of TUNEL‐positive cells to the total number of DAPI‐stained nuclei.

### Transmission Electron Microscopy (TEM)

Treated fibroblasts were fixed in 2.5% glutaraldehyde in 0.1 m phosphate buffer (pH 7.4) at 4 °C for 2 h, followed by post‐fixation in 1% osmium tetroxide (OsO₄) for 1 h at room temperature. After thorough washing with PBS, the samples were dehydrated in a graded series of ethanol (30%, 50%, 70%, 90%, and 100%) and embedded in epoxy resin. Ultrathin sections (70–90 nm) were cut using an ultramicrotome and collected on copper grids. The sections were then stained with uranyl acetate and lead citrate to enhance contrast. Imaging was performed using a transmission electron microscope (TEM) at an accelerating voltage of 80–120 kV.

### Immunohistochemical Staining

Paraffin‐embedded myocardial tissue was sectioned into 4 µm slices and dewaxed. The conditions for antigen repair were determined based on the guidelines provided by the appropriate antibodies. The paraffin sections were exposed to H_2_O_2_ in 0.3% warm deionized water for 30 min to block endogenous peroxidase activity, followed by normal nonimmune animal serum to prevent nonspecific adsorption. The target primary antibodies were chosen based on the experimental requirements and incubated overnight at 4 °C. Subsequently, the sections were incubated with horseradish peroxidase (HRP)‐conjugated secondary antibody for 1 h at room temperature the next day. The sections were developed with DAB for 10 min and counterstained with hematoxylin. Thereafter, the sections were cleaned, dehydrated, permeabilized, and finally observed under a microscope.

### Small Interfering RNA (siRNA) and AAV9 Transduction

Specific siRNA sequences were meticulously designed and synthesized by Tsingke (Guangzhou, China). The corresponding siRNA sequences are listed in Table S3 (Supporting Information). Subsequent transfections of cells with siRNAs were performed using Lipofectamine 3000 (Invitrogen) according to the manufacturer's protocol. At the point of transfection, the fibroblasts had been seeded and reached a confluence level of 70–90%. The DNA‐Lipofectamine 3000 complexes were subsequently added to the cells in a serum‐free medium. A scrambled siRNA served as the negative control (NC). siRNAs were transfected at a final concentration of 50 nm, and subsequent experiments were conducted at 48 h post transfection.

The AAV9 vector for knockout was procured from PackGene Biotech (Guangzhou, China). The mice were allocated randomly into control and experimental groups (*n* = 20 per group) using a computer‐generated sequence. During MI surgery, the AAV9 vector was delivered into the mice via in situ multipoint injections into the left ventricle. Briefly, adult mice were intracardially injected with an AAV9 vector expressing sh‐Kdm4a or an empty vector at a dose of 10 × 10^11^ viral genomic particles per mouse (≈30 µL). Two weeks after the AAV injection, the mouse hearts were isolated for real‐time PCR (qRT‒PCR) analyses to determine the success of transduction and for subsequent related experiments. The information of the virus is: AAV‐shKdm4a [ssAAV9‐periostin‐EGFP‐shRNA‐WPRE‐SV40pA] / AAV‐shNC [ssAAV9‐periostin‐EGFP‐WPRE‐SV40pA].

### Recombinant Adeno‐Associated Virus (AAV) Construction

The sgRNA (Kdm4a) sequences were cloned into the pAdeno‐associated vector (AAV‐U6‐sgRNA‐TCF21‐CRE‐T2A‐EGFP) backbone, and the adeno‐associated viral packaging was produced by HanBio Technology (Shanghai, China). The target sequence of sgKdm4a was 5′‐ATTGCCTACATTGAATCCCA‐3′. The information of the virus is: AAV‐sgRNA [AAV‐U6‐sgRNA‐TCF21‐CRE‐T2A‐EGFP] / AAV‐null [AAV‐TCF21‐CRE‐T2A‐EGFP].

### Collagen Gel Contraction Assay

Fibroblasts were processed and subsequently digested with trypsin‐disodium ethylenediaminetetraacetic acid (EDTA), and the cells were resuspended in serum‐free DMEM at a concentration of 3 × 10^6^ cells mL^−1^. Following this, 100 µL of the cell suspension was mixed with 400 µL of a collagen solution (Advanced Biomatrix, USA) according to the instructions. Five hundred microlitres of the resulting mixture were added to 24‐well cell culture plates and incubated at 37 °C for 1 h. After 1 h, 600 µL of DMEM was added to each well, and the collagen gels were released from the edges. The gel surface area was calculated using ImageJ software and presented as a normalized value relative to the initial gel size.

### Cell Counting Kit‐8 (CCK‐8) Assay

Cell viability was assessed using the CCK‐8 method. Following transfection, the cells were counted using a cell counting plate and subsequently inoculated into 96‐well plates at a density of 1000 cells per well (100 µL/well). The plates were then incubated at 37 °C with 5% CO_2_. After the addition of 10 µL of CCK‐8 solution to each well, the plates were incubated for 2 h in the incubator. After the incubation, the optical density (OD) value at 450 nm for each well was measured using a microplate reader.

### Wound Healing Assay

The migration ability of the cells was evaluated through a wound healing assay. The cells were digested and subsequently plated into six‐well plates at a density of 2.5 × 10^5^ cells/well. When the cells reached 80% confluence, they were transfected. After transfection, the cells were scratched with the tip of a sterile 200 mL micropipette and then gently rinsed twice with PBS to debride the scratched cells. The plates were then incubated at 37 °C in a 5% CO_2_ environment. Images were captured at 0, 6, 12, and 24 h post scratching. The mean migration distance between cells was computed via ImageJ software.

### Flow Cytometry Analyses

Fibroblasts were digested with trypsin, followed by centrifugation (1000 rpm × 5 min), washing (cold PBS), and collection. The collected fibroblasts were resuspended in 0.1 mL of binding buffer according to the manufacturer's instructions. The cell samples were subsequently incubated with the propidium iodide (PI) working solution at 37 °C in the dark for 30 min. Then, the samples were analyzed via flow cytometry (FACScan, BD Biosciences), and the acquired data were analyzed using FlowJo 7.6.1. Each sample was analyzed three times and at least three independent experiments were performed for statistical analysis.

### Flow Cytometry Analyses for Senescent Cells

Mouse hearts were rapidly washed with cold PBS to remove the blood and then minced into small fragments. The tissue was enzymatically digested with 0.06% type II collagenase and 0.08% trypsin at 37 °C to obtain single‐cell suspensions. The suspension was filtered through a 70 µm cell strainer to remove debris and centrifuged at 1000 rpm for 5 min. The supernatant was discarded, and the cell pellet was resuspended in a staining buffer for further analysis. For senescence detection, cells were incubated with Senescence‐associated β‐galactosidase (SA‐β‐Gal) fluorogenic substrate (5µM, AAT Bioquest, 14030) at 37 °C for 30 min in the dark. After incubation, cells were washed three times with PBS to remove excess dye and analyzed by flow cytometry (BD FACSCanto II). The fluorescence signal of a cleaved substrate, indicating SA‐β‐Gal activity, was detected using the FITC channel (530 nm). The mean fluorescence intensity (MFI) was used to quantitatively assess SA‐β‐Gal activity as a marker of cellular senescence.

For cellular senescence assessment in cultured cardiac fibroblasts, cells were harvested by trypsinization and resuspended in PBS. Senescent fibroblasts were identified by staining for SA‐β‐Gal activity using a fluorogenic substrate, as described above, and by surface marker expression. All experiments were conducted in triplicate, and unstained, single‐stained, and fluorescence‐minus‐one (FMO) controls were included to ensure specificity and accurate gating. Data were analyzed using FlowJo software.

### Western Blotting

Proteins were extracted from mouse heart tissues and CFs using RIPA buffer supplemented with the protease inhibitor phenylmethanesulfonyl fluoride (Beyotime, ST506) and phosphatase inhibitors (Beyotime, P1081). The protein content in the resulting supernatant was quantified using a BCA protein assay kit (Beyotime Biotechnology, Shanghai, China). Proteins were subsequently denatured through heating, resolved via sodium dodecyl sulfate‒polyacrylamide gel electrophoresis (SDS‒PAGE), and transferred onto polyvinylidene fluoride (PVDF) membranes. After the membranes were blocked with 5% BSA in Tris‐buffered saline with Tween 20 (TBST) for 1 h at room temperature, the membranes were incubated with primary antibodies at 4 °C overnight. The membranes were rinsed three times with 0.05% TBS‐T for 10 min each, followed by a 1 h incubation with HRP‐conjugated secondary antibodies at room temperature. The immunopositive bands were detected using enhanced chemiluminescence (Advance, No. RPN2235; GE Healthcare Life Sciences). Western blots were independently repeated in triplicate, and quantification was accomplished through ImageJ software. The intensity values were normalized to the total intensity values of β‐actin or GAPDH.

### Enzyme‐Linked Immunosorbent Assay (ELISA)

The concentrations of TGF‐β1, IL‐6, IL‐1β, TNF‐α, MMP‐2, and fibronectin in the cell supernatants were measured using ELISAs according to the manufacturer's protocol. Briefly, the supernatant was collected, added to the wells of a mouse microtiter plate, and then incubated at 37 °C for 1 h. After a washing step with the provided solution, 100 µL of mouse conjugate was added to each well and incubated at 37 °C for 1 h. The washing procedure was repeated, 200 µL of substrate solution was added, and the mixture was incubated for 30 min. Finally, 50 µL of stop solution was added to each well, and the absorbance at 450 nm was measured within 30 min using an ELISA plate reader (Spectra Max M5, Molecular Devices, California, United States). The quantification of secreted peptides was performed with at least three independent culture replicates.

### Hematoxylin and Eosin (H&E) Staining

Following euthanasia, the cardiac tissue was rinsed and then fixed with 4% paraformaldehyde for at least 24 h. These tissue samples were dehydrated with ascending ethanol concentrations and embedded in paraffin. The hearts were further sectioned longitudinally into 4 µm slices. The paraffin sections were then further subjected to H&E staining, Masson's trichrome staining, picrosirius red staining, and immunostaining.

### Masson's Trichrome Staining

Paraffin sections were deparaffinized and then subjected to modified Masson's trichrome staining (MST 8004; MST Biotechnology) according to the manufacturer's instructions. After staining, the sections were observed under a microscope, and ImageJ software (NIH, Bethesda, MD) was used to measure the percentage of the fibrotic area within the left ventricle. The percentage of the fibrotic area within the left ventricle was determined by dividing the circumference of the fibrotic tissue by the total left ventricular circumference across consecutive myocardial slices.

### Senescent Cell Models

To induce fibroblast senescence, cells at ≈70% confluence were exposed to H_2_O_2_ treatment (100 µM) along with serum starvation for 96 h according to previous studies (Sigma‒Aldrich).^[^ ^56^, ^57^ ^]^ Senescence was confirmed through western blot and qPCR analysis of p53, p21, and p16 levels, along with the detection of SA‐β‐gal. The viability of the fibroblasts was also assessed using the CCK‐8 assay. In the H_2_O_2_ + si‐Kdm4a experimental group, fibroblasts were first transfected with the siRNA against Kdm4a (si‐Kdm4a) before exposure to H_2_O_2_. For subsequent coculture experiments, H_2_O_2_‐induced senescent fibroblasts, with or without prior transfection, were placed on an insert membrane, after which the medium was replaced with fresh medium. These cells were then cocultured with normal fibroblasts plated on the well surface, establishing a coculture system that was maintained for an additional 36–48 h.

### ChIP‐seq and ChIP‐qPCR

ChIP assays were performed using a ChIP kit (Millipore) according to the manufacturer's guidelines, which enabled the acquisition of ChIP‐enriched DNA. Fibroblasts were exposed to 1% freshly prepared formaldehyde for 10 min at room temperature to facilitate the establishment of DNA‒protein crosslinks, after which the reaction was stopped by an incubation with glycine (0.125 m) for 5 min. The crosslinked cells were then harvested and lysed in ChIP lysis buffer, after which the chromatin was fragmented into segments spanning 200 to 300 base pairs. Immunoprecipitation was conducted with specific antibodies against Kdm4a or H3K9me3 (Abcam), with IgG used as a control. Protein Ggarose/Sepharose was used to precipitate the endogenous DNA‒protein complexes. The purified DNA fragments were analyzed by quantitative PCR to analyze target gene promoters (within 2000 bp upstream of the TSS) or subjected to ChIP‐seq. The primer‐specific sequences for the promoter region can be found in Table S2 (Supporting Information). ChIP‐seq libraries were constructed using the NEBNext ChIP‐seq Library Prep Master Mix Set for the Illumina system, and subsequent sequencing was performed via the HiSeq 4000 sequencer. Low‐quality reads were removed from the raw data using FastQC. The clean data were mapped to a reference genome using Bowtie2, and MACS2 was utilized to identify regions of significantly enriched peaks. The default setting was used to profile the binding regions and visualize the peaks using the Integrative Genomics Viewer (IGV). To annotate the peaks, we employed the R package ChIPseeker. The differential enrichment of genes was calculated based on the average peaks of all active regions in the gene and within the gene margin.

### Drug Treatment

The Kdm4a inhibitor (ML324, APExBio) was solubilized in diluted DMSO. The mice were randomly divided into different experimental groups. Intraperitoneal injections of ML324 (4 mg kg^−1^) were administered every 2 days, beginning on day 1 and continuing until day 28. The dose and mode of administration of ML324 were determined based on previous studies.^[^ ^29^ ^]^

### RNA Sequencing and Analysis

Primary cultured fibroblasts were transfected with siRNA against Kdm4a (si‐Kdm4a) or a scrambled siRNA control (si‐NC). After transfection, RNA was extracted from the cells using TRIzol reagent (Thermo), according to the manufacturer's protocol. The extracted RNA was subsequently subjected to mRNA isolation with oligo (dT) magnetic beads. We then prepared RNA libraries for RNA‐seq, followed by sequencing on the Illumina NovaSeq 6000 platform. During the data filtering phase, adaptor sequences, contaminants, and low‐quality reads were removed from the raw data. The clean reads were mapped to the mouse genome (mm10) using HISAT2. The fragments per kilobase of exon model per million mapped fragments (FPKM) values for each gene were calculated using Cufflinks. Additionally, the read counts for each gene were obtained through HTSeq‐count. Differentially expressed genes (DEGs) were identified using the DESeq2 package with standard *p* values < 0.05 and |log2FC| > 1. Gene Ontology (GO) enrichment, Kyoto Encyclopedia of Genes and Genomes (KEGG) pathway enrichment, and Gene Set Enrichment Analysis (GSEA) were conducted using the clusterProfiler package. Pearson's correlation coefficients were calculated to assess the correlations between genes.

### RNA Sequencing and Analysis—ssGSEA Analysis

Gene set variation analysis (GSVA), a specialized gene set enrichment method for estimating the variation in gene set enrichment across a dataset of expression samples, was performed using the “GSVA” R package.^[^ ^58^ ^]^ The ssGSEA method was employed to compute enrichment scores for SIPS gene signatures in individual samples. Pearson's correlation analysis was then conducted to examine the relationship between SIPS scores and gene expression levels.

### ScRNA‐Seq Data Analysis—Data Acquisition

Publicly available scRNA‐seq data from cardiac interstitial cells at homeostasis and adult mice with 3‐, 5‐, 7‐, 14‐ and 28‐days after MI were acquired from the European Nucleotide Archive database (ArrayExpress: E‐MTAB‐7895).^[^ ^59^ ^]^ CellRanger (7.0.1) was utilized to generate a single‐cell gene expression matrix from the data of this project.

### ScRNA‐Seq Data Analysis—Quality Control and Normalization

The analysis of the single‐cell gene expression matrix was performed via Seurat (4.0.2), an R package specifically designed for the analysis of single‐cell RNA sequencing data. During the quality control process, low‐quality cells were excluded by filtering out cells with fewer than 200 expressed genes or with mitochondrial reads exceeding 5%. Additionally, genes expressed in fewer than three cells were also removed. The count matrix for the remaining cells was subsequently normalized with Seurat's normalization data function. The top 2000 highly variable genes were detected using the FindVariableFeatures function with the “vst” method. The ScaleData function was utilized on the normalized data.

### ScRNA‐Seq Data Analysis—Unsupervised Clustering Analysis and Cell Annotation

The highly variable genes were subjected to principal component analysis (PCA). To address batch effects and integrate scRNA‐seq data, we utilized the Harmony R package, an algorithm that projects cells into a shared embedding in which cells are grouped by cell type rather than dataset‐specific conditions. Unsupervised cell clustering was conducted utilizing the top 20 principal components with the Seurat functions FindNeighbors and FindClusters. For data visualization, we used t‐distributed stochastic neighbor embedding (tSNE) to project the cells onto a 2D space, with the aligned canonical correlation analysis as the underlying method. To identify genes specific to each cluster, we employed the FindAllMarkers function, utilizing a Wilcoxon rank‐sum test. Manual annotation of cell types was performed based on the highly specific marker genes associated with each cluster.

### ScRNA‐Seq Data Analysis—Stress‐Induced Premature Senescence Analysis

To investigate stress‐induced premature senescence in MI, we defined stress‐induced premature senescence (SIPS) gene set comprising 58 genes identified from the CellAge Database^[^ ^60^ ^]^ as being associated with the induction of stress‐induced premature senescence. The R package AUCell was employed to score the SIPS genes. The input genes were ranked based on their log2FC values, and the area under the curve (AUC) was calculated as the enrichment score to assess the key subset within the expressed genes of each cell, with higher scores indicating greater enrichment. The AUCell analysis utilized three main functions: “AUCell_buildRankings” for gene ranking, “AUCell_calcAUC” for calculating enrichment scores, and “AUCell_exploreThresholds” for determining the optimal cutoff. Ultimately, the optimal threshold obtained from the Global_k1 analysis was used to categorize cells into SIPS and non‐SIPS groups. To identify differentially expressed genes (DEGs) between stress‐induced premature senescent cells and nonstress‐induced premature senescent cells, we utilized the FindAllMarkers function with the MAST method. DEGs were determined based on standard *p* values < 0.05 and average log 2 fold change |avg_log2FC| > 0.15.

### ScRNA‐Seq Data Analysis—Ligand‒Receptor Interaction Analysis

To explore the potential interactions between SIPS fibroblasts and non‐SIPS fibroblasts, we employed the ligand‒receptor interaction tool NicheNet.^[^ ^61^ ^]^ SIPS fibroblasts were defined as sender cells, whereas non‐SIPS fibroblasts were designated as receptor cells. The gene set of interest comprised genes that were differentially expressed within the interacting cell populations. The FindAllMarkers function, with the default parameters, was used to identify DEGs, which were further filtered with a P value < 0.05 and an average log (fold change) > 0.15. Ligand activities were predicted using the predict_ligand_activities function in NicheNet, and the ligands were ranked according to the Pearson correlation coefficient. NicheNet can prioritize the interaction potential of ligands with receptors and assess the regulatory potential of ligands on target genes.

### Statistical Analysis

The results were statistically analyzed using SPSS 22.0 software. All the data are shown in the graphs as the means ± SDs. For the statistical comparison of 2 groups, an unpaired, 2‐tailed Student's *t*‐test was used. One‐way ANOVA, followed by Bonferroni's multiple comparisons test, was used for comparisons of more than 2 groups. The *p* value was calculated, and *p* < 0.05 indicated statistical significance.

## Conflict of Interest

The authors declare no conflict of interest.

## Author Contributions

M. J. and C. L. contributed equally to this work. H.Z., J.P. B. and M. J. conceived and designed the study. M. J., C.L.L., and Z.Y.W. performed the experiments and and collected the data. G.Q.W., Z.Q. T., Z.W.Y., and J.F.X. analyzed and interpreted data. H.Z., J.P.B., M.J., and G.J.C. wrote the final manuscript. S.L.H., X.Z.L., Y.M.C., W.J.L., Y.J.C. and Y.L. L. supervised the experiments. All authors read and approved the final version of the manuscript.

## Supporting information



Supporting Information

## Data Availability

All data are available in the main text or the supplementary materials.
