# Supplementary material for: Inhibiting the Histone Demethylase Kdm4a Restrains Cardiac Fibrosis After Myocardial Infarction by Promoting Autophagy in Premature Senescent Fibroblasts
Source: Adv Sci (Weinh). 2025 Apr 15;12(21):2414830. doi: 10.1002/advs.202414830 (PMC12140349; doi:10.1002/advs.202414830)

**SUPPLEMENTAL MATERIALS**

**Title: Inhibiting the histone demethylase Kdm4a restrains cardiac fibrosis after myocardial infarction by promoting** **autophagy in premature senescent fibroblasts**

**Authors:** Ming Jin, MD, PhD ^1,3#^; Chuling Li, MD^2#^; Zhaoyi Wu, MD^1,3^; Zhenquan Tang, MD, PhD^1,3^; Jingfang Xie, MD, PhD^5^; Guoquan Wei, MD, PhD^1,3^; Zhiwen Yang, MD, PhD^1,3;^ Senlin Huang, MD, PhD^1,3^; Yijin Chen, MD^1,3^; Xinzhong Li, MD, PhD^1,3^; Yanmei Chen, MD, PhD^1,3^; Wangjun Liao, MD, PhD^4^; Yulin Liao,MD, PhD^2^; Guojun Chen, MD, PhD^1,3*^; Hao Zheng, MD, PhD^1,3*;^ Jianping Bin, MD, PhD^1,2,3*^

^1^Department of Cardiology, State Key Laboratory of Organ Failure Research, Nanfang Hospital, Southern Medical University, 510515, Guangzhou, China;

^2^Cardiovascular Center, the Sixth Affiliated Hospital, School of Medicine, South China University of Technology, Foshan, 528200, China;

^3^Guangdong Provincial Key Laboratory of Shock and Microcirculation, 510515, Guangzhou, China;

^4^Department of Oncology, Nanfang Hospital, Southern Medical University, 510515, Guangzhou, China;

^5^Guangdong Provincial Geriatrics Institute, Guangdong Provincial People’s Hospital (Guangdong Academy of Medical Sciences), Southern Medical University, Guangzhou 510080, China.

Ming Jin and Chuling Li contributed equally to this work.

**Short title:** Inhibiting Kdm4a restrains cardiac fibrosis

^#^Corresponding author: Jianping Bin, MD, PhD, Department of Cardiology, State Key Laboratory of Organ Failure Research, Nanfang Hospital, Southern Medical University, 1838 Guangzhou Avenue North, Guangzhou, 510515, China. China Tel: +862061642365; Fax: +862087712332; E-mail: [jianpingbin@126.com](mailto:jianpingbin@126.com) or [jianpingbin@hotmail.com](mailto:jianpingbin@hotmail.com).

**Supplementary Table 1: Antibodies for western blots and chromatin immunoprecipitation assay and immunofluorescence staining.**

|  | Vendor or  Source | Catalog # | Dilute  Proportion |
| --- | --- | --- | --- |
| anti-p16 | Abcam | ab189034 | 1:500 |
| anti-p21 | Proteintech | 10355-1-AP | 1:2000 |
| anti-p21 | Abcam | ab188224 | 1:1000 |
| anti-p53 | Proteintech | 10442-1-AP | 1:2000 |
| anti-γH2AX | Santa Cruz Biotechnology | sc-517348 | 1:500 |
| anti-GAPDH | Proteintech | 10494-1-AP | 1:5000 |
| anti-Kdm4a | Santa Cruz Biotechnology | sc-271210 | 1:500 |
| anti-vimentin | Invitrogen | PA1-10003 | 1:200 |
| anti-vimentin | Abcam | ab8978 | 1:200 |
| anti-α-SMA | Proteintech | 14395-1-AP | 1:2000 |
| anti-α-SMA | Abcam | ab7817 | 1:1000 |
| anti-Periostin | Santa Cruz Biotechnology | sc-398631 | 1:500 |
| anti-CollagenI | Proteintech | 14695-1-AP | 1:2000 |
| anti-CollagenI | Invitrogen | PA5-29569 | 1:200 |
| anti-CollagenⅢ | Proteintech | 22734-1-AP | 1:2000 |
| anti-H3 | Abcam | ab1791 | 1:2000 |
| anti-H3K4me3 | Abcam | ab8580 | 1:3000 |
| anti-H3K9me3 | Abcam | ab8898 | 1:3000 |
| anti-H3K27me3 | ABclonal | a2363 | 1:1000 |
| anti-H4K20me3 | ABclonal | a2372 | 1:500 |
| anti-Trim44 | Proteintech | 11511-1-AP | 1:500 |
| anti-Beclin1 | Proteintech | 11306-1-AP | 1:1000 |
| anti-LC3 | Cell Signaling Technology | 3868 | 1:1000 |
| anti-p62 | Proteintech | 18420-1-AP | 1:2000 |
| anti-Nexn | Abcam | ab233267 | 1:1000 |
| anti-Podn | Proteintech | 15014-1-AP | 1:500 |
| anti-Tublin | Proteintech | 11224-1-AP | 1:5000 |
| anti-Svil | Santa Cruz Biotechnology | sc-53556 | 1:500 |
| anti-TGF-β1 | Invitrogen | MA1-21595 |  |
| Goat anti-mouse  IgG HRP | Santa Cruz Biotechnology | sc-2005 | 1:10000 |
| Goat anti-rabbit  IgG HRP | Santa Cruz Biotechnology | sc-2004 | 1:10000 |
| Goat Anti-Chicken IgY H&L | Abcam | ab150169 | 1:200 |

**Supplementary Table 2: The primers used in this study.**

| Experiments | Gene name |  | Primers Sequence (5’-3’) |
| --- | --- | --- | --- |
| qPCR | p16 | + | CCGATTCAGGTGATGATGAT |
|  |  | - | CGCACGATGTCTTGATGT |
|  | p21 | + | TTCCTTGCCACTTCTTACC |
|  |  | - | ACTGCTTCACTGTCATCC |
|  | p53 | + | ACAAGAAGTCACAGCACAT |
|  |  | - | ATAGGTCGGCGGTTCAT |
|  | IL-6 | + | CCGCTATGAAGTTCCTCTC |
|  |  | - | GGTATCCTCTGTGAAGTCTC |
|  | IL-8 | + | GAGACCTGAGAACAAGAGAA |
|  |  | - | ATCCATACACCAGACTAACG |
|  | IL-1β | + | CTTCAGGCAGGCAGTATC |
|  |  | - | CAGCAGGTTATCATCATCATC |
|  | MCP-1 | + | CAATGAGTAGGCTGGAGAG |
|  |  | - | GAAGTGCTTGAGGTGGTT |
|  | MMP-2 | + | CTACACCTACACCAAGAACT |
|  |  | - | CCTCATACACAGCGTCAA |
|  | MMP-9 | + | GCGTGTCTGGAGATTCGACTTG |
|  |  | - | ACTGCAGGAGGTCGTAGGTCAC |
|  | Vegfa | + | GGCTGCTGTAACGATGAA |
|  |  | - | CTGCTGTGCTGTAGGAAG |
|  | Kdm4a | + | CGCTACATTGCCTACATTG |
|  |  | - | GAACTCACGAACGGTCAT |
|  | Kdm4b | + | CACTAACTTCGCCACACT |
|  |  | - | CTTGCTTCCATTGCTCATAG |
|  | Kdm4c | + | TCACGCAATACAACATCCA |
|  |  | - | ACACTCTTCTTCAACCACAT |
|  | Kdm4d | + | CCTGAGCGATATGAACTGT |
|  |  | - | GACGACGACCAAGACTAC |
|  | Trim44 | + | AGAAGACAGCGAGGAAGA |
|  |  | - | TGGACACAGGACACAGAT |
|  | Pcdhgb4 | + | ATCAGACGGTAGTAAGTACCCTG |
|  |  | - | GGTCACTAGGACCTGTATCTGT |
|  | Sptbn2 | + | GGCCGAATGCGGATTCACT |
|  |  | - | GGTGGTTTCCGTCCACAATGT |
|  | Podn | + | GACTGTCCCCGAGATTGTGC |
|  |  | - | CAGGTCGCCTGGAAACTCA |
|  | Nexn | + | GGAAAATACAGCGCGAATTAGC |
|  |  | - | CTCTGATGCGGACTCGGTTC |
|  | Svil | + | GTCCCAAAGAGACATTCGAGAAA |
|  |  | - | CTGTGTGTGTGAACGGTCCT |
|  | Amn | + | CAGCTCTGTGCAATGACTCG |
|  |  | - | CGGGAACTGAACCGCATCTC |
|  | Lad1 | + | ACGAAGAACGCCTAAAGAACAG |
|  |  | - | AGGGGCAGGCTTTCTCAGA |
|  | Dmrta2 | + | CAGGCTCGGAGAACGGAGA |
|  |  | - | GAACCCGATTCGGAACCCA |
|  | Gata3 | + | AAGCTCAGTATCCGCTGACG |
|  |  | - | GTTTCCGTAGTAGGACGGGAC |
|  | GAPDH | + | AGGTCGGTGTGAACGGATTTG |
|  |  | - | GGGGTCGTTGATGGCAACA |
| Experiments | Gene name |  | Primers Sequence (5’-3’) |
|  |  |  |  |
| ChIP | Svil | + | TGAGGAAGAAGGAGATGAGA |
|  |  | - | CTGTGTTGAATACGCATAGG |
|  | Podn | + | TCTGTCTACTTCCATCTCCT |
|  |  | - | ACCTGACTGAACTCCAAGA |
|  | Trim44 | + | ATGATGGTGTATGGCTTAGG |
|  |  | - | AGGAGTGGCTTAACTATGTG |
|  | Sptbn2 | + | CCTACTCTAACCACAACCTAA |
|  |  | - | AACTACCTCACTCCATCCA |
|  | Amn | + | CCTACTCTAACCACAACCTAA |
|  |  | - | AACTACCTCACTCCATCCA |
|  | Lad1 | + | GACCTCTTCACTTGCTCTAT |
|  |  | - | CCTCCTCCTTATCCTCACA |
|  | Nexn | + | AAGTAAGCCGCAGGTAAC |
|  |  | - | CCGATGAAGGAGGATAGAAG |

**Supplementary Table 3. Kdm4a and Trim44 siRNA sequences.**

| siRNA 1 Kdm4a, sense | 5’-CGAACATCCTACGACGATA-3’ |
| --- | --- |
| siRNA 2 Kdm4a, sense | 5’-CACCGAGACCTTCTACGAA-3’ |
| siRNA 3 Kdm4a, sense | 5’-GAATGGATGTGCTCTCGAT-3’ |
| siRNA 1 Trim44, sense | 5’-GCCTTTGAAGAATTAAGAAGC-3’ |
| siRNA 2 Trim44, sense | 5’-GCAATGATAGAGTTGGTGGAA-3’ |
| siRNA 3 Trim44, sense | 5’-GAAUCAGUCGGAUACUCAUAG-3’ |

**Supplementary Table 4. ELISA kit** **.**

|  | Vendor or  Source | Catalog # |
| --- | --- | --- |
| TGF-β1 | Elabscience | E-EL-0162 |
| IL-6 | Cloud Clone Corp | SEA079Mu |
| IL-1β | Elabscience | E-EL-M0037 |
| TNF-α | Cloud Clone Corp | SEA133Mu |
| MMP-2 | Cloud Clone Corp | SEA100Mu |
| Fibronectin | Cloud Clone Corp | SEA037Mu |

**Figure S1**


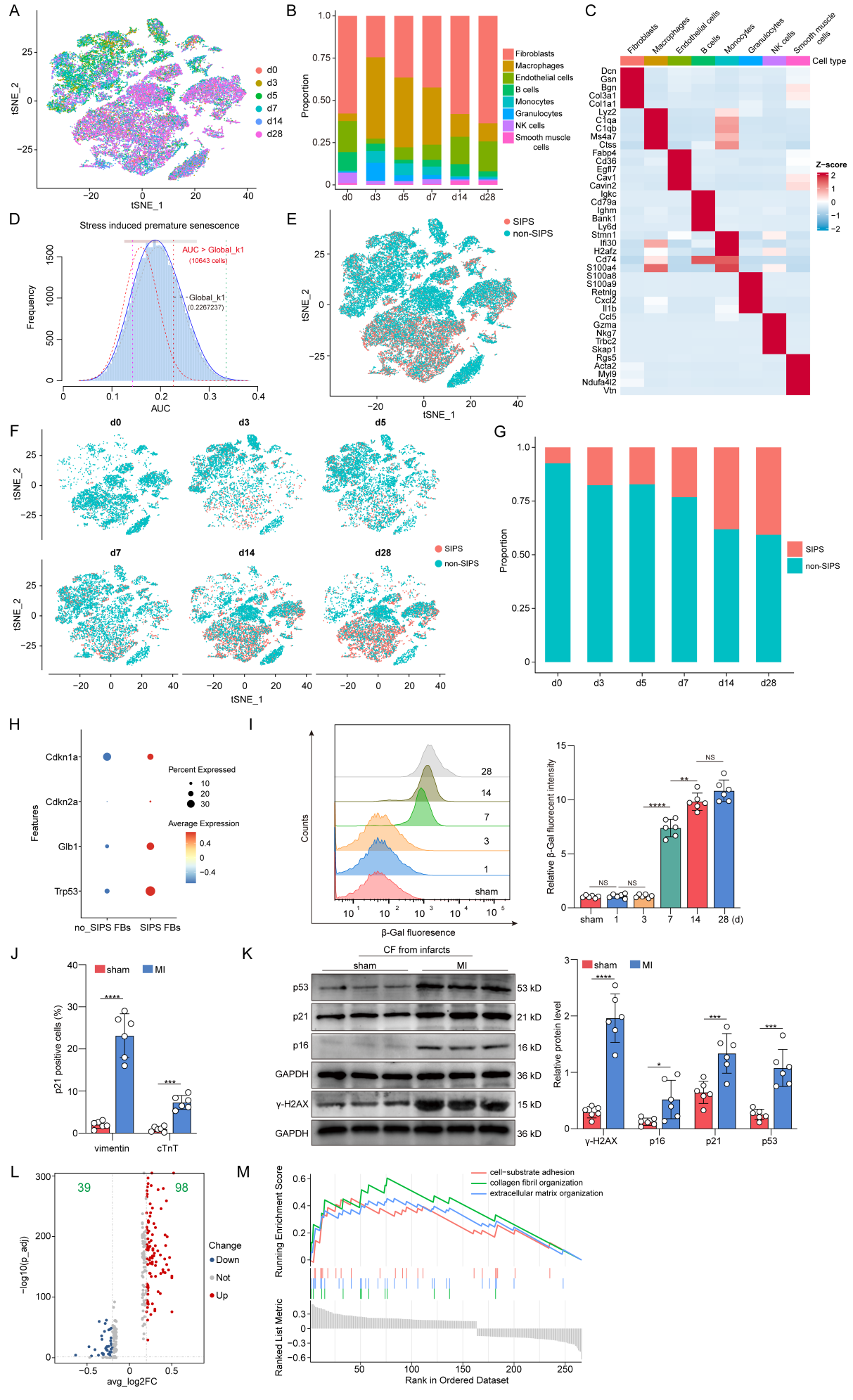


**Supplementary figure 1. Analysis of the cardiac cellulome using scRNA-seq.**

A. A total of 42,049 cells derived from various conditions were integrated and visualized on a tSNE plot.

B. Bar plots depicting the distribution of 8 cell types across different time points.

C. Heatmap showing the expression of marker genes of 8 cell types.

D. Columnar distribution plots illustrating the frequency of AUC scores for the SIPS gene set. A total of 10,643 cells exceeding the 'Global_k1' threshold of the AUC score of SIPS.

E. A tSNE projection of SIPS and non-SIPS cells based on the 'Global_k1' threshold in the AUC score. Cells exceeding the 'Global_k1' threshold were clustered as SIPS cells, while the remaining cells were classified as non-SIPS cells.

F. tSNE plots illustrating the variations between SIPS and non-SIPS cells across different time points.

G. Bar plots depicting the distribution of SIPS and non-SIPS cells across different time points.

H. Dot plots showing the expression of Cdkn1a, Cdkn2a, Glib1 and Trp53 in SIPS fibroblasts and non-SIPS fibroblasts.

I. Flow cytometry assay sort the number of senescent cells after MI sing β-galactosidase (β-Gal)  probe.

J. Representative densitometric analysis of senescence-associated markers p21 in MI model hearts at 14 days post-MI. (n = 6 /group)

K. Western blot analysis of the protein levels of p16, γH2AX, p21, and p53 in isolated fibroblasts from the hearts of mice with MI and in control fibroblasts. (n = 6/group)

L. Volcano plot illustrating differentially expressed genes in SIPS fibroblasts compared to non-SIPS fibroblasts. A | avglog2FC |>0.15 and a p value<0.05 were considered indicative of significant differential expression.

M. GSEA of ‘cell-substrate adhesion’, ‘extracellular matrix organization’ and ‘collagen fibril organization’ in SIPS fibroblasts compared to non-SIPS fibroblasts. The x-axis represents the ranked gene list based on differential expression, while the y-axis displays the enrichment score.

Data are expressed as the means SD for each group. I was analyzed by one-way ANOVA followed by Tukey’s test. J-K were analyzed by two-tailed unpaired t-test. *P < 0.05, **P < 0.01, ***P < 0.001, ****P < 0.0001.

**Figure S2**


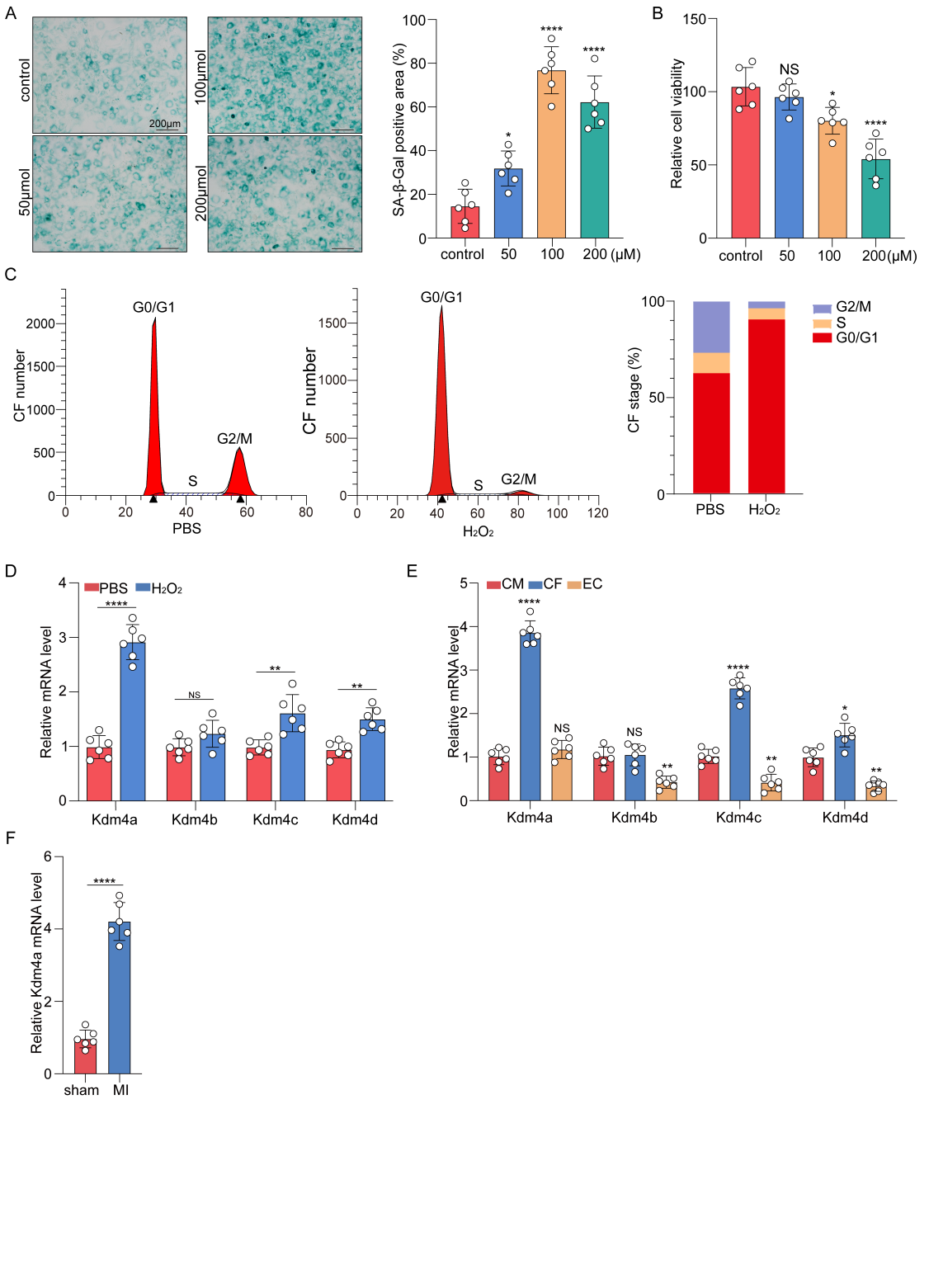


**Supplementary figure 2. H_2_O_2_ induces cellular senescence in cardiac fibroblasts.**

A. SA-β-gal staining in control fibroblasts (control) and H_2_O_2_-induced senescent fibroblasts (H_2_O_2_) at various concentrations (50 μM, 100 μM and 200μM). (n = 6 /group; scale bars = 200 μm)

B. The cytotoxicity of fibroblasts treated with H_2_O_2_ at various concentrations (50 μM, 100 μM, and 200 μM) was assessed by the CCK-8 assay.

C. Cell cycle was assessed by flow cytometry after treatment with H_2_O_2_ for 96 hours and the percentages of fibroblasts in each phase were analysed and shown in the right panel. (n = 6/group)

D. RT-qPCR analysis of the mRNA levels of Kdm4a, Kdm4b, Kdm4c and Kdm4d in control fibroblasts and H_2_O_2_-induced senescent fibroblasts. (n = 6/group)

E. RT-qPCR analysis of Kdm4a, Kdm4b, Kdm4c and Kdm4d mRNA levels in cardiomyocytes (CMs), fibroblasts (FBs) and endothelial cells (ECs). (n = 6/group)

F. RT-qPCR analysis of Kdm4a mRNA levels in isolated fibroblasts from the hearts of mice with myocardial infarction and in control fibroblasts. (n = 6/group)

Data are expressed as the means SD for each group. A, B and E were analyzed by one-way ANOVA followed by Tukey’s test. C, D and F were analyzed by two-tailed unpaired t-test. *P < 0.05, **P < 0.01, ***P < 0.001, ****P < 0.0001.

**Figure S3**


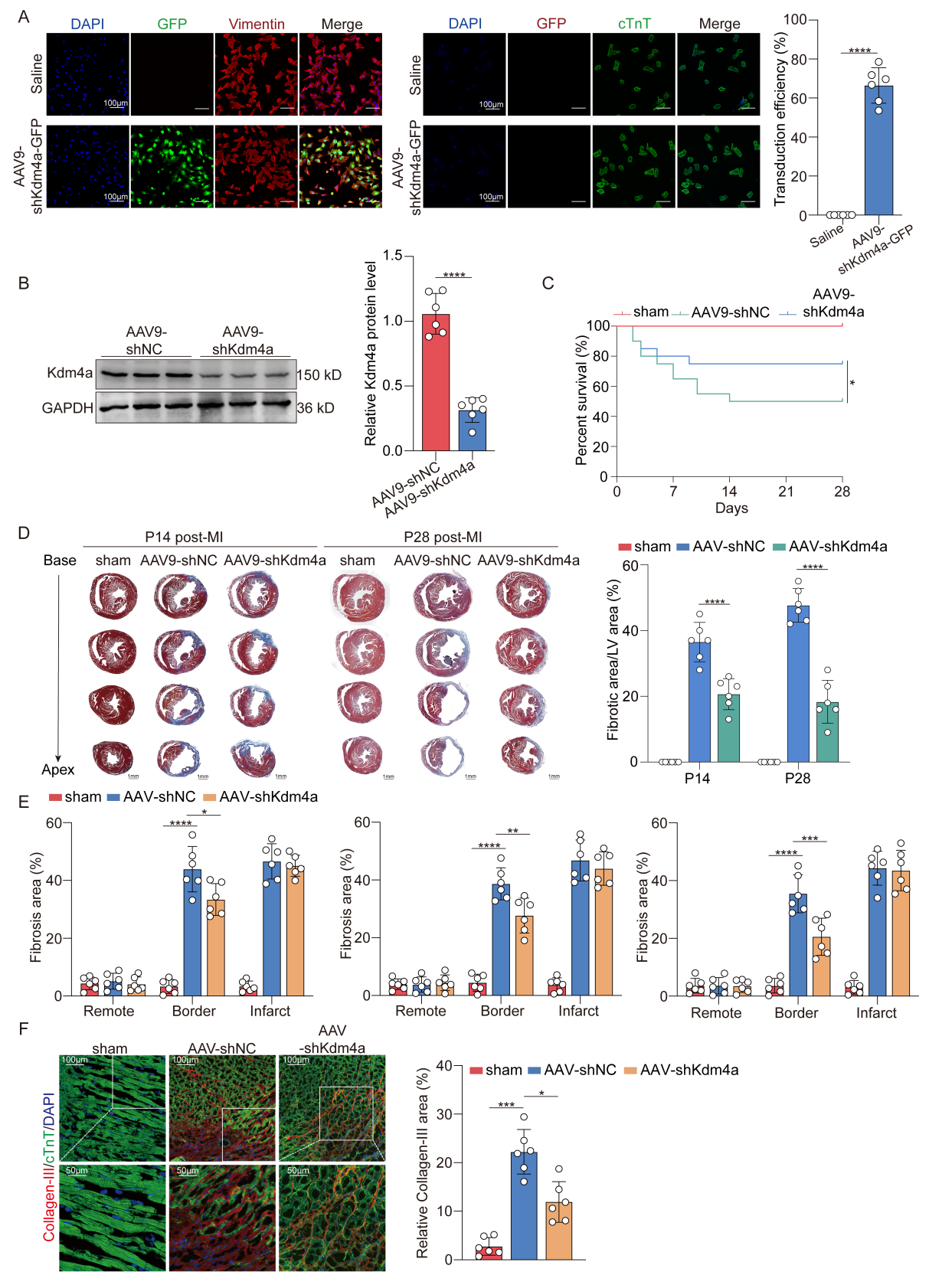


**Supplementary figure 3. AAV-shKdm4a pretreatment prevents cardiac fibrosis after MI in adult mice.**

A. The transduction efficiency of AAV9-shKdm4a in adult fibroblasts and cardiomyocyte in adult mouse. (n = 6 /group; scale bars = 100μm)

B. Western blot analysis and quantification of Kdm4a expression in adult mouse hearts injected with AAV9-NC or AAV9-shKdm4a. (n = 6/group)

C. Kaplan-Meier survival curves in the sham, shNC and shKdm4a groups post-MI. (n =20 /group)

D. Masson's trichrome staining of heart cross sections in adult mice at 14 and 28 days post-MI and quantification of infarct size. (n = 6 /group; scale bars = 1mm)

E. Quantitative analysis of fibrosis area in the myocardium of three different zones in mice from the three groups. (n = 6/group)

F. Immunofluorescence for collagen III in hearts of adult mouse models of MI at 14 days post-MI. (n = 6 /group; scale bars = 100 and 50 μm)

Data are expressed as the means SD for each group. A and B were analyzed by two-tailed unpaired t-test. C was analysed by the log-rank (Mantel-Cox) test. D-F were analyzed by one-way ANOVA followed by Tukey’s test. *P < 0.05, **P < 0.01, ***P < 0.001, ****P < 0.0001.

**Figure S4**


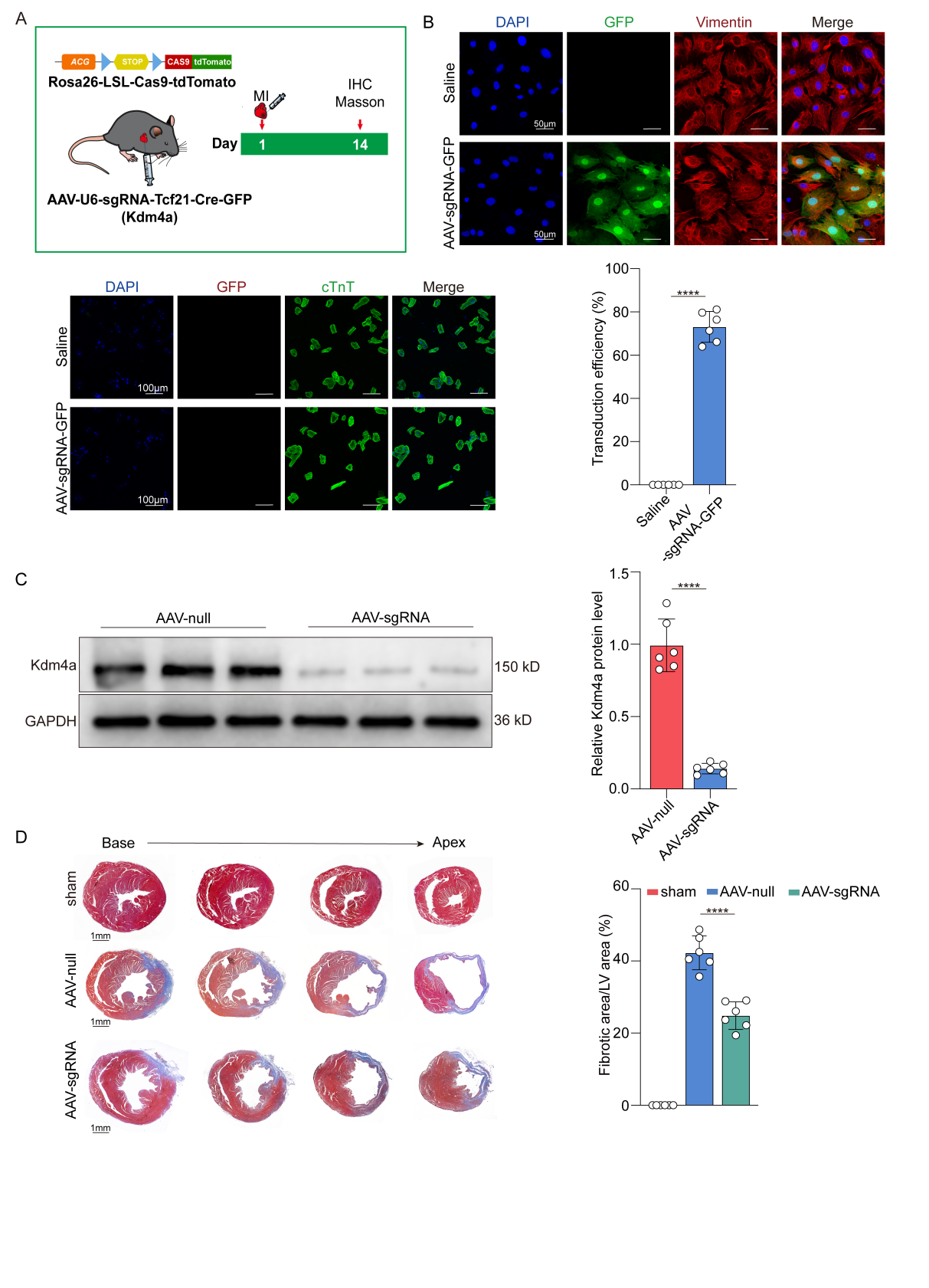


**Supplementary figure 4. AAV-sgRNA pretreatment prevents cardiac fibrosis after MI in adult mice.**

A. Scheme depicting the Cas9-tdTomato mice injected with AAV expressing sgRNA.

B. The transduction efficiency of AAV-sgRNA in adult fibroblasts and cardiomyocyte in adult mouse. (n = 6 /group; scale bars = 50μm and 100μm )

C. Western blot analysis and quantification of Kdm4a expression in adult mouse hearts injected with AAV-null or AAV9-sgRNA. (n = 6/group)

D. Masson's trichrome staining of heart cross sections in adult mice at 14 days post-MI and quantification of infarct size. (n = 6 /group; scale bars = 1mm)

Data are expressed as the means SD for each group. B and C were analyzed by two-tailed unpaired t-test. D were analyzed by one-way ANOVA followed by Tukey’s test. *P < 0.05, **P < 0.01, ***P < 0.001, ****P < 0.0001.

**Figure S5**


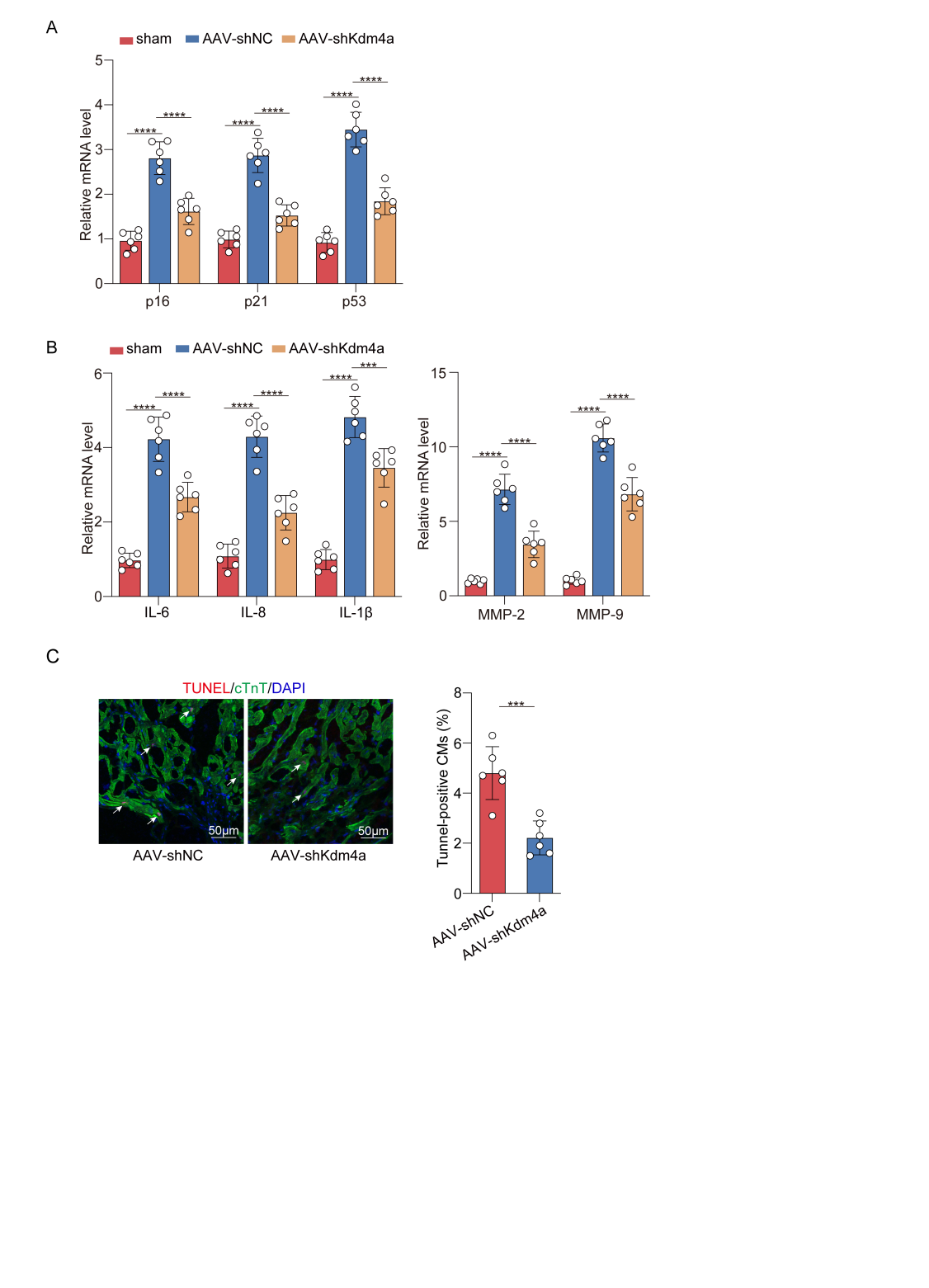


**Supplementary figure 5. Deficiency of Kdm4a inhibits fibroblast senescence after MI in adult mice.**

A-B. RT‒PCR analyzed the expression of senescence-associated markers and SASP factor mRNA levels in each group. RNA levels were normalized to GAPDH. (n = 6 /group)

C. Immunofluorescence for TUNEL staining in hearts of adult mouse models of MI at 14 days post-MI. (n = 6 /group; scale bars = 50 μm)

Data are expressed as the means SD for each group. A-B were analyzed by one-way ANOVA followed by Tukey’s test. C were analyzed by two-tailed unpaired t-test. *P < 0.05, **P < 0.01, ***P < 0.001, ****P < 0.0001.

**Figure S6**


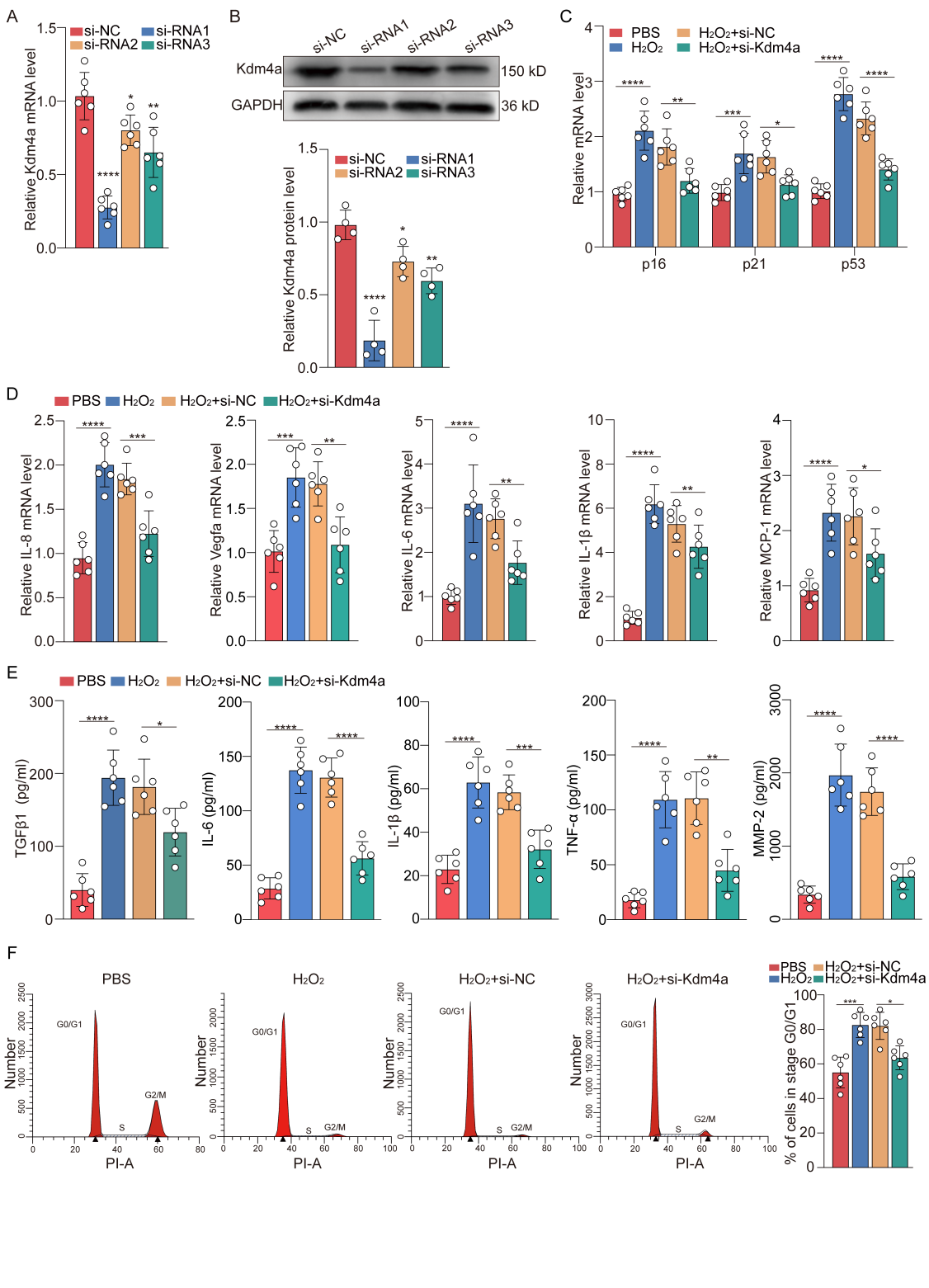


**Supplementary figure 6. Kdm4a deficiency inhibits H_2_O_2_-induced cellular senescence in primary cardiac fibroblasts.**

A. Kdm4a mRNA levels in fibroblasts incubated with three different siRNAs targeting Kdm4a. RNA levels were normalized to GAPDH. (n = 6/group)

B. Kdm4a protein levels in fibroblasts incubated with three different siRNAs targeting Kdm4a. Protein levels were normalized to GAPDH. (n = 6 /group)

C-D. The mouse cardiac fibroblasts were pretreated with si-Kdm4a and then exposed to H_2_O_2_ (100 μM) for an additional 96 h, and then the cells were analyzed. RT‒PCR analyzed the expression of senescence-associated markers and SASP factors mRNA levels in each group. RNA levels were normalized to GAPDH. (n = 6 /group)

E. SASP detection by enzyme-linked immunosorbent assay. ELISAs were performed on conditioned medium collected from fibroblasts incubated in PBS, H_2_O_2_, H_2_O_2_ +si-NC, and H_2_O_2_ +si-Kdm4a. (n = 6/group)

F. Flow cytometry analysis the distribution of the cell cycle and the percentage of cells in the G0/G1 phase. (n = 6 /group)

Data are expressed as the means SD for each group. A-F were analyzed by one-way ANOVA followed by Tukey’s test. *P < 0.05, **P < 0.01, ***P < 0.001, ****P < 0.0001.

**Figure S7**


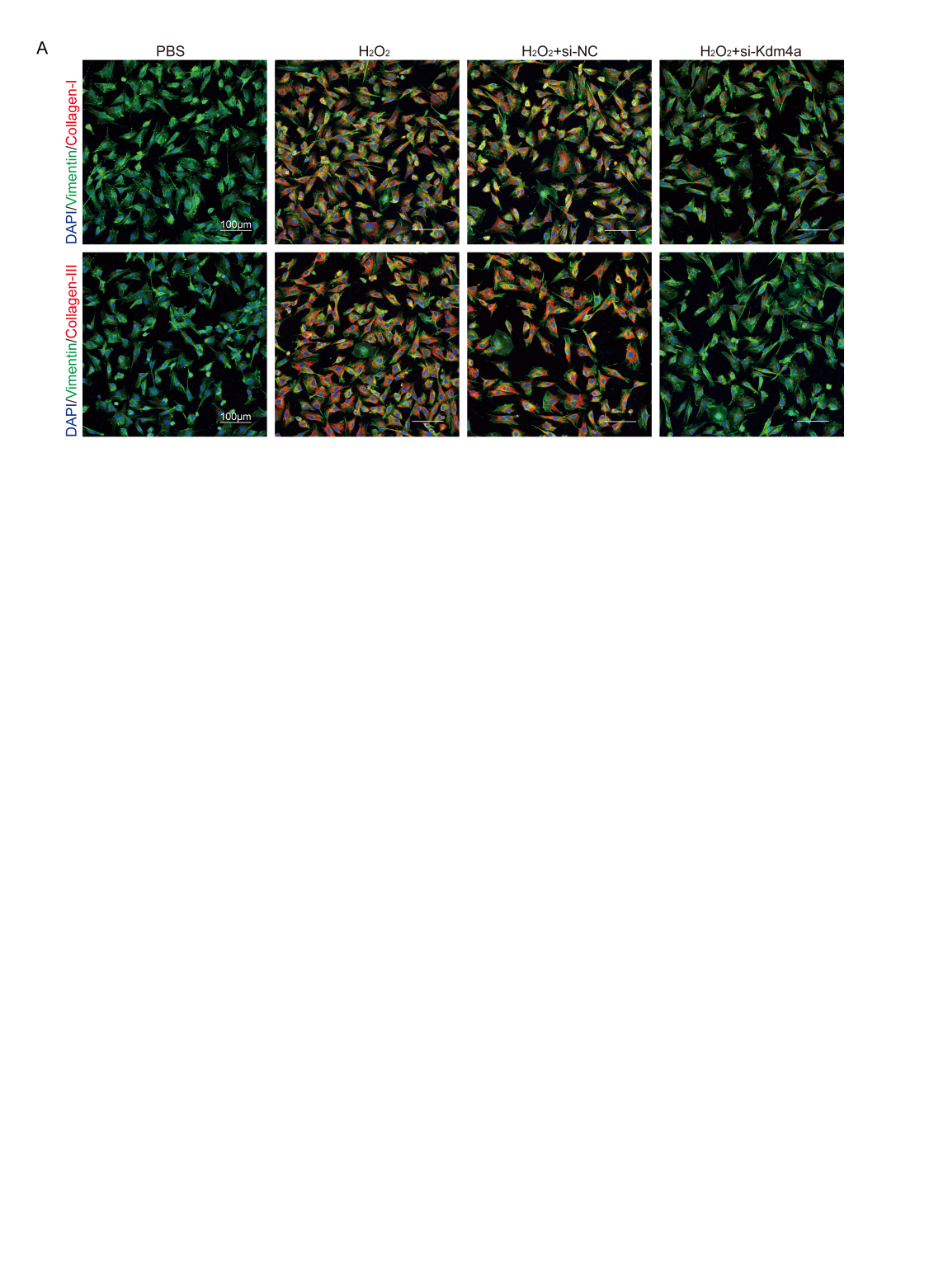


**Supplementary figure 7. Kdm4a deficiency suppresses H_2_O_2_-induced extracellular matrix (ECM) production in cardiac fibroblasts.**

A. Representative images of collagen I and collagen III immunostaining in fibroblasts cocultured with fibroblasts from PBS, H_2_O_2_, H_2_O_2_ + si-NC or H_2_O_2_ + si-Kdm4a. (scale bars = 100 µm)

**Figure S8**


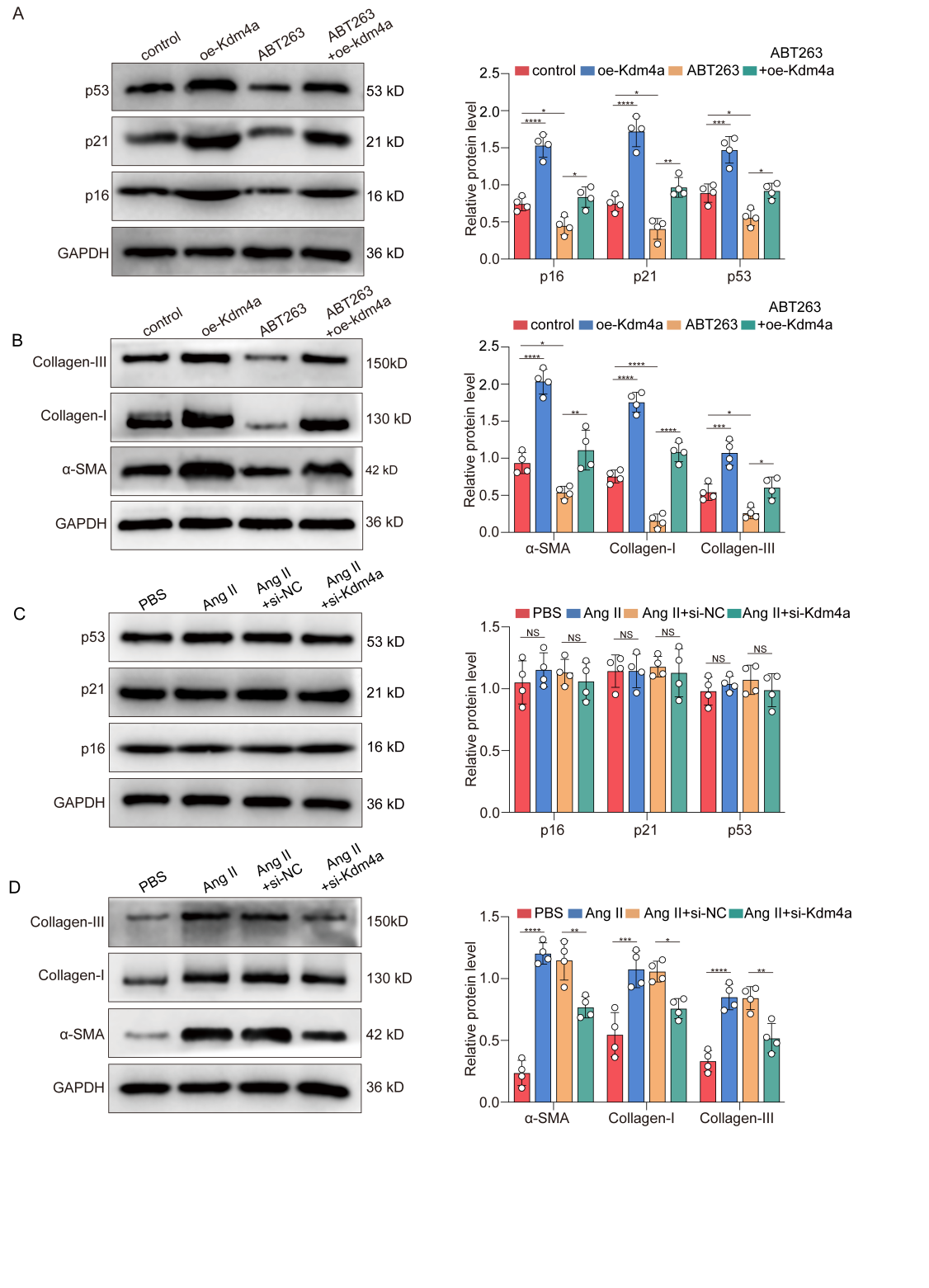


**Supplementary figure 8. Kdm4a deficiency suppresses fibroblast activation and ECM production by suppressing fibroblast senescence.**

A. Western blot analysis and quantification of p16, p21 and p53 protein levels in fibroblasts to evaluate the effects of Kdm4a. GAPDH was used as a loading control. (n = 4/group)

B. Western blot analysis and quantification of expression of fibrotic markers α-SMA, collagen I and collagen III expressions in fibroblasts cocultured with fibroblasts from OE-NC + PBS, OE-Kdm4a+ PBS, OE-NC + ABT-263 or OE-Kdm4a+ ABT-263. GAPDH was used as a loading control. (n = 4/group)

C. Western blot analysis and quantification of p16, p21 and p53 protein levels in fibroblasts to evaluate the effects of Kdm4a. GAPDH was used as a loading control. (n = 4/group)

D. Western blot analysis and quantification of expression of fibrotic markers α-SMA, collagen I and collagen III expressions in fibroblasts cocultured with fibroblasts from PBS, Ang II, Ang II + si-NC or Ang II + si-Kdm4a. GAPDH was used as a loading control. (n = 4/group)

Data are expressed as the means SD for each group. A-D were analyzed by one-way ANOVA followed by Tukey’s test. *P < 0.05, **P < 0.01, ***P < 0.001, ****P < 0.0001.

**Figure S9**


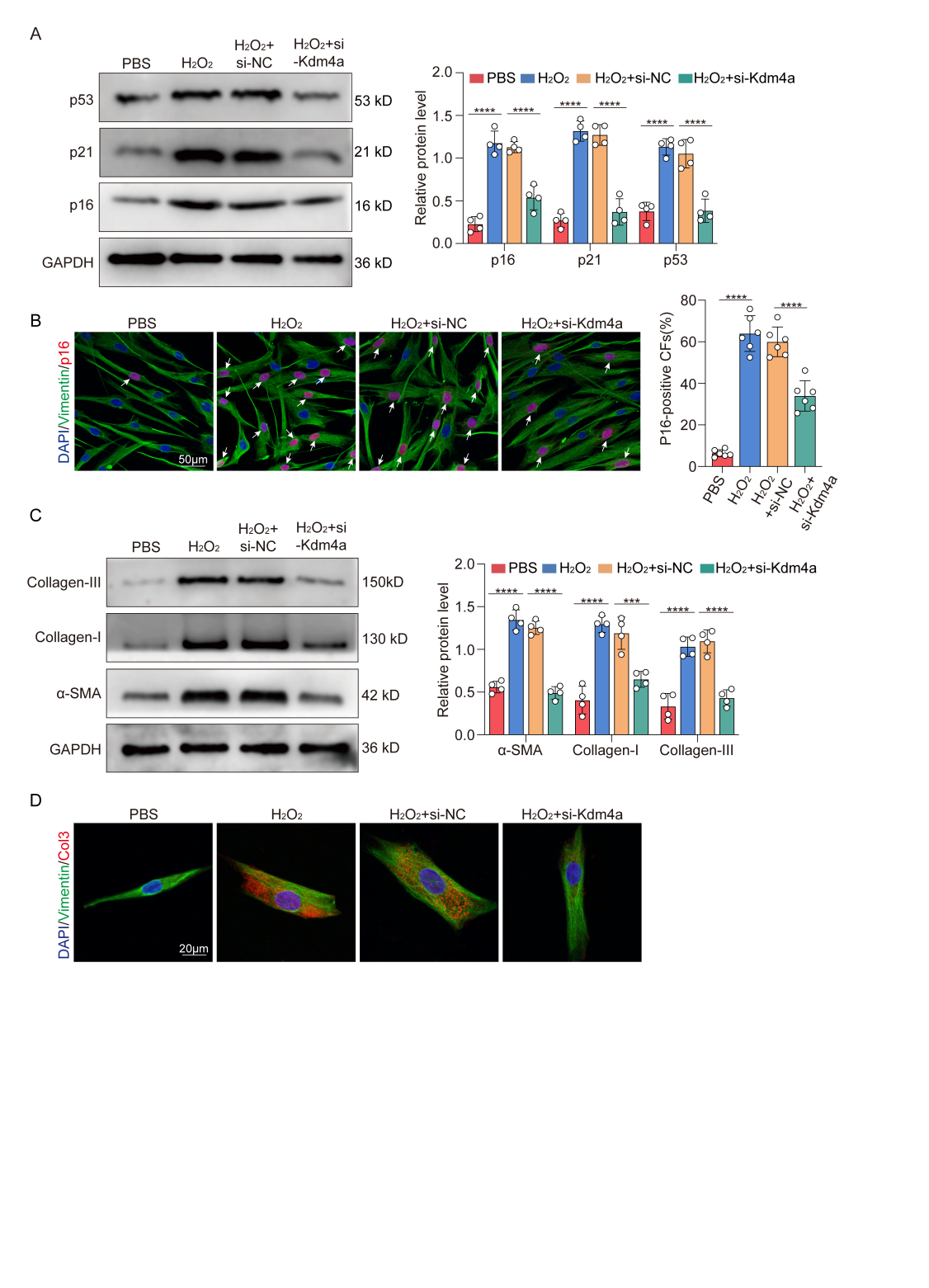


**Supplementary figure 9. Kdm4a deficiency suppresses H_2_O_2_-induced extracellular matrix (ECM) production in human cardiac fibroblasts.**

A. Western blotting and densitometric analysis of the protein levels of p16, p21 and p53 in each group. (n = 4 /group)

B. Representative immunofluorescence images for p16 in fibroblasts. Fibroblasts are stained with vimentin, nuclei are labelled with DAPI. (scale bars = 50 µm; n = 6/group)

C. Western blotting of the protein levels of fibrotic markers α-SMA, collagen I, and collagen III in cultured fibroblasts transfected with siRNA against Kdm4a (si-Kdm4a). (n = 4 /group)

D. Representative images of collagen III immunostaining in fibroblasts cocultured with fibroblasts from PBS, H_2_O_2_, H_2_O_2_ + si-NC or H_2_O_2_ + si-Kdm4a. (scale bars = 20 µm)

Data are expressed as the means SD for each group. A-C were analyzed by one-way ANOVA followed by Tukey’s test. *P < 0.05, **P < 0.01, ***P < 0.001, ****P < 0.0001.

**Figure S10**

**
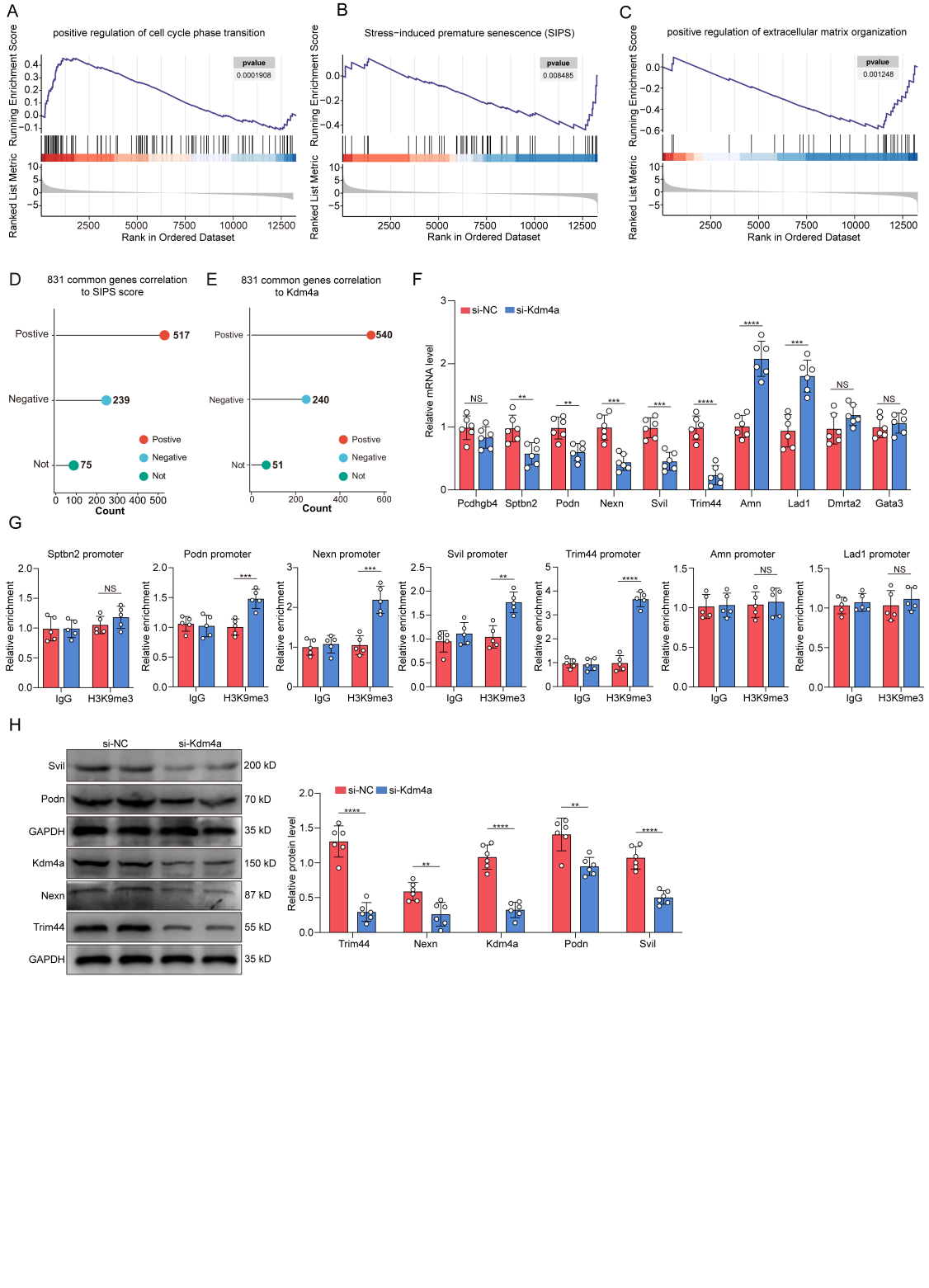
**

**Supplementary figure 10. Identification of Trim44 as the main targeting protein influenced by Kdm4a-mediated H3K9me3 modifications.**

A-C. GSEA of ‘positive regulation of cell cycle phase transition’, ‘negative regulation of SIPS’ and ‘extracellular matrix organization’ in H_2_O_2_-treated fibroblasts following Kdm4a knockdown. The x-axis represents the ranked gene list based on differential expression, while the y-axis displays the enrichment score.

D-E. The number of overlapping 831 genes correlated with SIPS ssGSEA scores or Kdm4a expression was assessed. Genes significantly correlated were identified with a p-value < 0.05 and an absolute value of the Pearson correlation coefficient > 0.8.

F. RT-PCR to confirm the expression level of the indicated genes in fibroblasts regulated by Kdm4a. (n = 5/group)

G. ChIP‐qPCR to confirm changes in H3K9me3 modification on the promoters of the indicated genes in fibroblasts regulated by Kdm4a. (n = 5/group)

H. Western blot analysis and quantification of Svil, Podn, Nexn and Trim44 protein levels in fibroblasts after Kdm4a inhibition. (n = 6/group)

Data are expressed as the means SD for each group. F, G and H were analyzed by two-tailed unpaired t-test. *P < 0.05, **P < 0.01, ***P < 0.001, ****P < 0.0001.

**Figure S11**

**
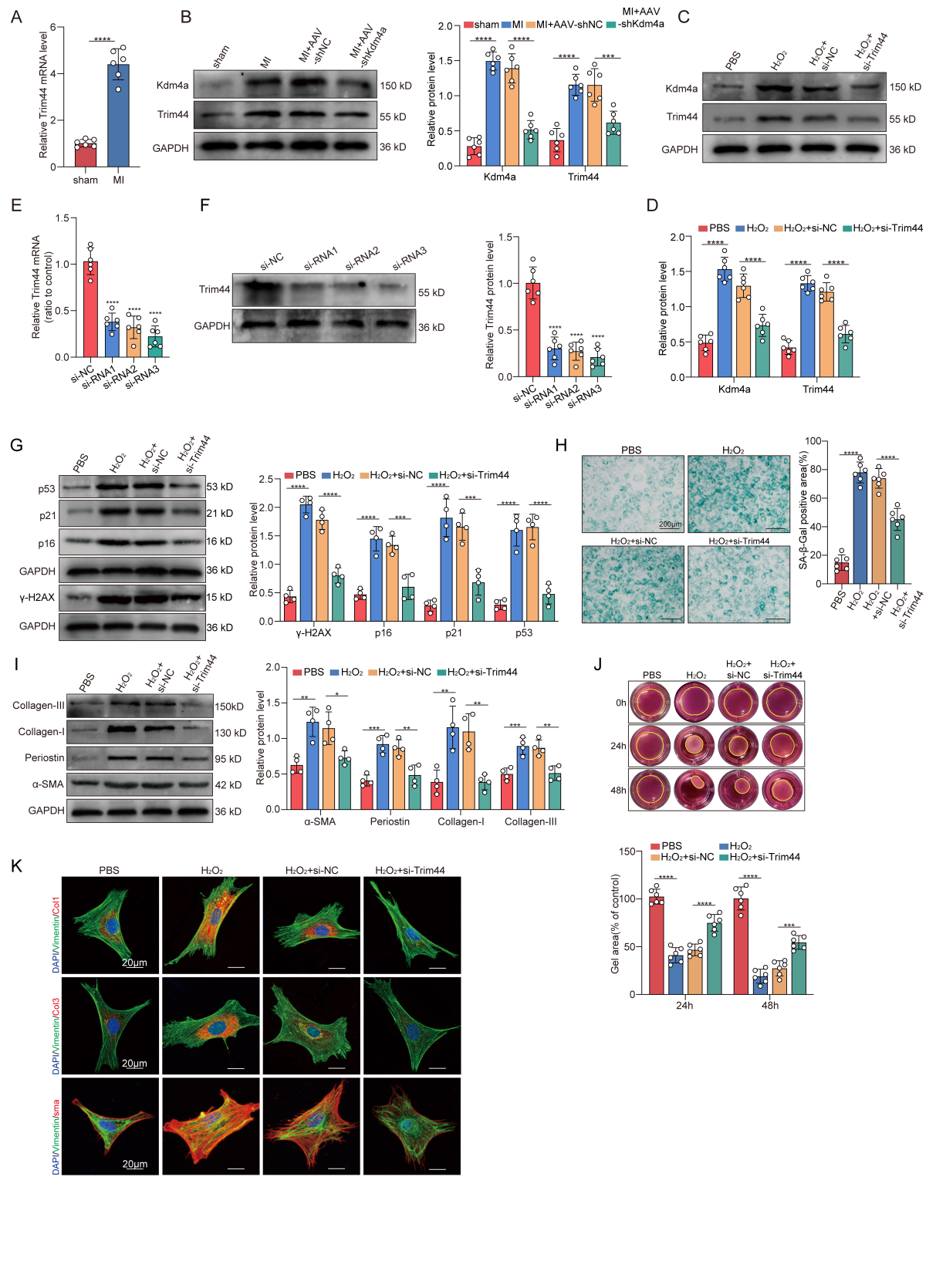
**

**Supplementary figure 11. The expression of Trim44 in heart after myocardial infarction and H_2_O_2_-induced fibroblasts.**

A. The mRNA expression of Trim44 was analyzed in adult mouse MI model hearts at 14 days post-MI. RNA levels were normalized to GAPDH. (n = 6/group)

B. Western blot analysis the expression of Kdm4a and Trim44 protein from an infarcted heart injected with AAV-shNC or AAV-shKdm4a at the 14 days after MI surgery. GAPDH was used as a loading control. (n = 6/group)

C-D. Western blot analysis and quantification the expression of Kdm4a and Trim44 protein levels in fibroblasts after Kdm4a inhibition. (n = 6/group)

E-F. RT-PCR and western blot analyzed the expression of Trim44 levels in fibroblasts incubated with three different Trim44-targeting siRNAs (si-Trim44). RNA levels were normalized to GAPDH. (n = 6/group)

G. Western blot analysis and quantification of p16, γH2AX, p21, and p53 protein expression in fibroblasts treated with PBS, H_2_O_2_, H_2_O_2_+ si-NC or H_2_O_2_+ si-Trim44. GAPDH was used as a loading control. (n = 4/group)

H. Representative images of SA-β-gal activity in primary cardiac fibroblasts treated with PBS, H_2_O_2_, H_2_O_2_+ si-NC or H_2_O_2_+ si-Trim44. And the percentage of positive fibroblasts was quantified. (n = 6 /group; scale bars = 200μm)

I. Western blot analysis and quantification of expression of fibrotic markers α-SMA, periostin, collagen I, and collagen III expressions in fibroblasts cocultured with fibroblasts from PBS, H_2_O_2_, H_2_O_2_+ si-NC or H_2_O_2_+ si-Trim44. GAPDH was used as a loading control. (n = 4/group)

J. Collagen gels containing fibroblasts were photographed at 0, 24, and 48 hours. The sizes of the gels were measured and analyzed to assess changes in the gel.

K. Representative immunofluorescence images for α-SMA (red), collagen I (red) and collagen III (red) in fibroblasts incubated with si-Trim44. (scale bars = 20 µm)

Data are expressed as the means SD for each group. A was analyzed by two-tailed unpaired t-test. B-K were analyzed by one-way ANOVA followed by Tukey’s test. J was analyzed by the two-way ANOVA followed by Tukey’s test. *P < 0.05, **P < 0.01, ***P < 0.001, ****P < 0.0001.

**Figure S12**

**
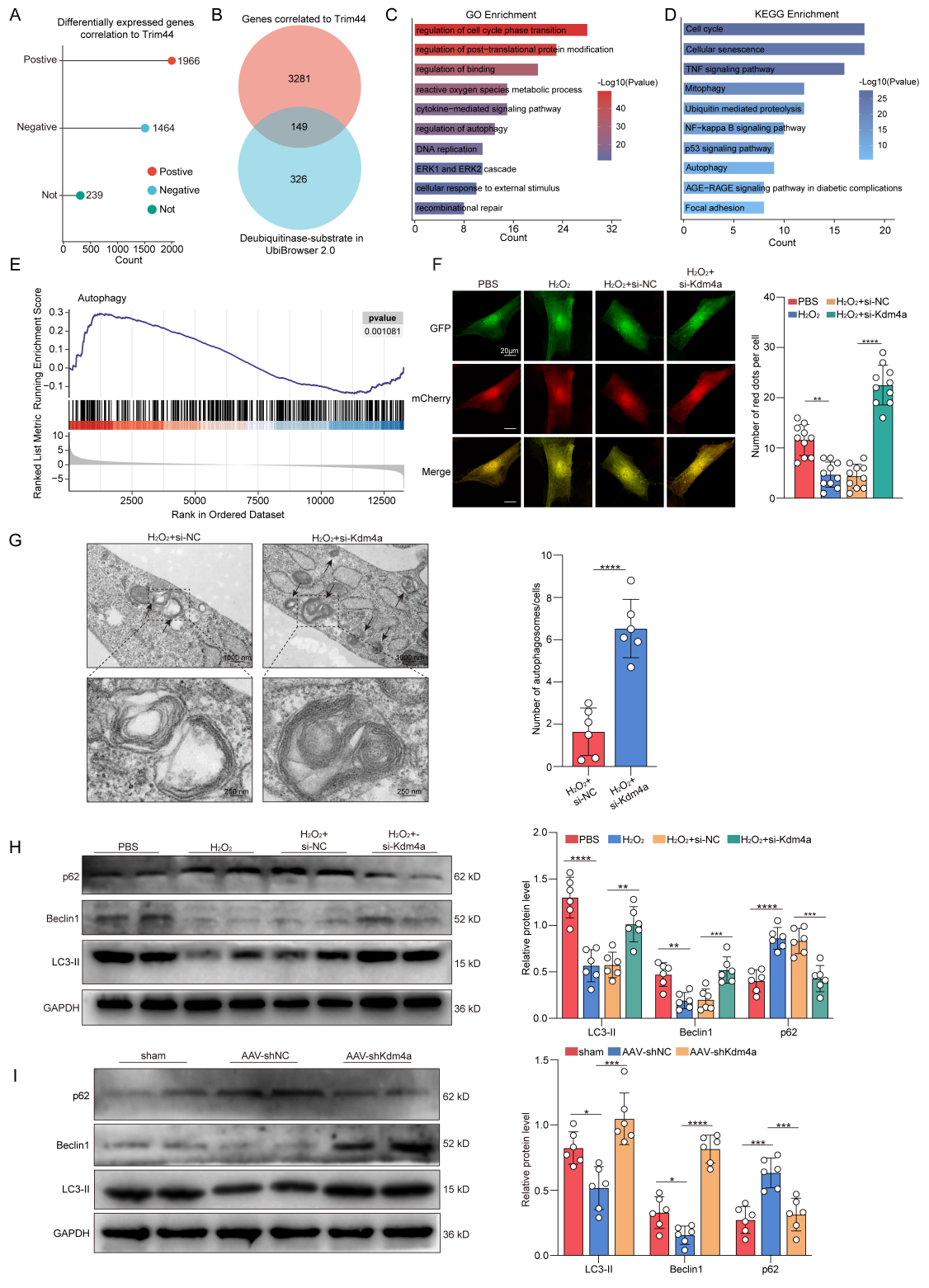
**

**Supplementary figure 12. Analysis of the relationship between senescence and autophagy in fibroblasts following Kdm4a knockdown.**

A. The number of differentially expressed genes correlated with Trim44 was assessed. Genes significantly correlated were identified with a p-value < 0.05 and an absolute value of the Pearson correlation coefficient > 0.8.

B. Venn diagram illustrating the intersection between significantly correlated genes with Trim44 and deubiquitinase-substrates in the UbiBrowser 2.0 database (<http://ubibrowser.ncpsb.org.cn>).

C-D. GO and KEGG pathway enrichment analysis of the overlapping 149 genes. The x-axis indicates the gene count, and the y-axis specifies the GO or KEGG terms.

E. GSEA of ‘Autophagy’ in H_2_O_2_-treated fibroblasts following Kdm4a knockdown. The x-axis represents the ranked gene list based on differential expression, while the y-axis displays the enrichment score.

F. H_2_O_2_-treated fibroblasts were pretreated with si-Kdm4a and then transfected with mCherry-GFP-LC3 adenovirus. LC3 spots were captured by confocal microscopy. (scale bars = 20 µm)

G. Representative transmission electron microscopy (TEM) images showed after treatment with si-Kdm4a in H_2_O_2_-treated fibroblasts. Arrowheads indicate autophagosomes. (scale bars = 1000 nm and 250nm)

H. Western blotting and densitometric analysis of the protein level of LC3-II, Beclin1 and p62 in each group. (n = 6 /group)

I. Western blot analysis of LC3-II, Beclin1 and p62 in cardiac tissue from mice harvested at 14 days post-MI in each group. (n = 6 /group)

Data are expressed as the means SD for each group. F, H and I were analyzed by one-way ANOVA followed by Tukey’s test. G was analyzed by two-tailed unpaired t-test. *P < 0.05, **P < 0.01, ***P < 0.001, ****P < 0.0001.

**Figure S13**

**
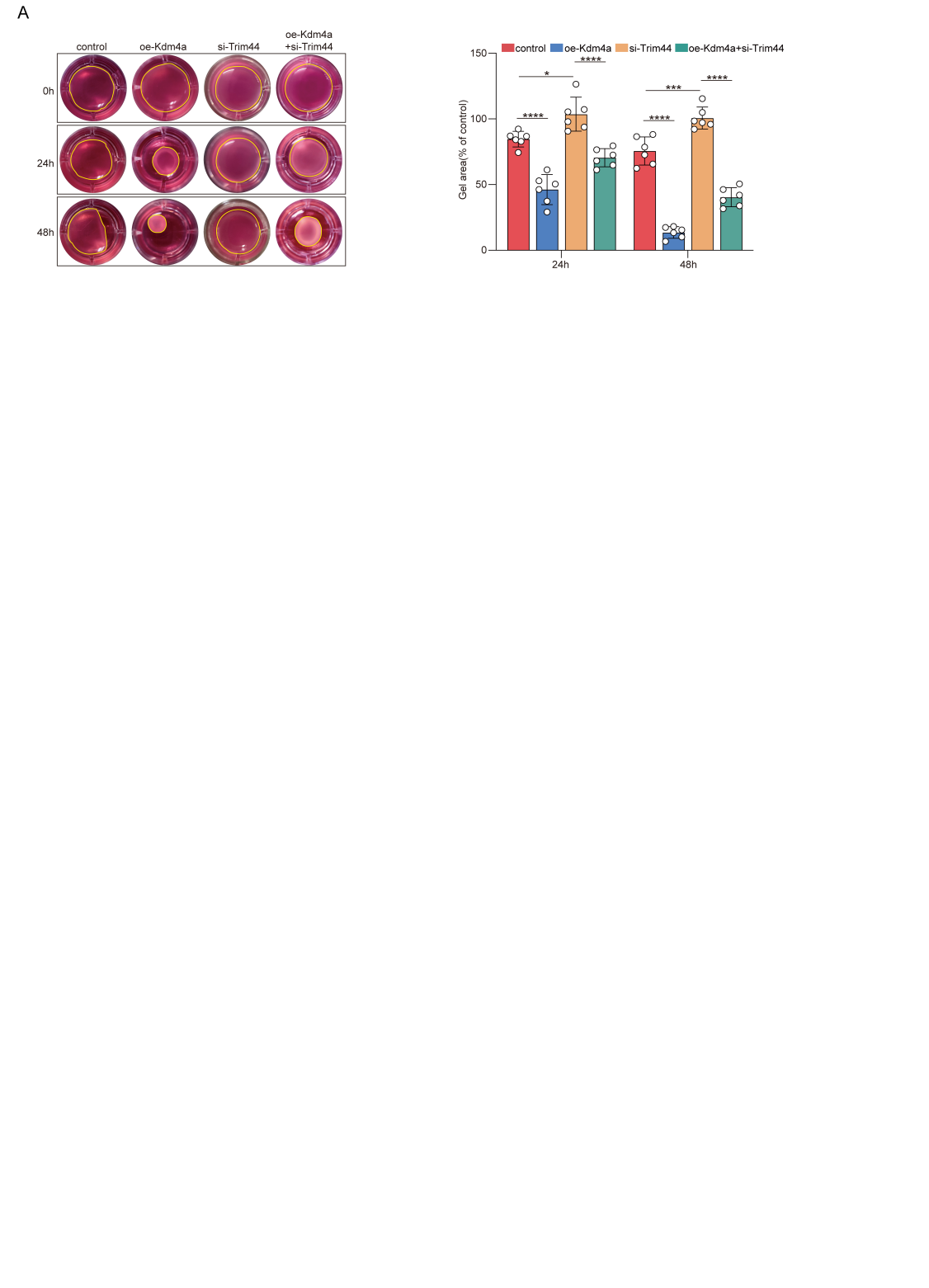
**

**Supplementary figure 13. Kdm4a knockdown attenuates fibroblast senescence by regulating Trim44-mediated autophagy.**

A. Collagen gels containing fibroblasts were photographed at 0, 24 and 48 hours. The sizes of the gels were measured and analyzed to assess changes in the gel.

Data are expressed as the means SD for each group. A was analyzed by the two-way ANOVA followed by Tukey’s test. *P < 0.05, **P < 0.01, ***P < 0.001, ****P < 0.0001.

**Figure S14**


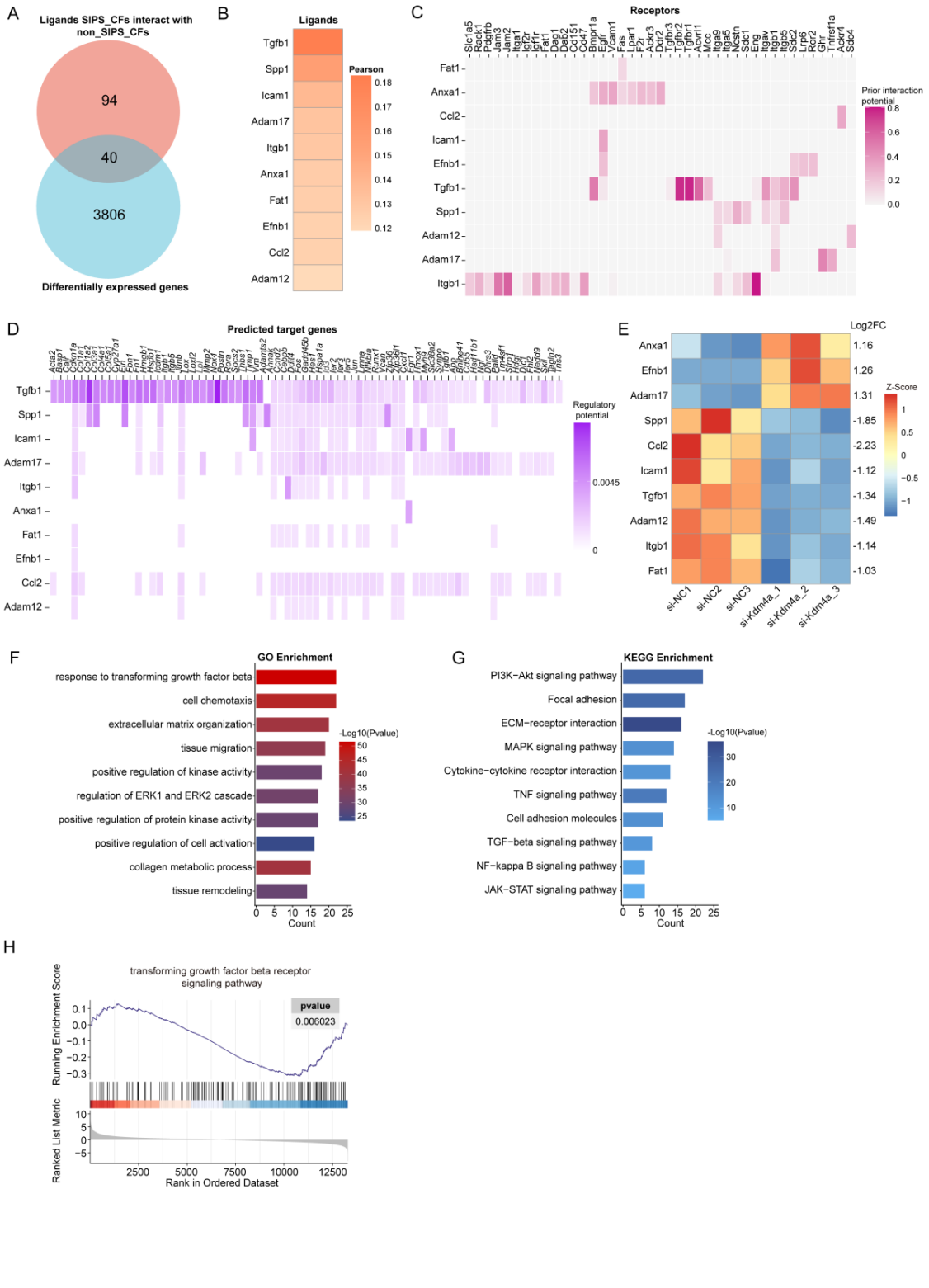


**Supplementary figure 14. Identification of the key SASP factor mediating cardiac fibroblast activation.**

A. Venn diagram showing the intersection of ligands SIPS fibroblasts to non-SIPS fibroblasts and differentially expressed genes in H_2_O_2_-treated fibroblasts following Kdm4a knockdown.

B. Heatmap showing the top 10 ligands with the highest Pearson correlation coefficients.

C. Heatmap showing the potential interaction of the ligands on the receptors.

D. Heatmap showing the potential regulation of the ligands on the receptors.

E. Heatmap showing the expression of the top 10 ligands in H_2_O_2_-treated fibroblasts transfected with si-NC or si-Kdm4a.

F-G. GO and KEGG pathway enrichment analysis of the top 10 ligands and their potential receptors and target genes. The x-axis indicates the gene count, and the y-axis specifies the GO or KEGG terms.

H. GSEA of ‘transforming growth factor beta receptor signaling pathway’ in H_2_O_2_-treated fibroblasts following Kdm4a knockdown. The x-axis represents the ranked gene list based on differential expression, while the y-axis displays the enrichment score.

**Figure S15**


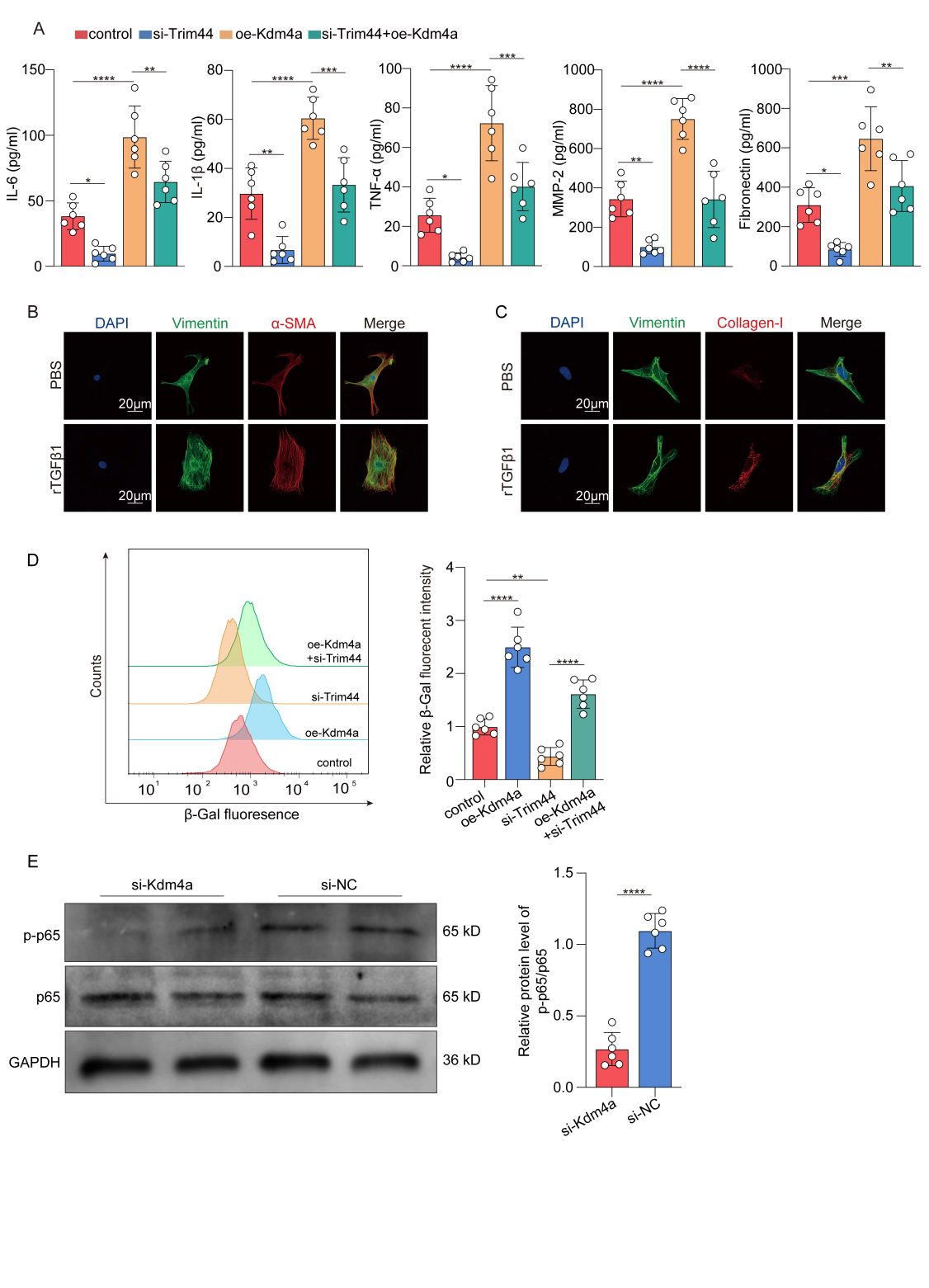


**Supplementary figure 15. Kdm4a deficiency through** NF-κB signaling pathway **inhibits SASP secretion.**

A. SASP were detected by enzyme-linked immunosorbent assay (ELISA). ELISAs were performed on conditioned medium collected from fibroblasts incubated in OE-NC + si-NC, OE-Kdm4a+ si-NC, OE-NC + si-Trim44 or OE-Kdm4a+ si-Trim44. (n = 6/group)

B-C. Representative images of α-SMA and collagen I immunostaining in fibroblasts

treated with PBS or rTGF-β1. (scale bars = 20 µm)

D. Flow cytometry sort the number of senescent fibroblasts using β-galactosidase. (n = 6 /group)

E. Western blot analysis p65 and p-p65 in fibroblasts treated with si-NC or si-Kdm4a. (n = 6 /group)

Data are expressed as the means SD for each group. A and D was analyzed by one-way ANOVA followed by Tukey’s test. E was analyzed by two-tailed unpaired t-test. *P < 0.05, **P < 0.01, ***P < 0.001, ****P < 0.0001.

**Figure S16**


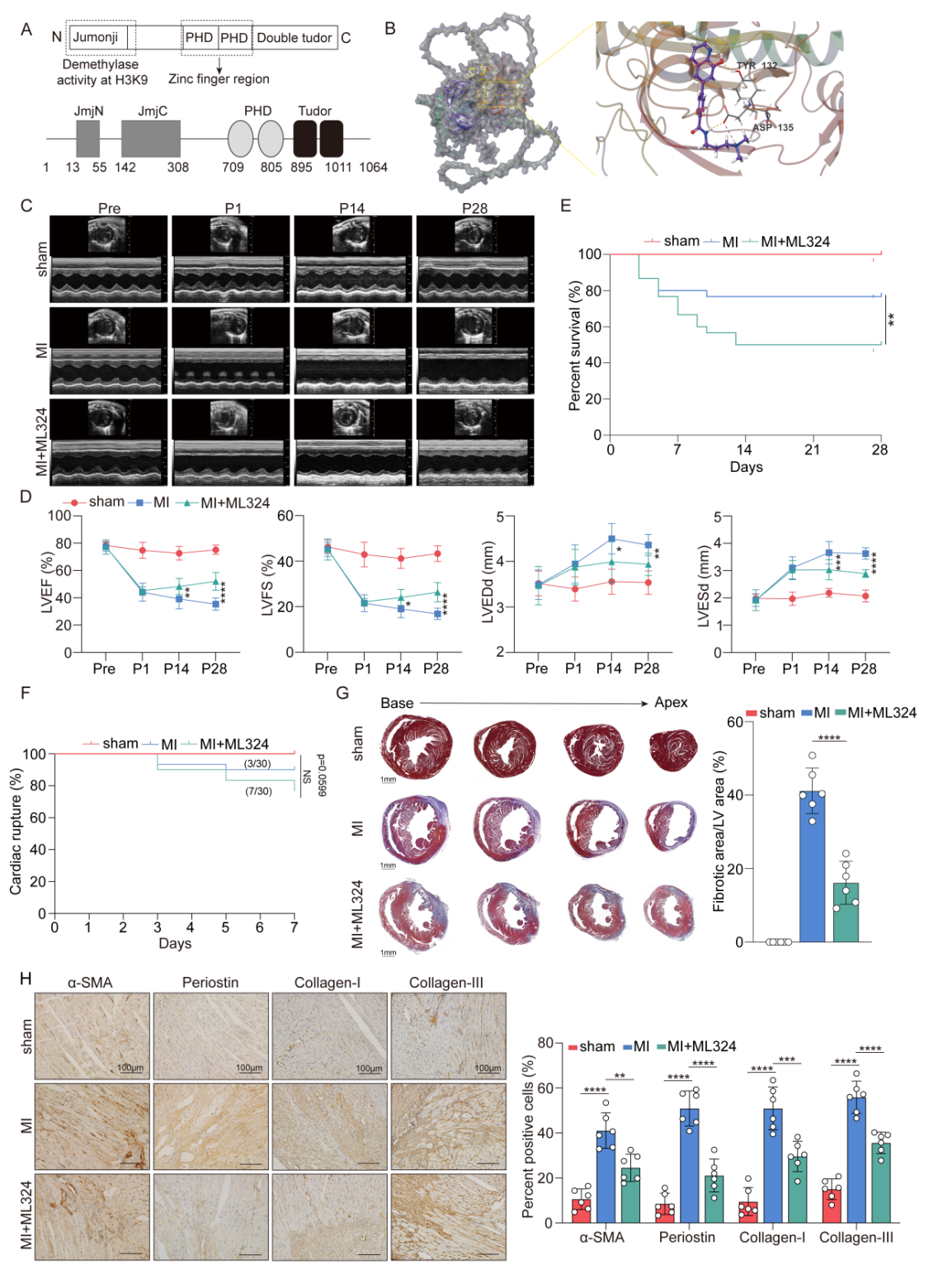


**Supplementary figure 16. Pharmacological inhibition of Kdm4a promotes cardiac function after MI in adult hearts**

1. Schematic diagram illustrating the structure of the Kdm4a protein.
2. Molecular docking of the ML324 into the Kdm4a.

C-D. Cardiac function was analyzed by echocardiography at pre-MI and 1, 14 and 28 days post-MI treated with ML324. (n = 10 /group)

E. Kaplan-Meier survival curves in the sham, MI and MI+ML324 groups post-MI. (n =30 /group)

F. Percentage cardiac rupture between mice injected with PBS or ML324 post-MI. (n=30 /group)

G. Masson's trichrome staining and quantification of infarct size in cross sections of adult mouse post-MI treated with ML324. (n = 6 /group; scale bars = 1 mm)

H. Representative immunohistochemical staining of α-SMA, periostin, collagen I and collagen III in transverse sections from adult mouse MI model hearts at 14 days after MI treated with ML324. (n = 6 /group; scale bars = 100 μm)

Data are expressed as the means SD for each group. D was analyzed by the two-way ANOVA followed by Tukey’s test. E and F was analyzed by the log-rank (Mantel-Cox) test. G and H were analyzed by one-way ANOVA followed by Tukey’s test. *P < 0.05, **P < 0.01, ***P < 0.001, ****P < 0.0001.

**Figure S17**


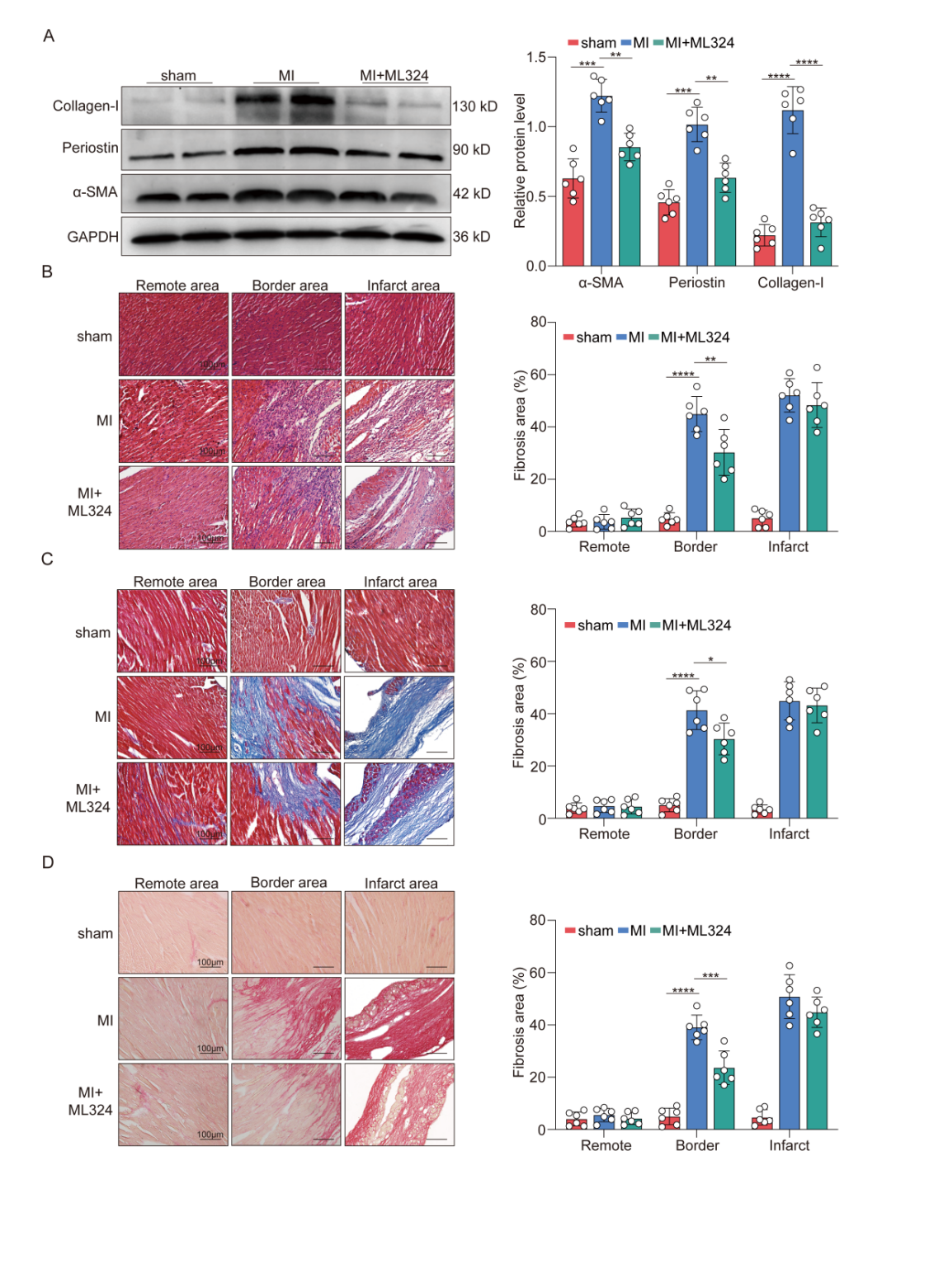


**Supplementary figure 17. Pharmacological inhibition of Kdm4a improves cardiac fibrosis after MI in adult hearts**

1. Western blot analysis and quantification of α-SMA, periostin and collagen I protein levels in heart tissues of mice after MI treated with ML324. (n = 6 /group)

B-D. The myocardium from three different zones of mice was harvested on day 28 post-MI for the following study. Representative images of haematoxylin and eosin (H&E), picrosirius red staining and Masson trichrome staining in the mouse heart. (n = 6 /group; scale bars = 100 μm)

Data are expressed as the means SD for each group. A-D were analyzed by one-way ANOVA followed by Tukey’s test. *P < 0.05, **P < 0.01, ***P < 0.001, ****P < 0.0001.

**Western Blot images**


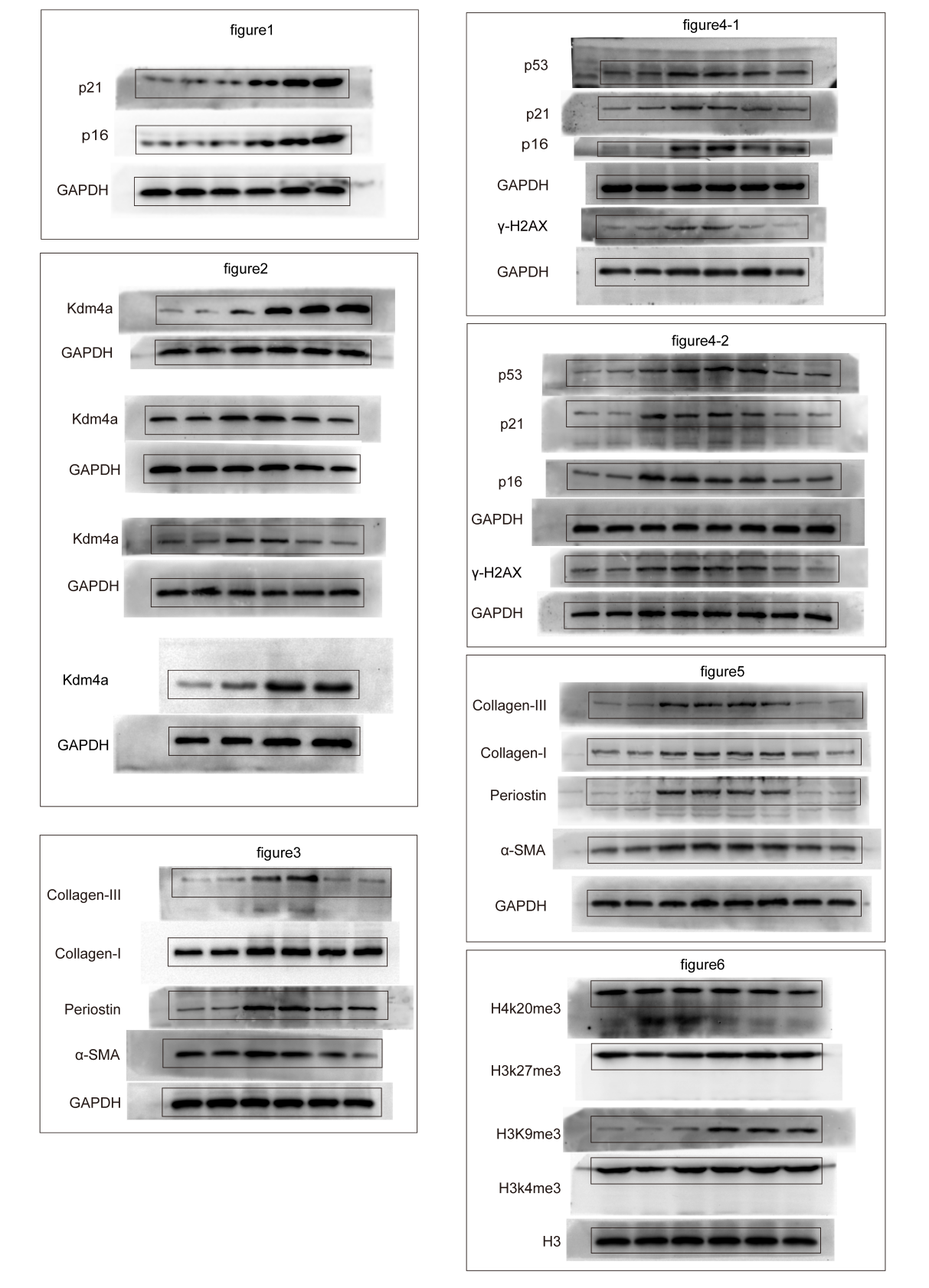


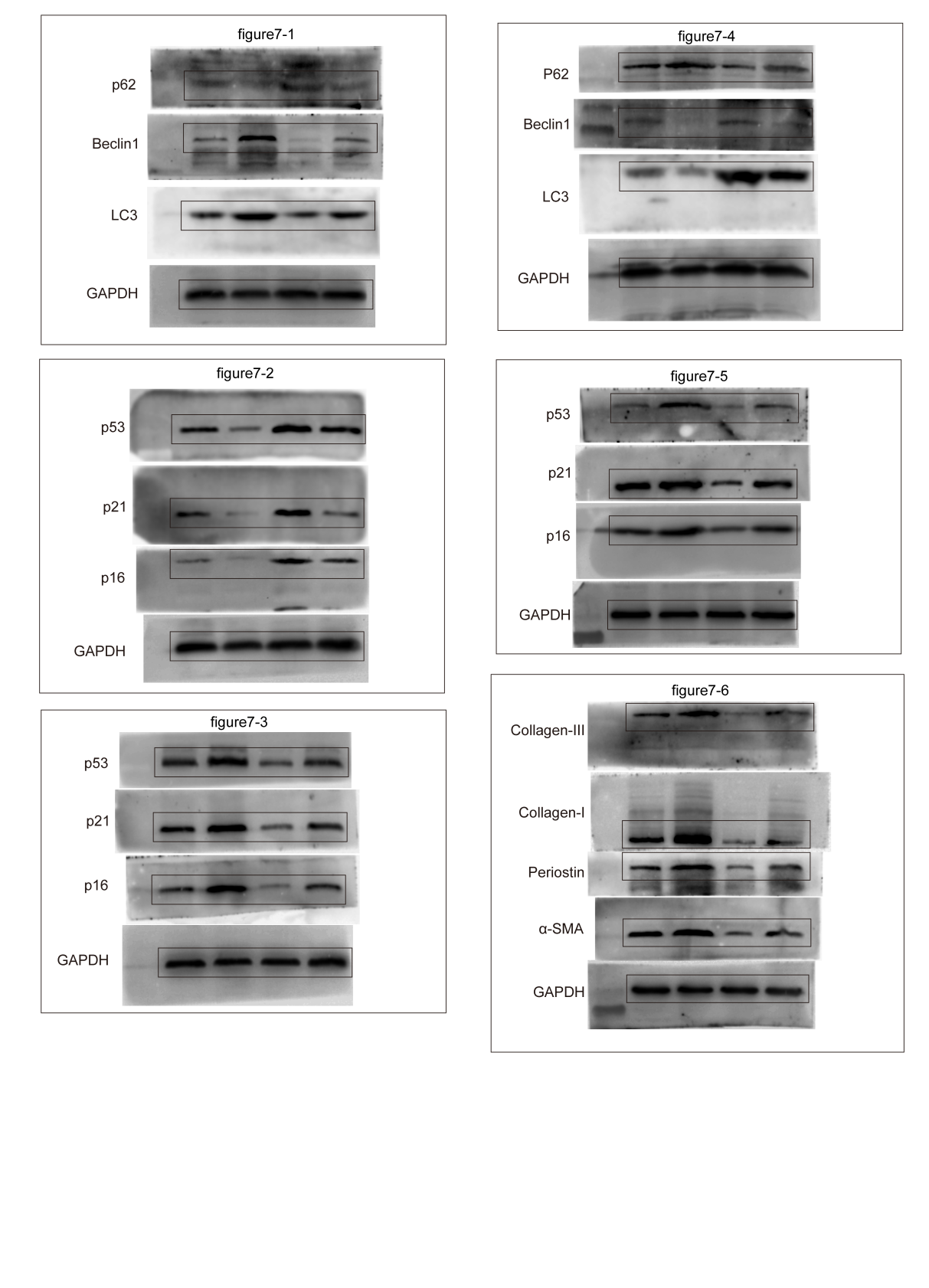


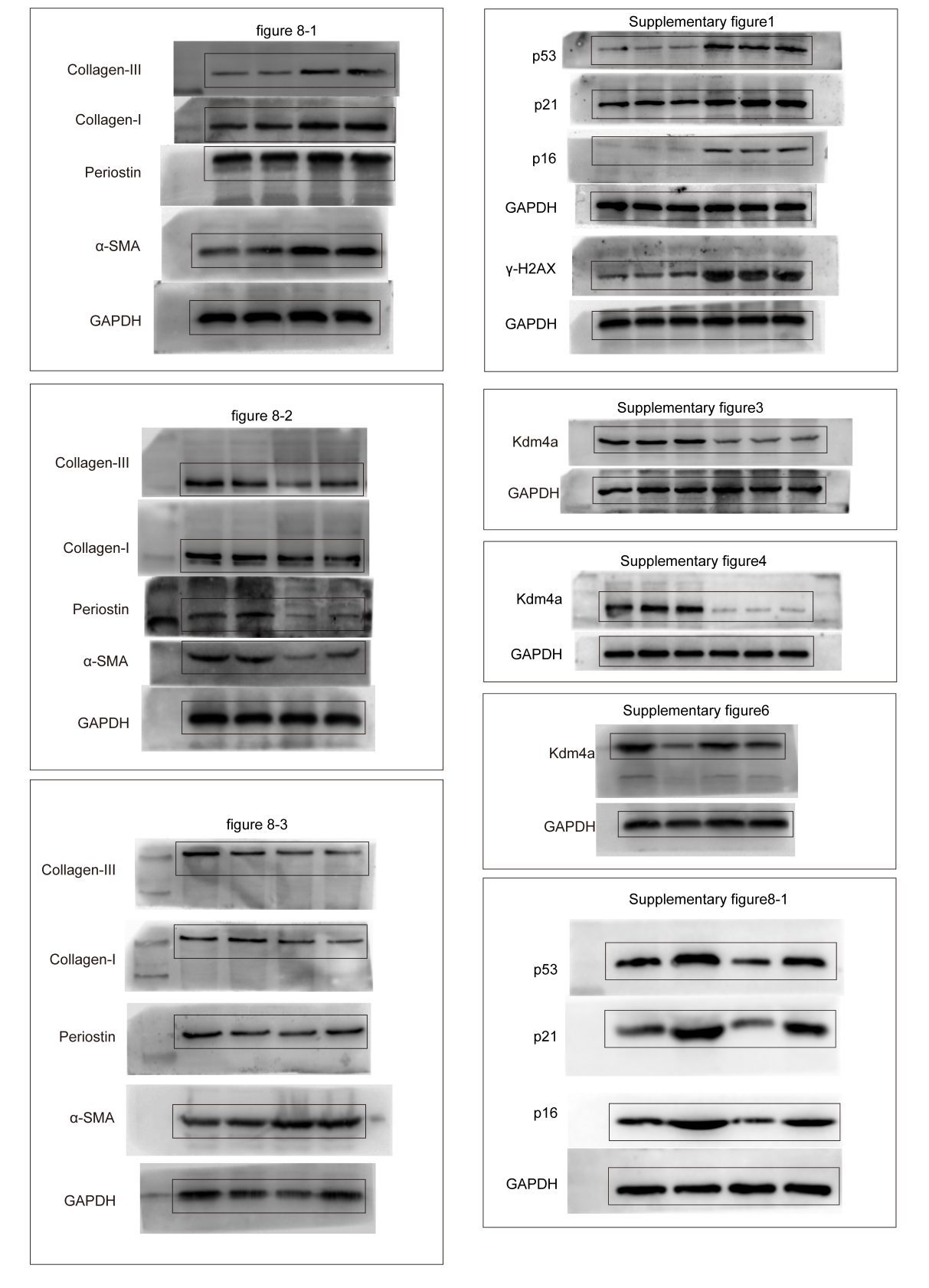


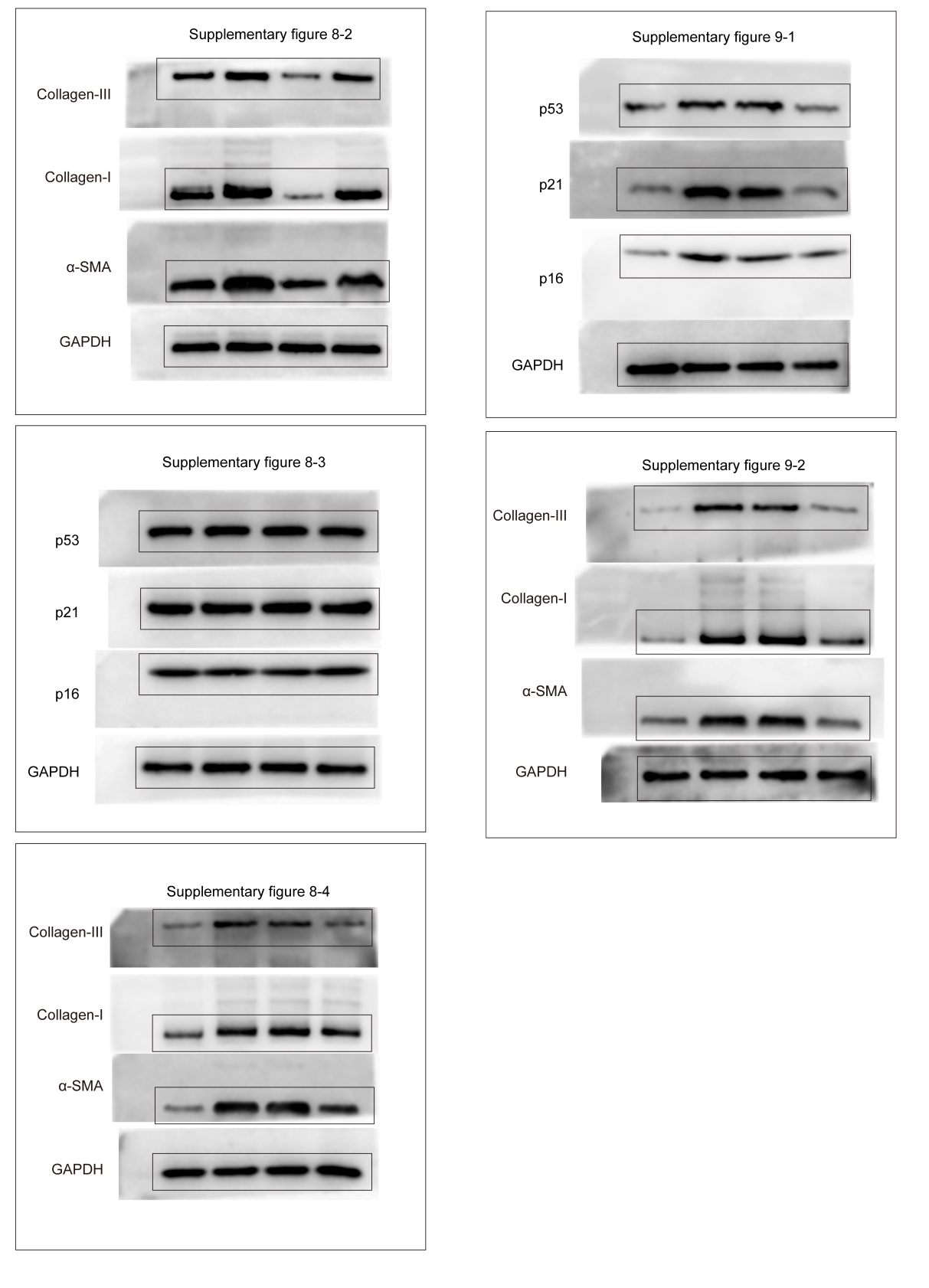


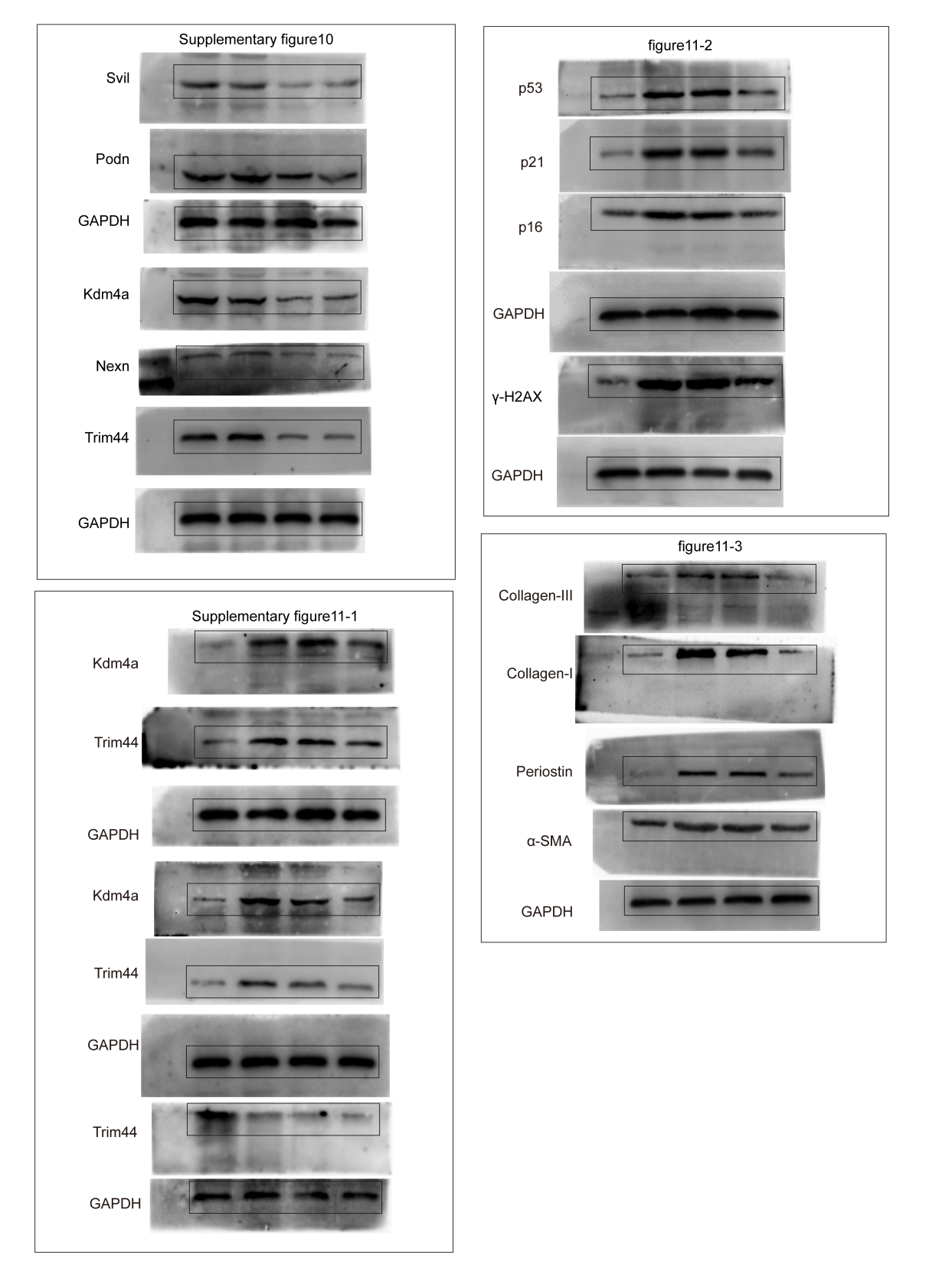

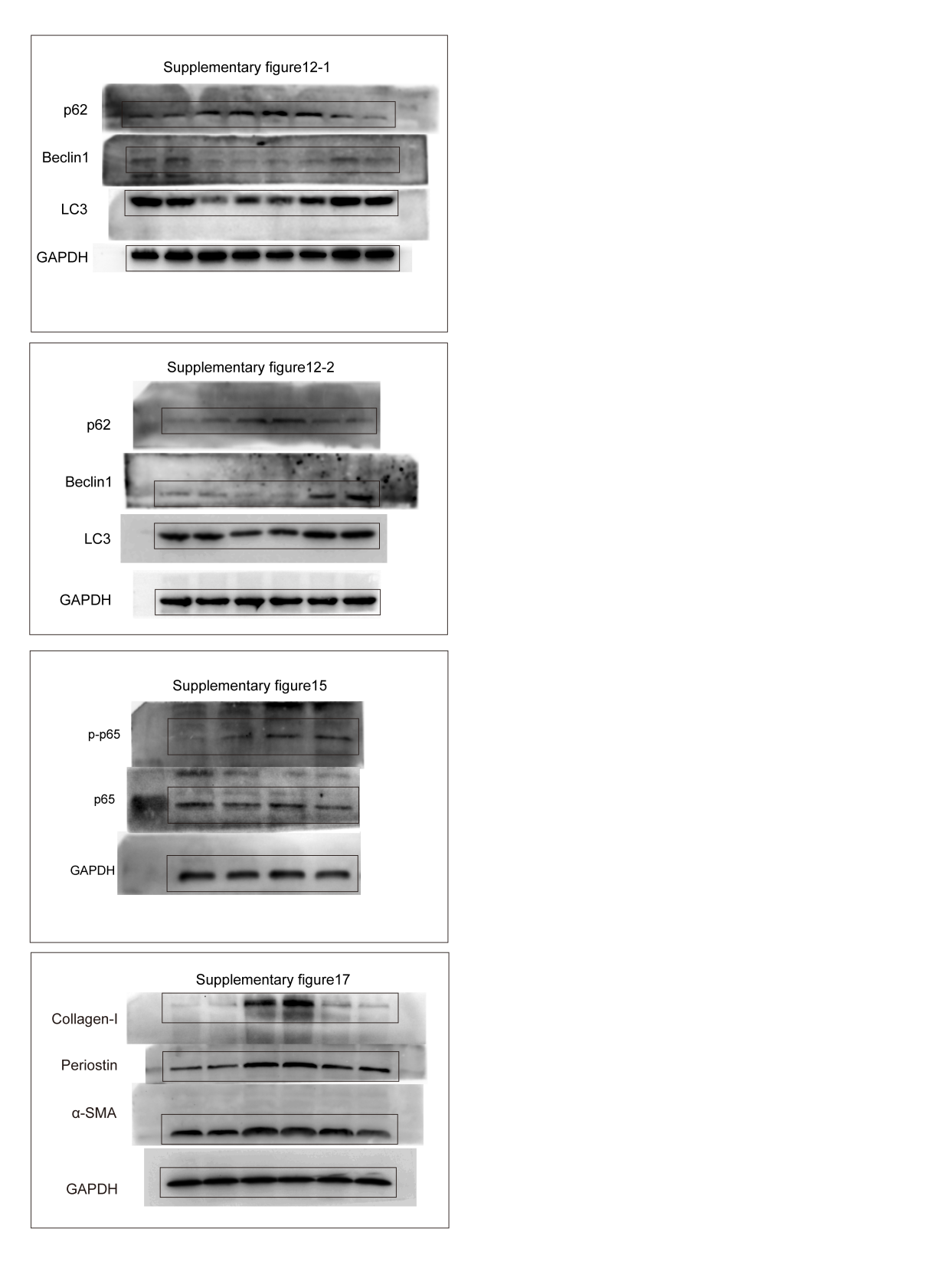

Supplement: Supplementary file 1 — Supporting Information [file ADVS-12-2414830-s001.docx]
